# Gender differences in fibrosis remodeling in patients with long-standing persistent atrial fibrillation

**DOI:** 10.18632/oncotarget.16342

**Published:** 2017-03-17

**Authors:** Zhi Li, Zengwei Wang, Zongtao Yin, Yuji Zhang, Xiaodong Xue, Jinsong Han, Yan Zhu, Jian Zhang, Maximilian Y. Emmert, Huishan Wang

**Affiliations:** ^1^ Department of Cardiovascular Surgery, Shenyang Northern Hospital, Shenyang, China; ^2^ Clinic for Cardiovascular Surgery, University Hospital Zurich, Rämistrasse, Zürich, Switzerland

**Keywords:** gender differences, atrial fibrillation, remodeling, mitral valve, pulmonary vein

## Abstract

The success rate of catheter ablation in atrial fibrillation (AF) is known to be lower in females than in males. However, while the exact mechanism for this phenomenon remains to be elucidated, tissue fibrosis may play an important role in this regard. It has been shown that fibrosis promotes AF and its recurrence, thereby substantially reducing the efficacy of catheter ablation in AF patients. Thus, we hypothesized that fibrosis may contribute to gender differences in the outcomes of AF catheter ablation.

Here we systematically assessed pulmonary vein sleeves obtained from 166 patients with and without long-standing persistent-AF (LSP-AF) in order to identify gender-specific mechanistic differences in fibrosis remodeling of AF patients.

Histological analysis revealed that the female LSP-AF group, rather than its male counterpart, had a higher degree of fibrosis when compared to the NON-AF group. Further analysis using microarray, immunohistochemistry and Western Blot displayed that gender differences in fibrosis remodeling of LSP-AF were mainly due to the inherent differential expression of fibrosis-related genes (n=32) and proteins (n=6). Especially, those related to the TGFβ/Smad3 pathway appeared to be up-regulated in the female LSP-AF group thus promoting an aggravation of fibrosis remodeling. In summary, our data suggest that the aggravation of fibrosis remodeling in women may be an important reason for the low success rate of AF catheter ablation when compared to men. Therefore, inhibiting the TGFβ/Smad3 pathway-mediated fibrosis could represent an interesting target for future therapeutic concepts to improve the success rate of AF catheter ablation in women.

## INTRODUCTION

Atrial fibrillation (AF) is one of the most prevalent arrhythmias worldwide and it is related to an increased cardiovascular morbidity and mortality [1]. However, so far, gender-specific differences in AF patients are widely neglected, and substantial knowledge gaps do exist in the understanding of epidemiology, pathophysiology, presentation, and prognosis between men and women [2].

Recent studies have suggested that the incidence, clinical presentation and consequences of AF are different between genders. For instance, while men are found to have a 1.5-fold higher risk of developing AF than women, but this gap appears to become balanced with advancing age due to the longer life-span of women [3]. In contrast, more comorbidities such as heart failure or stroke are usually found in women [4].

With regards to the treatment of AF patients, catheter-ablation represents a reasonable therapy strategy for both genders. However, interestingly, women are more likely to experience higher frequency of recurrence when compared to men [5]. While the exact mechanism for this phenomenon remains to be elucidated, fibrosis has been suggested to play a key role in this regard. Fibrosis remodeling leads to a dissociation in atrial conduction and thereby promotes AF which then ultimately results in a lower efficacy of ablation therapy. [6]. Next, it has been suggested that the more extensive the fibrosis is, the more ineffective the ablation therapy becomes [7].

Therefore, we hypothesized that fibrosis may play an important role in the less effective AF ablation outcomes in women when compared to men. Subsequently, in the present study we comprehensively assessed pulmonary vein sleeves obtained from 166 patients with and without long-standing persistent-AF (LSP-AF) in order to identify gender-specific mechanistic differences in fibrosis remodeling of AF patients.

## RESULTS

### Patients characteristics

Patient characteristics were summarized in Table 1 and Supplementary Table 1. In regard to the gender comparison there were no differences between women and men for in left atrial enlargement ratio, age and hypertension (Supplementary Table 1). Comparison in regard to AF and NON-AF also displayed no significant gender differences in regard to age, clinical diagnosis, accompanying disease and echocardiographic parameters (Table 1).

**Table 1:** Clinical characteristics of study population

Classification	Female		*P*-value	Male		*P*-value
	NON-AF (*n*=30)	LSP-AF (*n*=55)		NON-AF (*n*=35)	LSP-AF (*n*=46)	
Age, y	58.9±6.1	57.7±6.0	0.38	56.6±5.4	57.0±5.3	0.75
**Clinical diagnosis (n%)**						
MS+MR	15(50%)	39(70.91%)	0.06	5(14.29%)	15(32.61%)	0.06
MR	14(46.67%)	15(27.27%)	0.07	29(82.86%)	30(65.22%)	0.08
MS	1(3.33%)	1(1.82%)	0.66	1(2.86%)	1(2.17%)	0.84
**Accompanied disease (n%)**						
AVD	9(30%)	20(36.36%)	0.55	14(40%)	14(30.43%)	0.37
TVD	4(13.33%)	18(32.73%)	0.05	7(20%)	18(39.13%)	0.06
CHF	0(0%)	2(3.64%)	0.29	0 (0%)	0(0%)	
CHD	7(23.33%)	21(38.18%)	0.16	8(22.86%)	15(32.61%)	0.34
Cerebral infarction	4(13.33%)	8(14.55%)	0.89	2(5.71%)	1(2.17%)	0.40
Left atrial thrombus	0(0%)	6(10.91%)	0.06	8(22.86%)	4(8.70%)	0.08
Pulmonary hypertension	3(10%)	5(9.09%)	0.89	6(17.14%)	12(26.09%)	0.34
Hyperthyroidism	2(6.67%)	0(0%)	0.05	1(2.86%)	1(2.17%)	0.84
Gout	0(0%)	0(0%)		0(0%)	1(2.17%)	0.38
Hypertension						
I	0(0%)	1(1.82%)	0.46	0(0%)	1(2.17%)	0.38
II	1(3.33%)	1(1.82%)	0.66	4(11.43%)	2(4.35%)	0.23
III	6(20%)	10(18.18%)	0.84	6(17.14%)	6(13.04%)	0.61
**Echocardiography**						
LAD(mm)	48.47±5.95	48.22±5.35	0.84	49.66±5.21	51.78±5.02	0.07
LAER	1.28±0.16	1.27±0.14		1.24±0.13	1.30±0.13	
LVEF	0.59±0.09	0.58±0.07	0.80	0.57±0.06	0.57±0.06	0.95
LVFS	0.28±0.04	0.28±0.04	0.60	0.29±0.05	0.28±0.04	0.46
LVEDD(mm)	52.10±5.28	49.69±5.73	0.06	56.44±6.35	54.22±6.16	0.12

Data is presented as means±SEM. LSP-AF, long standing persistent atrial fibrillation; MS, mitral stenosis; MR, mitral regurgitation; AVD, Aortic valve disease; TVD, tricuspid valve disease; CHF, congestive heart failure;CHD, coronary heart disease; LAD, left atrial diameter; LAER, left atrial enlargement ratio: female, LAD/38;male, LAD/40; LVEF, left ventricular ejection fraction; LVFS, left ventricular fractional shortening; LVEDD, left ventricular end diastolic dimension; *P*-value (female: NON-AF vs LSP-AF; male: NON-AF vs LSP-AF).

### Collagen volume fraction (CVF)

**Figure 1:**
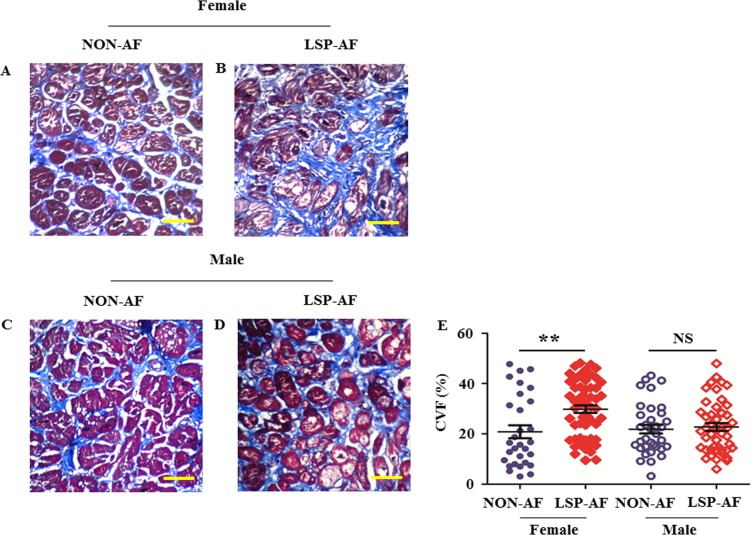
Gender difference in collagen volume fraction (CVF) (X60, bar = 50 um) Female group: NON-AF, *n* = 30(blue solid circle); LSP-AF, *n* = 55(red solid diamond); Male group: NON-AF, *n* = 35(blue hollow circle); LSP-AF, *n* = 46(red hollow diamond). **A**. and **B**., Masson staining of pulmonary vein sleeves from female NON-AF and LSP-AF group. **C**. and **D**., Masson trichrome of pulmonary vein sleeves from male NON-AF and LSP-AF group. Collagen (blue), Cardiac muscle (red), Nuclei (black). **E**., Comparative analysis of CVF in Female group between NON-AF and LSP-AF group. *P* ^*^ < 0.01; Comparative analysis of CVF in Male group between NON-AF group and LSP-AF group. P^NS^ > 0.05.Y axis, CVF (100%) = collagen area/total area. LSP-AF, long-standing-persistent AF. All data are expressed as means ± SEM. Unpaired *t*-test.

To assess the degree of fibrosis remodeling, CVF was assessed in representative sections from each group by Masson's trichrome staining and shown in Figure 1A-1D. CVF was significantly increased (Figure 1E, *P* = 0.0019 < 0.01) in the female LSP-AF group (Figure 1B, CVF = 29.83% ±11.22%) when compared to the female NON-AF group (Figure 1A, CVF = 20.82%± 14.27%). In contrast, it did not differ significantly (Figure 1E, *P*= 0.7196) between the male LSP-AF group (Figure 1D, CVF = 22.73% ±10.42%) and the corresponding NON-AF male group (Figure 1C, CVF = 21.90% ±10.20%). These findings suggested, an aggravation of fibrosis remodeling in the female LSP-AF group but not in its male counterpart. Further analysis revealed there was a positive correlation between fibrosis remodeling and LSP-AF in females (Pearson r = 0.3319, *P* < 0.01), but not in males (Pearson r = 0.04050, *P* = 0.7196) (Supplemental Table 2).

### Receiver operating characteristic (ROC) curve analysis

**Figure 2:**
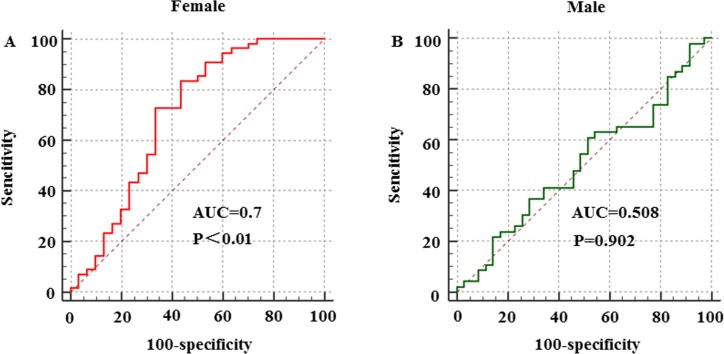
Gender difference in CVF to predict the presence of LSP-AF Female group: NON-AF, *n* = 30; LSP-AF, *n* = 55; Male group: NON-AF, *n* = 35; LSP-AF, *n* = 46; **A**. and **B**., Receiver operating characteristic (ROC) curves of gender difference in CVF to predict the presence of LSP-AF. AUC indicates area under ROC curve.

Next, ROC curve analysis was performed to evaluate the utility of CVF in male and female subjects to predict the occurrence of LSP-AF (Figure 2). The results revealed statistical significance in females with an optimal cut-off value for female CVF to predict the presence of LSP-AF of was > 16.72%, with 56.7% specificity and 83.6% sensitivity (AUC = 0.7, 95% CI: 0.591-0.795, *P* < 0.01), while there was no statistical significance in male CVF to predict the presence of LSP-AF (AUC = 0.508, 95% CI: 0.395-0.621, *P* = 0.902). Thus, when compared with male CVF, the female CVF showed good predictive values for the presence of LSP-AF.

### Dysregulated mRNA expression profiles in LSP-AF

**Figure 3:**
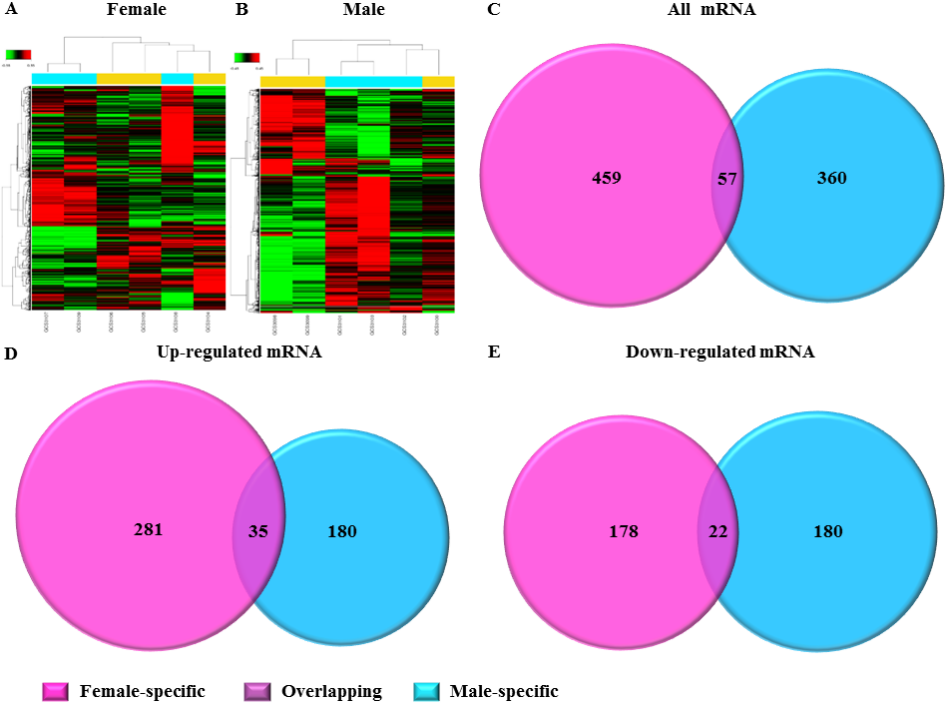
Gender differences in dysregulated AF-related genes **A**. and **B**., The hierarchical cluster analysis of differentially expressed mRNAs associated with LSP-AF between female group and male group (*n* = 3/group). The relative gene log2 expression changes are expressed by a color gradient intensity scale, as shown in the top left corner. Green color indicates down-regulation, and red color indicates up-regulation of mRNA expression. Each row represents a separate sample and each column represents a single mRNA. Comparative analysis of dysregulated Total mRNA **C**., up-regulated mRNA **D**., and down-regulated mRNA **E**. in LSP-AF group between female and male group. Pink, Female-specific part; Purple, overlapping part, Blue, Male-specific part.

Microarray was performed to assess for gender differences in dysregulated mRNA expression profiles in patients with LSP-AF and results showed systematic variations between females and males respectively (Figure 3A and 3B). In the female LSP-AF group, a total of 516 differentially expressed mRNAs (316 upregulated, 200 downregulated) (Supplemental Table 3) were found, while in the male LSP-AF group, 417 differentially expressed mRNAs were detected (215 upregulated, 202 downregulated) were found (Supplemental Table 4). Further analysis revealed that in the LSP-AF groups, only 57 mRNAs (35 upregulated, 22 downregulated) were overlapping between genders (Figure 3C) (Supplemental Table 6), while most mRNAs appeared to be gender-specific with 459 female-specific mRNAs (281 upregulated, 178 downregulated) (Supplemental Table 5) and 360 male-specific mRNAs (180 upregulated, 180 downregulated) (Figure 3D and 3E) (Supplemental Table 7) suggesting that there were substantial gender differences in dysregulated mRNA expression profiles in patients with LSP-AF.

### Categories of AF-related genes by GO and pathway analysis

Gene ontology (GO) analysis is the key functional classification method used at NCBI and can organize genes into hierarchical categories and uncover gene regulatory networks on the basis of biological processes and molecular functions. Pathway Analysis is used to identify canonical pathways representing genes. In a further step, GO and Pathway analysis were applied to classify differentially expressed AF related mRNAs.

### Gender differences are displayed in categories of AF-related gene (Figure 4A and 4B) (Supplemental Table 8-11)

- female-specific: GO, 183(Supplemental Figure 1); Pathway 31(Supplemental Figure 2)
- overlapping: GO, 178(Supplemental Figure 3); Pathway, 98(Supplemental Figure 4)
- male-specific: GO, 200(Supplemental Figure 5); Pathway 34(Supplemental Figure 6)

Representative top5 categories of AF-related gene are shown as follows:

### GO analysis: (Figure 4C)

- female-specific group: immune response; inflammatory response; blood coagulation
- overlapping group: small molecule metabolic process; cell adhesion
- male-specific group: respiratory electron transport chain; extracellular matrix (ECM) organization; Signal transduction

### Pathway analysis (Figure 4D)

- female-specific group: Focal adhesion; PI3K-Akt signaling pathway; Malaria; Staphylococcus aureus infection
- overlapping group: Metabolic pathways

male-specific group: Huntington's disease; Parkinson's disease; Alzheimer's disease; Oxidative phosphorylation.

**Figure 4:**
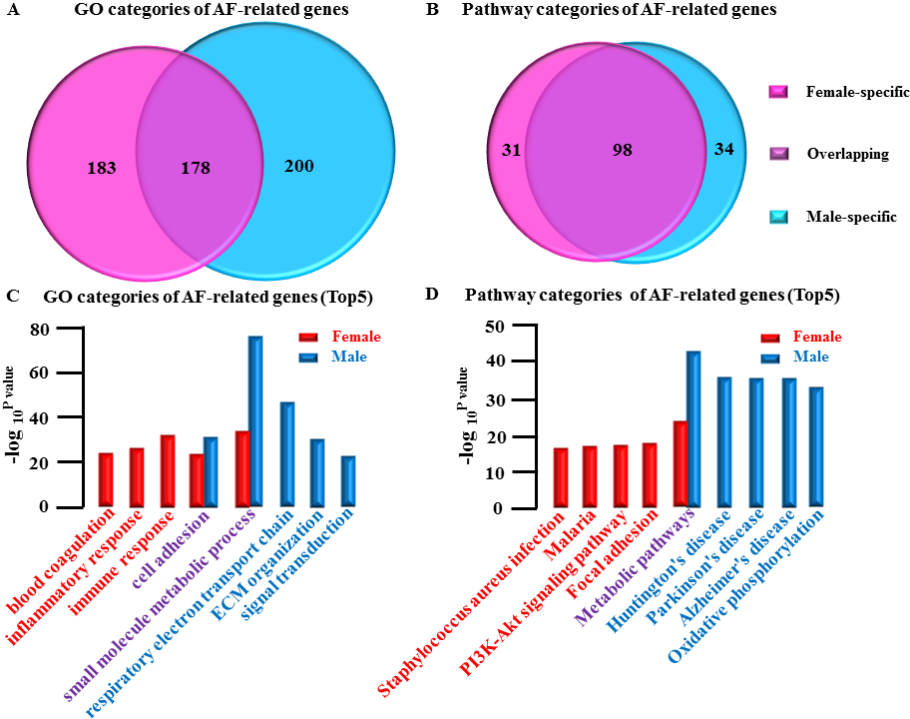
Gender differences in categories of AF-related genes **A**., Comparative analysis of GO categories of AF-related genes between female LSP-AF and male LSP-AF group. **B**., Comparative analysis of Pathway categories of AF-related genes in LSP-AF group between female group and male group. Pink, Female-specific part; Purple, overlapping part, Blue, Male-specific part. **C**., GO categories of AF-related genes (Top5): Female-specific categories (red bar): immune response; inflammatory response; blood coagulation; Overlapping categories (red and blue bar): small molecule metabolic process; cell adhesion; male-specific categories (blue bar): respiratory electron transport chain; extracellular matrix (ECM) organization; Signal transduction. Y axis, −log_10_^P-value^;X axis, GO categories of AF-related genes; **D**., Pathway categories of AF-related genes (Top5):Female-specific categories (red bar):Focal adhesion; PI3K-Akt signaling pathway;Malaria;Staphylococcus aureus infection; Overlapping categories (red bar and blue): Metabolic pathways; Male-specific categories (blue bar): Huntington's disease; Parkinson's disease; Alzheimer's disease; Oxidative phosphorylation. Y axis, −log_10_^P-value^; X axis, Pathway categories of AF-related genes.

In summary we found that the most obvious category for both genders was metabolism-related suggesting that those categories may play an important regulatory role in AF (Figure 4C and 4D).

### Categories of fibrosis remodeling related genes in LSP-AF

**Figure 5:**
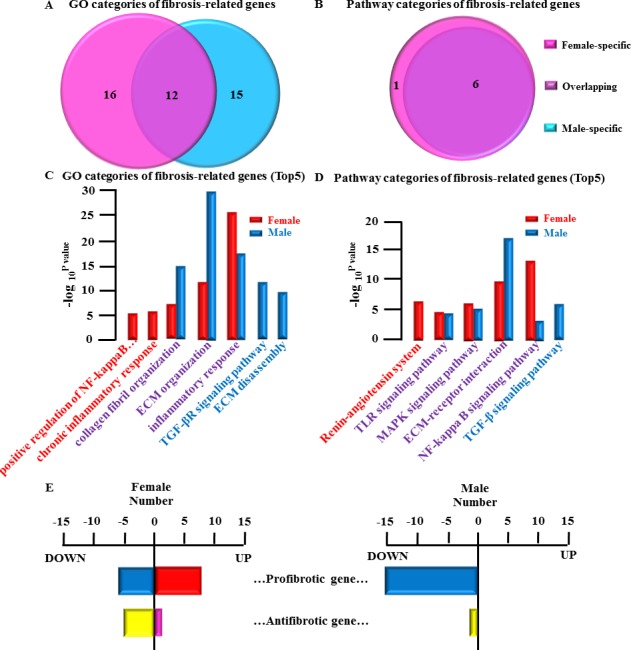
Gender differences in fibrotic-remodeling-related genes and their categories **A**., Comparative analysis of GO categories of fibrosis-related genes between female LSP-AF and male LSP-AF group. **B**., Comparative analysis of Pathway categories of AF-related genes between female LSP-AF and male LSP-AF group. Pink, Female-specific part; Purple, overlapping part, Blue, Male-specific part. **C**., GO categories of fibrosis-related genes (Top5) : Female-specific categories (red bar): chronic inflammatory response; positive regulation of NF-kappaB import into nucleus; Overlapping categories (red and blue bar): Inflammatory response; ECM organization; collagen fibril organization; male-specific categories (blue bar) : transforming growth factor beta (TGF-β) receptor signaling pathway, ECM disassembly. Y axis, −log_10_^P-value^; X axis, GO categories of fibrosis-related genes; **D**., Pathway categories of fibrosis-related genes(Top5): Female-specific categories (red bar):’Renin-angiotensin system; Overlapping categories (red and blue bar): NF-kappa B signaling pathway, ECM-receptor interaction, MAPK signaling pathway;Toll-like receptor (TLR) signaling pathway; Male-specific categories (blue bar): TGF-β signaling pathway. Y axis, −log_10_^P-value^; X axis, Pathway categories of fibrosis remodeling related genes. **E**., Gender differences in fibrosis remodeling related genes: Female group fibrosis remodeling related genes: 20 fibrotic remodeling-related genes, with 14 pro-fibrotic gene (up-regulated gene, 8; down-regulated gene 6) and 6 anti-fibrotic gene (up-regulated, 1; down-regulated,5); Male group fibrosis remodeling related genes:16 fibrotic remodeling-related genes, with 15 pro-fibrotic genes (up-regulated, 0; down-regulated, 15) and 1 anti-fibrotic gene (up-regulated, 0; down-regulated, 1) X axis, number of fibrotic remodeling-related genes; Y axis, categories of fibrosis related gene (pro-fibrotic gene and anti-fibrotic gene).red bar, up-regulated pro-fibrotic gene; blue bar, down-regulated pro-fibrotic gene; pink bar, up-regulated anti-fibrotic gene; yellow bar, down- regulated anti-fibrotic gene.

The precise mechanisms and signaling pathways involved in the development of atrial fibrosis in AF remain to be clarified. Three predominant interrelated mechanisms (the renin- angiotensin system, inflammation and oxidative stress) and pathways (TGF-β signaling pathways, WNT signaling pathways, MAPK signaling pathways) appear to be involved and are used as screening principle for categories of AF fibrosis remodeling related genes [6–8]. Thus, the categories of AF fibrosis remodeling related genes were obtained according to the screening principles mentioned above. In our analysis, substantial gender differences could be detected in the categories of AF fibrosis remodeling related genes (Figure 5A and 5B):

- female- specific: GO, 16; Pathway1
- overlapping: GO, 12; Pathway, 6
- male-specific: GO, 15; Pathway0

Representative top5 categories of AF-related gene are shown as follows:

### GO analysis (Figure 5C)

- female-specific group: chronic inflammatory response; positive regulation of NF-kappaB import into nucleus
- overlapping group: Inflammatory response; ECM organization; collagen fibril organization
- male-specific group: transforming growth factor beta (TGF-β) receptor signaling pathway; ECM disassembly

### Pathway analysis (Figure 5D)

- female-specific group: Renin-angiotensin system
- overlapping group: NF-kappa B signaling pathway; ECM-receptor interaction; MAPK signaling pathway; Toll-like receptor (TLR) signaling pathway

male-specific group: TGF-β signaling pathway

Interesting, our analysis showed that in females, the most obvious category was inflammation- related, while in males, the most obvious category was ECM- related (Figure 5C and 5D) underlining the substantial gender-specific differences in regard to fibrosis remodeling related genes in LSP-AF.

### Gender difference in fibrosis remodeling related genes

Of note, the genes obtained from the categories of AF fibrosis remodeling related genes are not all involved in the regulation of fibrosis remodeling [9]. Therefore, in order to clarify the mechanism of gender differences in fibrosis remodeling, in our further analysis we only selected the genes involved in regulation of fibrosis remodeling and the function of each screened gene was carefully looked up on PubMed (www.pubmed.com) (Supplemental Table 12).

Our analysis revealed the following results (Figure 5E):

Female group: 20 fibrotic remodeling-related genes, with 14 pro-fibrotic gene (up-regulated gene, 8; down-regulated gene 6) and 6 anti-fibrotic gene (up-regulated gene, 1; down- regulated, 5).

Male group: 16 fibrotic remodeling-related genes, with 15 pro-fibrotic genes (up-regulated gene, 0; down-regulated gene, 15) and 1 anti-fibrotic gene (up-regulated gene, 0; down- regulated gene, 1).

Interestingly, while both genders were comparable in regard to the number of pro-fibrotic genes (females: 14 and males: 15), the number of up-regulated pro-fibrotic genes was substantially higher in females (*n* = 8), while there were no up-regulated genes in males at all.

### Immunohistochemistry

**Figure 6:**
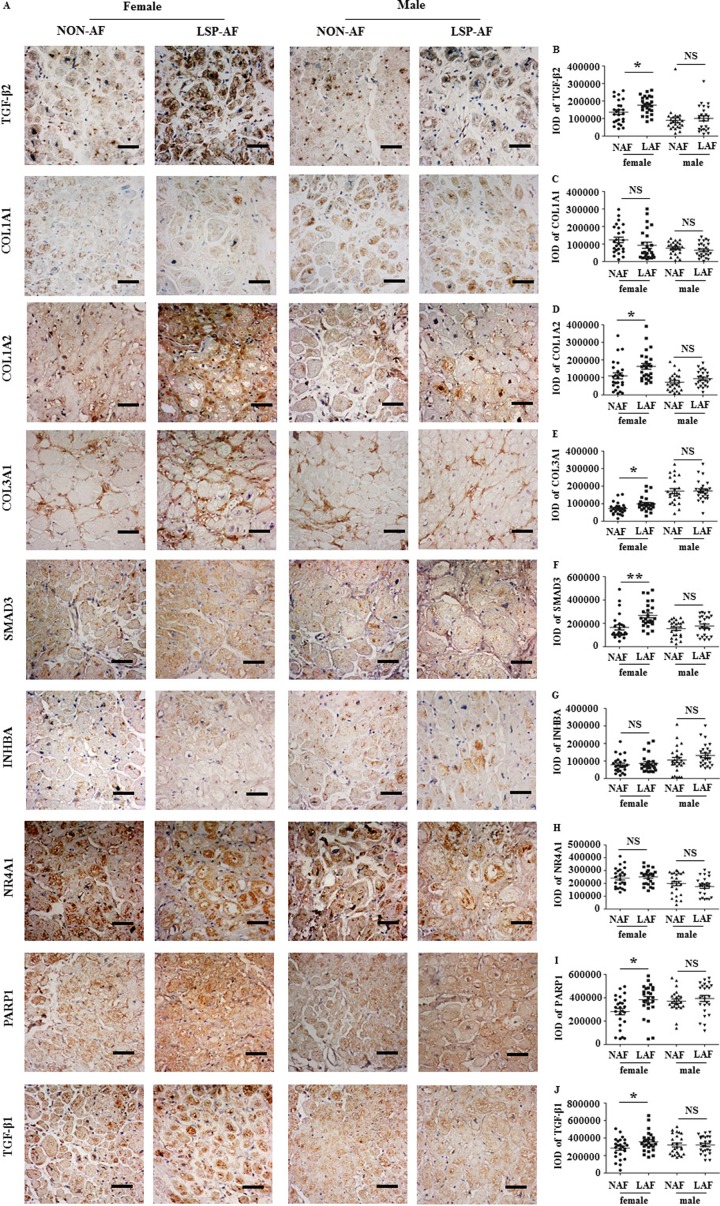
Gender differences in fibrosis remodeling-related proteins (X60, bar = 50 um) **A**., Gender different expression of TGF-β2, COL1A1, COL1A2, COL3A1, SMAD3, INHBA, NR4A1, PARP1, TGF-β1 protein between LSP-AF and NON-AF by immunohistochemistry with hematoxylin counter staining. Quantitative assessments of positive area by integrate optical density (IOD) of TGF-β2, COL1A1, COL1A2, COL3A1, SMAD3, INHBA, NR4A1, PARP1, TGF-β1between LSP-AF and NON-AF respectively are shown **b**.-**J**. Female group: NAF (Dot), *n* = 25; AF (Square), *n* = 25; Male group: NAF (Triangle), *n* = 25; AF (Inverted triangle), *n* = 25. P^NS^ > 0.05 between NAF and LAF; *P* * < 0.05 between NAF and LAF; *P* ** < 0.01 between NAF and LAF.NAF, NON-AF; LAF, long- standing-persistent AF.

In a next step, we further validated our gene expression results from microarray on a protein level. Eight, randomly selected genes (TGF-β2, SMAD3 [10, 11], COL1A1, COL1A2, COL1A3 [12], PARP1 [13], INHBA [14] and NR4A1 [15] from the categories of AF-related genes of the microarray were validated at corresponding protein level by immunohistochemistry (IHC). IHC analysis showed (Figure 6A) that the expression of TGF-β2 (Figure 6B), COL1A2 (Figure 6D), COL3A1 (Figure 6E), SMAD3 (Figure 6F), PARP1 (Figure 6I) were significantly higher in the female LSP-AF group, but not in the male LSP-AF group. In contrast, COL1A1 (Figure 6C), INHBA (Figure 6G), NR4A1 (Figure 6H) did not show significant gender differences in the LSP-AF groups.

Importantly, it is to mention that all significantly higher expressed proteins((TGF-β2, SMAD3 [10, 11], COL1A2, COL1A3 [12], PARP1 [13]) are pro-fibrosis-associated proteins and belong to or regulate the TGF-beta signaling pathway, suggesting that the TGF-β pathway plays a key role in promoting fibrosis remodeling in the female LSP-AF group. To assess this in more detail, in a subsequent step, further analysis for TGF-β1, which is another key molecule in TGF-β signaling, was performed. Notably, it was also found to be significantly higher expressed in the female LSP-AF group, but not in the male LSP-AF group (Figure 6A and 6J) underlining this theory.

### Western blot

**Figure 7:**
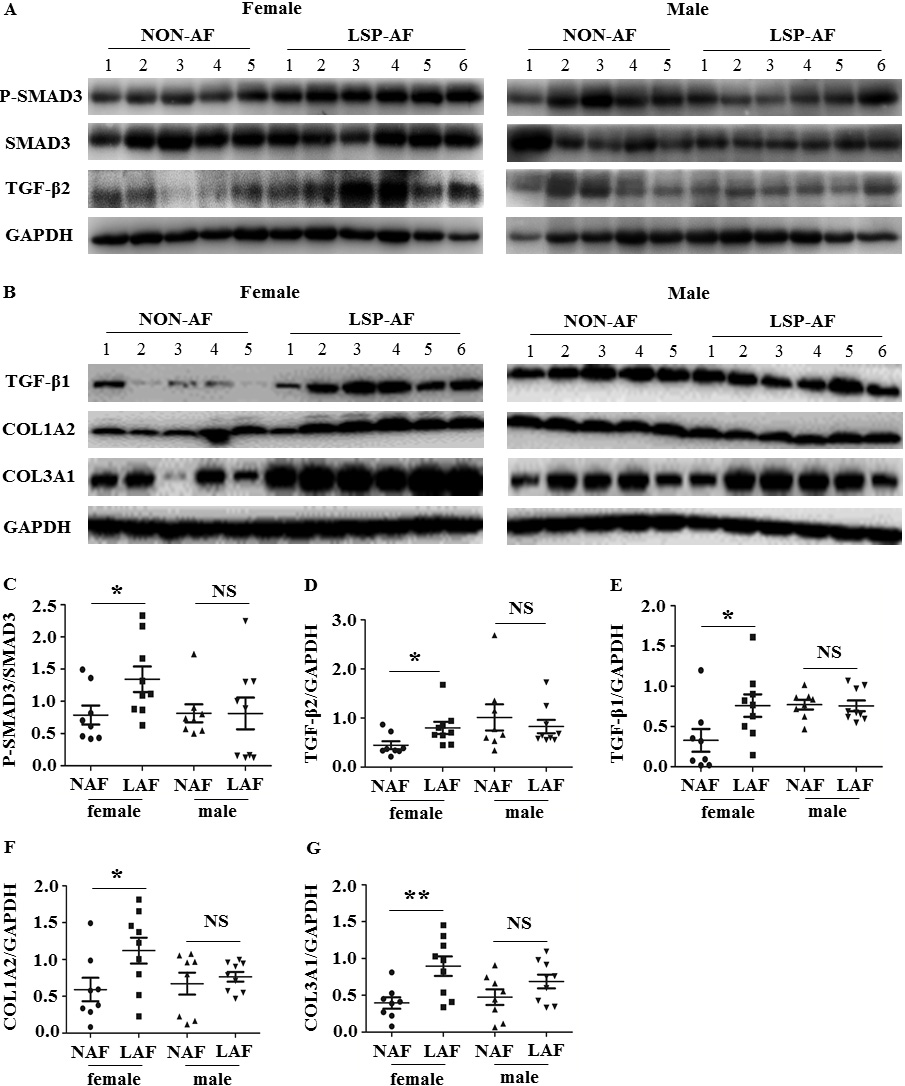
Gender differences in TGF-β signaling pathway **A**. and **B**., Representative images of Western blot results for P-SMAD3, SMAD3, TGF-β2, TGF-β1, COL1A2, COL3A1 (female: non-AF, *n* = 5;LSP-AF, *n* = 6; male: non-AF, *n* = 5;LSP-AF, *n* = 6;) of pulmonary vein sleeves in female / male group with and without AF (GAPDH was used as the loading control). Scatter plots shows a summary and comparison of quantitative results of **C**. P-SMAD3/ SMAD3, **D**. TGF-β2, **E**. TGF-β1, **F**. COL1A2, and (G) COL3A1 were detected between NAF and LAF. Female group: NAF (Dot), *n* = 8; AF (Square), *n* = 9; Male group: NAF (Triangle), *n* = 8; AF (Inverted triangle), *n* = 9.P^NS^ > 0.05 between NAF and LAF; *P* * < 0.05 between NAF and LAF; *P* ** < 0.01 between NAF and LAF. NAF, NON-AF; LAF, long- standing- persistent AF.

Based on our IHC findings and as the TGF-β pathway is suggested the classic signaling pathways in regulation of fibrotic remodeling [11], Western blot analysis was performed. It showed that the expression of P-SMAD3/SMAD3 (Figure 7A and 7C), TGF-β2 (Figure 7A and 7D), TGF-β1 (Figure 7B and 7E), COL1A2 (Figure 7B and 7F), and COL3A1 (Figure 7B and 7G) was significantly up-regulated in the female LSP-AF group, but not in the male LSP-AF group, which further verified the TGF-β signaling pathway to play an important regulatory role in promoting fibrosis remodeling in the female LSP-AF group.

## DISCUSSION

### Significant gender-specific differences in fibrosis remodeling in AF patients

In this study, we detected for the first time significant mechanistic gender differences in the fibrotic remodeling in patients with LSP-AF using a rigorous study design. Notably tissue samples from females with LSP-AF, rather than male, showed a higher degree of fibrosis than samples from patients without AF. Moreover, the degree of fibrosis was closely related to LSP-AF and showed better predictive values for the presence of LSP-AF in females but not in males. Further analysis demonstrated that these gender differences in fibrosis remodeling of LSP-AF were mainly due to inherent differential expression of fibrosis-related genes and proteins. Especially those, related to the TGFβ/Smad3 pathway, were up-regulated in female LSP-AF group suggesting the aggravation of fibrosis remodeling.

### Comparison with other studies and selection of study cohort

Although gender differences in prevalence and outcomes were recently reviewed [16], and despite significant advancements in the clinical understanding of AF in the past decade, to date, only few studies providing mechanistic insight into the gender-specific differences in fibrosis remodeling and AF are available [17, 18]. For instance, in their recent report, Pfannmüller and colleagues biochemically assessed tissue samples from 123 patients with sinus rhythm (SR, *n* = 41) or atrial fibrillation (AF, *n* = 82) and suffering from mitral valve disease, did not detect any significant gender-specific difference in regard to fibrotic remodeling [17]. Interestingly, those findings differ from ours, and we believe that the reasons for this are multifaceted and are mainly attributable to the selection of our patient cohort and our rigorous study design: First, we exclusively included patients with LSP-AF, while previous studies did not specifically do this important distinction between the types of AF. Secondly, even if the previous study had indeed only chosen LSP-AF patients, a biasing effect of potential confounders such as mitral valve disease and accompanying left atrial enlargement were most often not particularly considered and even completely neglected. Mitral valve disease is well established to be often accompanied by left atrial enlargement [19], which is an independent risk factor for fibrosis remodeling and AF [20–22]. Therefore, we only enrolled mitral valve disease patients with LSP-AF that also presented with left atrial enlargement. Finally, in our view, the presence of strict controls was critical to determine our main result that significant gender differences in fibrosis remodeling can be found in patients with LSP-AF. For this reason, we took particular care, that also our non-AF control patients displayed similar characteristics with LSP-AF group in regards to mitral valve disease, and importantly left atrial enlargement, which was not the case in previous [23, 24]. In another report, Cochet and colleagues used cardiac MRI to show that the degree of atrial fibrosis is higher in female AF patients when compared male counterparts [18]. However, while the authors in this study primarily focused on the gender-specific comparison among AF patients, our primary aim was on gender differences in fibrosis remodeling between AF and non-AF patients in order to highlight that the fibrosis plays a gender-specific role in the occurrence and maintenance of AF.

### Aggravation of fibrosis remodeling in women as a reason for the low success rate of AF ablation?

While the exact mechanism for this phenomenon remains to be elucidated, fibrosis has been suggested to play a key role in this regard. Fibrosis remodeling leads to a dissociation in atrial conduction and thereby promotes AF which then ultimately results in a lower efficacy of ablation therapy.

Despite being a reasonable treatment strategy for AF patients in general, catheter ablation outcomes in females are worse when compared to men. [5]. While the reason for this is still unclear, the mechanism and degree of fibrosis remodeling seems to be a key mediator of this gender-specific phenomenon [6] [7]. Our findings clearly demonstrated that female LSP-AF, rather than male, presented with a higher degree of fibrosis than non-AF suggesting that the lower success rates of AF catheter ablation observed in women may be attributed to the aggravation of fibrosis remodeling in females. Thus, the inhibition of fibrosis may increase success rates of AF catheter ablation in females and gender-specific anti-fibrosis treatments may be considered for future therapy concepts in women with LSP-AF.

### Gender-specific molecular mechanisms in atrial fibrosis of AF patients

Although fibrosis may be considered as an interesting therapeutic target in the future, the exact gender-specific underlying molecular mechanisms in atrial fibrosis of LSP-AF are still extensively debated and remain to be elucidated. In this context, our systematic analysis using microarray, immunohistochemistry and Western Blot may provide useful insight. Remarkably, our data indicated that gender differences were not only present in fibrosis remodeling-related genes but also in fibrosis remodeling related proteins. While the microarray results detected 14 pro-fibrotic genes with 8 being up-regulated in women, it showed 15 pro-fibrotic genes, with none being up-regulated in men. In line with these findings, IHC and Western Blot analysis further verified these findings. With numerous genes (that were randomly selected from the microarray analysis; TGF-β2, SMAD3, COL1A2, COL1A3, and PARP1) showing a significant up-regulation the female LSP-AF group (*versus* the female NON-AF group), while no difference was seen between male groups.

Furthermore, the TGF-β pathway is known to be a classic regulatory pathway of fibrosis remodeling [11]. Notably, in our Western Blot analysis, we found several Pro-fibrosis related proteins (TGF-β1, TGF-β2, P-Smad3, COL1A2, and COL1A3), which belong to TGF-β pathway, that showed gender-specific differences between NON-AF and LSP-AF group, with a significant up-regulation in the female LSP-AF group, but not in the male counterpart. Finally, PARP-1, being involved in regulating the TGF-β signaling pathway by promoting TGF-β1-induced Smad3 transactivation and expression of Smad3 target genes, such as collagen Iα1, collagen IIIα1 [13, 25] was also significantly up-regulated females, but not males with LSP-AF.

**Figure 8:**
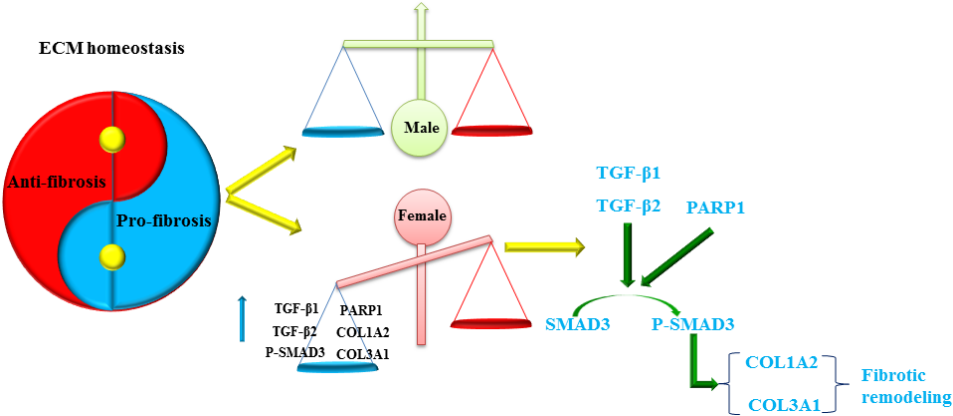
Gender-difference in remodeling and homeostasis of ECM

Taken together, and based on our analysis we hypothesize that in women, but not in men with AF the TGF signaling pathway is activated and thereby promotes the increased expression of fibrosis-related proteins leading to the disturbance of the ECM homeostasis and the aggravation of fibrosis remodeling (Figure 8). Therefore, inhibition of TGFβ/Smad3 pathway-mediated fibrosis could prevent the aggravation of fibrosis remodeling and may improve the success rate of AF catheter ablation in women.

### Limitations

As all patients in our study had LSP-AF, the conclusions drawn from this study cannot be applied to a population with other types of AF such as lonely AF and paroxysmal AF.

## CONCLUSIONS

When compared to men, our study shows a significant aggravation of fibrosis remodeling in women with LSP-AF which may be a major reason for the overall lower success rates of AF catheter ablation in women. As demonstrated by our mechanistic analysis targeting TGFβ/Smad3 pathway-mediated fibrosis could be an interesting focus for future therapy concepts in order to improve the success rate of AF catheter ablation in females.

## MATERIALS AND METHODS

### Study subjects

This study was conducted in the department of Cardiovascular Surgery of Shenyang North Hospital, China.

A total of 166 patients with dilated left atria and concomitant mitral valve disease were enrolled from June 2015 to May 2016 and divided into four groups by gender and AF status (female NON-AF, female LSP-AF, male NON-AF, male LSP-AF) (Supplemental Figure 7).

Since AF when associated with mitral valve disease is often accompanied by left atrial enlargement [21] (which is a well-known independent risk factor for fibrosis remodeling and AF [20]), we only included LSP-AF or NON-AF patients with a similar degree of atrial dilation in order to exclude any experimental bias. The dilated left atria were defined according to ESC guidelines as follows [26]: male left atrial diameter (LAD) > 40mm, female LAD > 38mm. However, since the extent of left atrial enlargement is not defined the similar between genders [26], we decided to choose the left atrial enlargement ratio (left atrial diameter respectively divided by minimum threshold of left atrial enlargement in men and women) as a screening factor (Table 1), in order to eliminate this inconsistency. The similarity of left atrial enlargement rates between male and female patients was an important baseline clinical characteristic for all comparative analyses in our study.

In addition, patients with diabetes and those who reported smoking or alcohol consumption within 10 years were excluded from the study. Patients’ characteristics were listed in the Table 1. The enrolled female patients were all post -menopausal, thus avoiding any potential effect of estrogen. All patients were classified as preoperative functional status III according to New York Heart Association (NYHA) guidelines. The diagnosis of LSP-AF was made according to AHA/ACC/ESC/HRS guidelines [27]. The LSP-AF patients had a history of AF for 1 year after which a rhythm control strategy is often adopted [28]. The NON-AF patients in normal sinus rhythm had no history of any type of AF or use of any anti-arrhythmic drugs.

Trimmed-off tissues of pulmonary vein sleeves (1 cm away from right pulmonary vein orifices) (Supplemental Figure 8) were obtained from the edge of the incisions of left atria during mitral valve surgery +/− MAZE III surgery). In 46 of all patients, the size of each pulmonary vein sleeve was about 8×4mm-6×4mm (female: NON-AF, *n* = 11; LSP-AF, *n* = 12; male: NON-AF, *n* = 11; LSP-AF, *n* = 12).A portion of the tissue (≈5×4mm) was immediately snap-frozen in liquid nitrogen, and stored at −80°C, while the other part (≈3×4mm-1×4mm) was fixed in neutral formalin. In the remaining 120 patients, a piece of pulmonary vein sleeve < 6×4mm (female: NON-AF, *n* = 19; LSP-AF, *n* = 43; male: NON-AF, *n* = 24; LSP-AF, *n* = 34) was fixed in neutral formalin. No complications occurred as a consequence of tissue sampling.

### Ethics approval and informed consent

The Chinese Ethics Committee of Registering Clinical Trials approved this study, and the investigation complied with the principles for the use of human tissues outlined in the Declaration of Helsinki. All patients gave informed consent before participating in the study. Ethics committee approval No: ChiECRCT-20150025. There was no industry involvement in the design or performance of the study or in the analysis of the data.

### Masson trichrome staining

Paraffin-embedded pulmonary vein sleeve tissue samples were sectioned (3 μm) and Masson's trichrome staining (Baso Diagnostics Inc. Zhuhai, China) was performed as described elsewhere to evaluate collagen (blue color) of pulmonary vein sleeve. After Masson's trichrome staining, the collagen volume fraction (CVF) was determined via quantitative morphometry using Image-Pro Plus 6.0 under a 60× microscopic magnification, which was expressed as the percentage of pixels of positive collagen staining divided by total pixels of the image (CVF = collagen area/total area). Five random images with fibrosis of each section were selected. The mean of 5 sections per specimen represented the CVF of the assessed pulmonary vein sleeve.

### Immunohistochemistry

Paraffin-embedded pulmonary vein sleeve tissue samples were sectioned (3 μm), and subjected to de-paraffinization, rehydration, and antigen retrieval before the staining procedures. The tissue slides were blocked with 1% bovine serum albumin for 30 min and then incubated with rabbit anti-human TGF-β1, TGF-β2, SMAD3, INHBA, NR4A1 (1:100, Proteintech, Chicago, USA), COL1A1, COL1A2, COL3A1 (1:100, Santa Cruz Biotechnology Inc. CA, USA), and PARP1 (1:100, CST, Boston, USA) overnight at 4°C. Then, antibody binding was detected using Horseradish peroxidase-conjugated goat anti- rabbit IgG (1:500, Zhongshan Golden Bridge Biotechnology Co., Beijing, China) for 1 h at 37°C and then rinsed with water, counterstained with hematoxylin and mounted with Neutral balsam. The sections were visualized with diaminobenzidine solution, lightly counterstained with hematoxylin, and observed using light microscopy. Negative control slides were processed without primary antibody and were included for each staining. All slides were reviewed by 2 pathologists. The presence of buffy or brown diaminobenzidine precipitates is indicative of positive reactivity. The integral optical density (IOD) of immunohistochemical intensity was analyzed by Image-Pro Plus 6.0 software. Each value represents IOD counted at a high-power view (x60) by microscopy. The mean value represents the average number derived from five high-power fields of each case.

### RNA extraction

To extract RNA, frozen tissues were ground into powder with mortar and pestle and resuspended in TRIzol reagent (Invitrogen, Carlsbad, CA, USA). RNA purification was performed on the RNA-containing aqueous phase with the RNeasy minikit (Qiagen, Valencia, CA, and USA). RNA was then eluted with RNase-free water and treated with turbo DNase (Ambion) to remove any contaminating DNA. Quantification and RNA quality evaluation were performed with Nanodrop and Agilent2100 Bioanalyzer, respectively. A260/A280 in all samples was more than 1.8 and there were no degradation fragments in electrophoresis images (data not shown).

### Microarray analysis

Purified RNA was amplified and transcribed into cRNA utilizing a random priming method and cDNA was labeled and hybridized to the Gene Chip Human Gene 2.0 ST Array (Affymetrix). In total, 70,524 transcripts both coding and non-coding were covered. Gene Chip Human Gene 2.0 ST Array protocol was followed; in brief: Step 1, preparation of the RNA sample, kit and reagents (TRIzol reagent and miRNeasy Mini Kit). Step 2, total RNA clean-up and RNA QC. Step 3, preparation of labeling reaction and reagents: Gene Chip WT Terminal Labeling and Controls Kit. Step 4, purification of the labeled/amplified RNA and labeled cRNA QC. Step 5, hybridization. Step 6, microarray wash. Step 7, scanning. Step 8; data extraction using Affymetrix Extraction Software. The arrays were scanned by Gene Chip® Command Console® Software (AGCC) and the acquired array images were analyzed by Affymetrix Gene Chip Operating Software. QC analysis of Gene 2.0 ST Array data was performed using the Affymetrix® Expression Console™ Software. Differentially expressed genes were selected with the Two Class Dif method, and were identified through random variance model (RVM) [29]. Heat maps representing differentially regulated genes were generated using Cluster 3.0. The threshold used to screen up- or down-regulated genes was fold-change > 1.2 and *P* < 0.05. The microarray data discussed in this study have been deposited in NCBI's Gene Expression Omnibus and are accessible through GEO Series accession number GSE76899 (http://www.ncbi.nlm.nih.gov/geo/query/acc.cgi? acc = GSE76899).

### Gene ontology and pathway analysis

Gene ontology (GO) and Pathway Analysis were applied to classify differentially expressed AF related genes. GO is the key functional classification method used at NCBI and can organize genes into hierarchical categories and uncover gene regulatory networks on the basis of biological processes and molecular functions. Canonical pathways representing genes were identified using the curated IPA (Ingenuity Pathway Analysis) database according to KEGG, Biocarta, and Reatome, as previously described. Fisher's exact test and v2 test were used for statistical analysis. The threshold of significance was defined by the *P*-value and FDR, and enrichment score was expressed in −log_10_^P-value^ for comparative analysis of the categories [30–32].

### Western blotting

Tissue samples (≈22 mg) were thawed, minced and homogenized in lysis buffer with a Dounce homogenizer on ice, and centrifuged at 12000 rpm for 20 minutes at 4°C. Supernatants were collected, and the protein concentrations were measured with Pierce^®^ BCA Protein Assay Kit (Thermo Scientific, Rockford, IL, USA). Equal amounts of proteins were separated on SDS gels and electro blotted onto polyvinylidene difluoride (PVDF) membrane (EMD Millipore, Billerica, USA). Membranes were incubated overnight with the following antibodies: rabbit anti-human TGF-β1, TGF-β2, SMAD3 (1:1000, Proteintech, Chicago, USA), P-SMAD3 (1:1000, CST, Boston, USA), COL1A2, COL3A1 (1:1000, Santa Cruz Biotechnology Inc. CA, USA). Horseradish peroxidase-conjugated goat anti- rabbit IgG (1:500, Zhongshan Golden Bridge Biotechnology Co., Beijing, China) were used as secondary antibodies. Rabbit anti-human GAPDH (1:5000, Abcam, Cambridge, UK) was used in every experiment for the internal control. Reactive bands were developed and enhanced by SuperSignal West Pico chemiluminescence detection reagents according to the instructions of the manufacturer (Thermo Scientific, Rockford, IL, USA), and the images were scanned, and densities of each band were analyzed by Total Lab software (Nonlinear Dynamics). The results are presented as percent change compared with that in controls after normalization to the GAPDH bands of each sample.

### Statistical analysis

All group data were expressed as the mean ± SEM from at least three independent experiments. The chi-square test was used to determine differences of constituent ratio between groups. Student's unpaired t-test was used to determine differences between groups. All P-values were two-sided and obtained by using PASW Statistics 18 and Graph Pad Prism 5. Statistical significance was considered at *P* < 0.05.

## SUPPLEMENTARY MATERIALS FIGURES AND TABLES




