# Supplementary material for: Gender differences in fibrosis remodeling in patients with long-standing persistent atrial fibrillation
Source: Oncotarget. 2017 Mar 17;8(32):53714–29. doi: 10.18632/oncotarget.16342 (PMC5581144; doi:10.18632/oncotarget.16342)
Supplement: Supplementary file 1 [file oncotarget-08-53714-s001.pdf]

Gender differences in fibrosis remodeling in patients with long-standing persistent atrial fibrillation

Supplementary Material

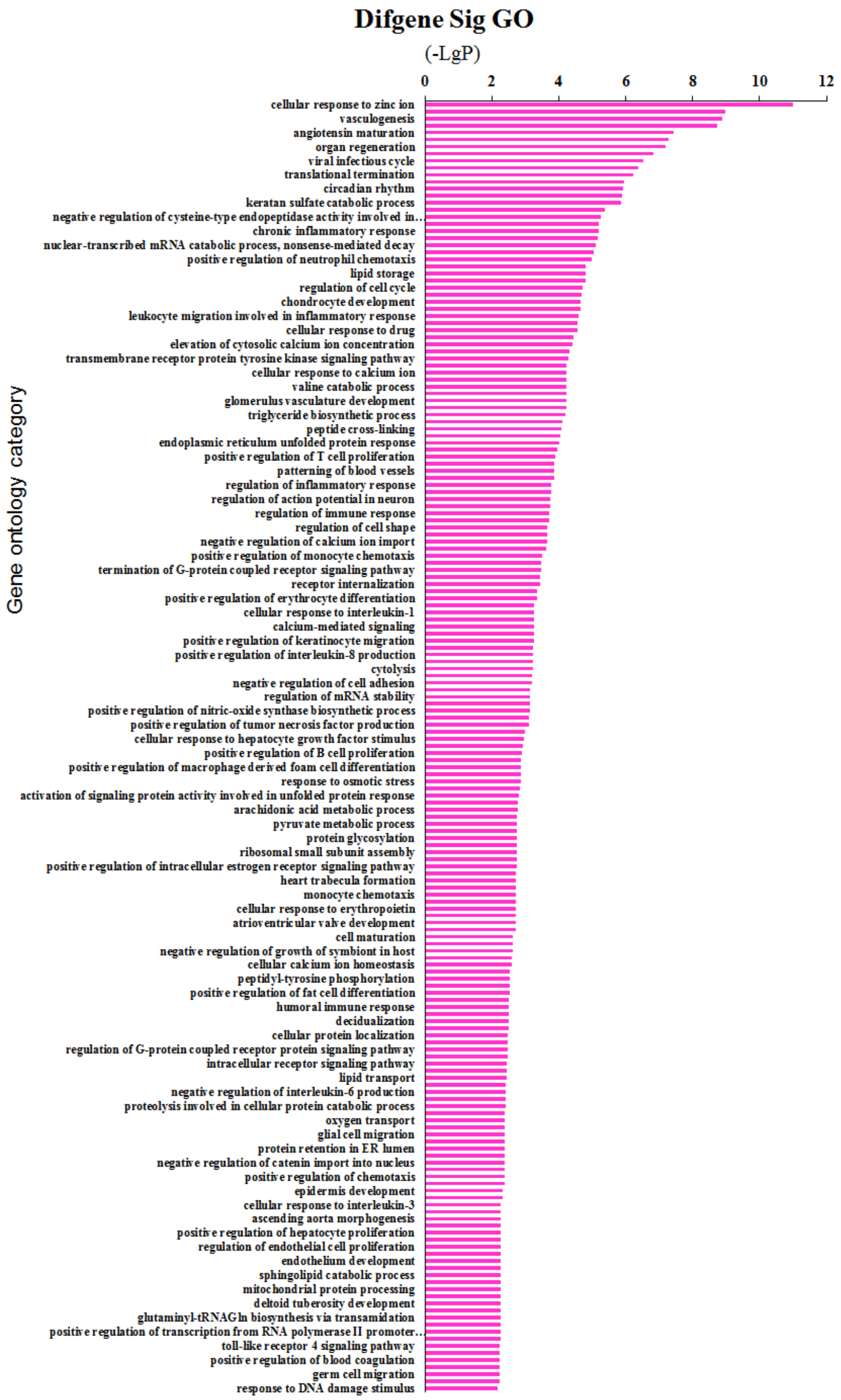

Supplementary Figure 1: GO analysis: female-specific categories of AF-related gene.

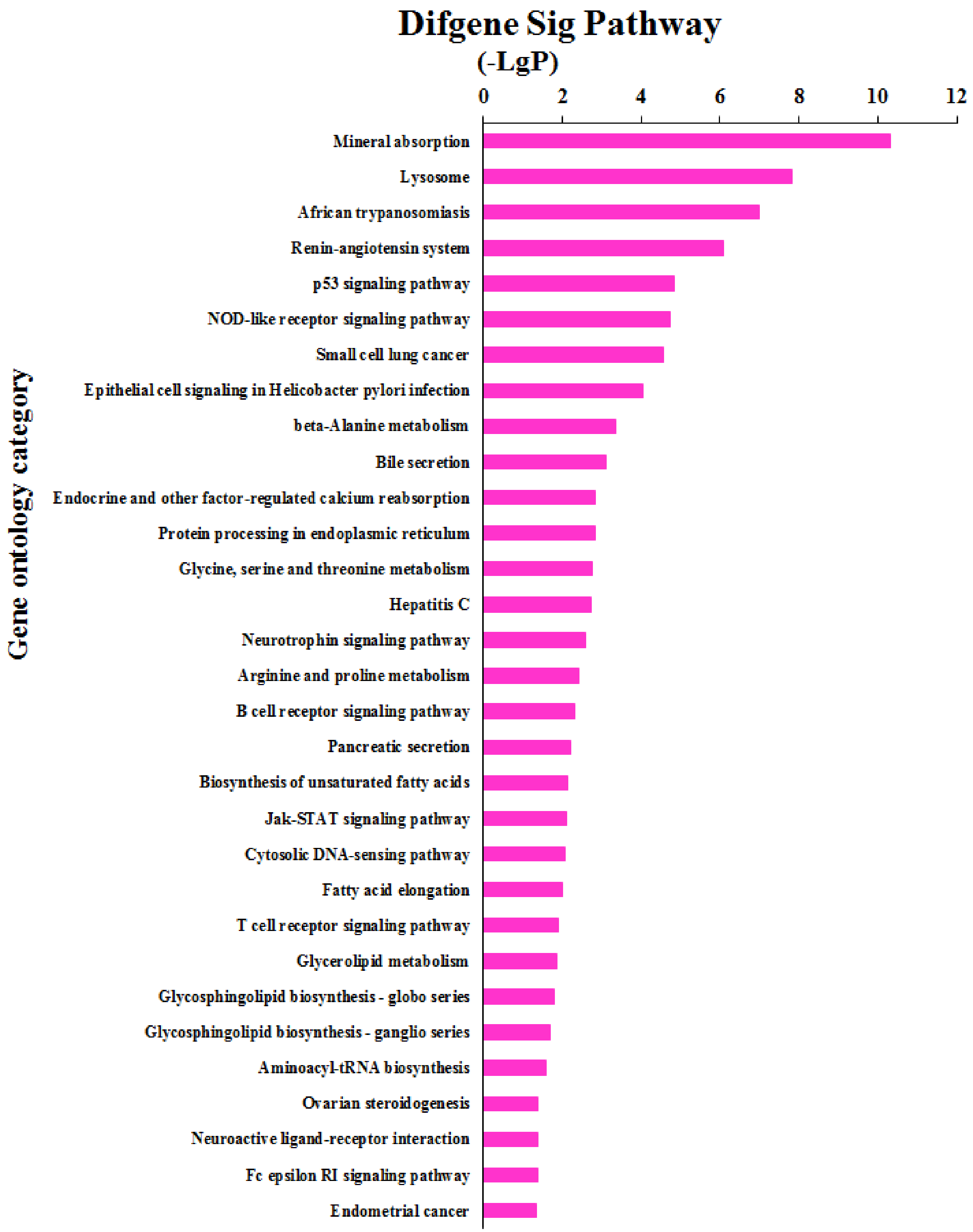

Supplementary Figure 2: Pathway analysis: female-specific categories of AF-related gene.

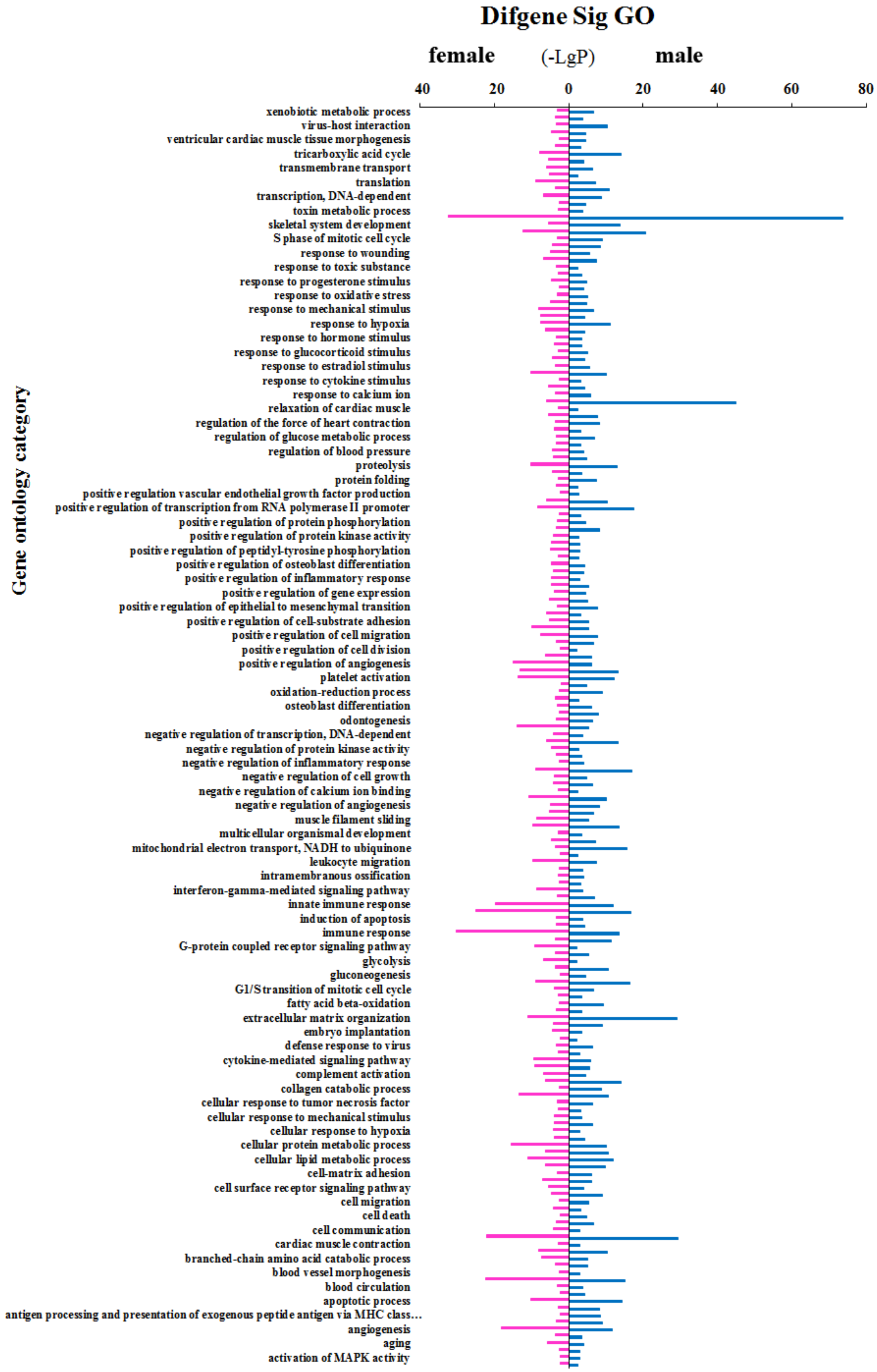

Supplementary Figure 3: GO analysis: overlapping categories of AF-related gene. female, pink bar; male, blue bar.

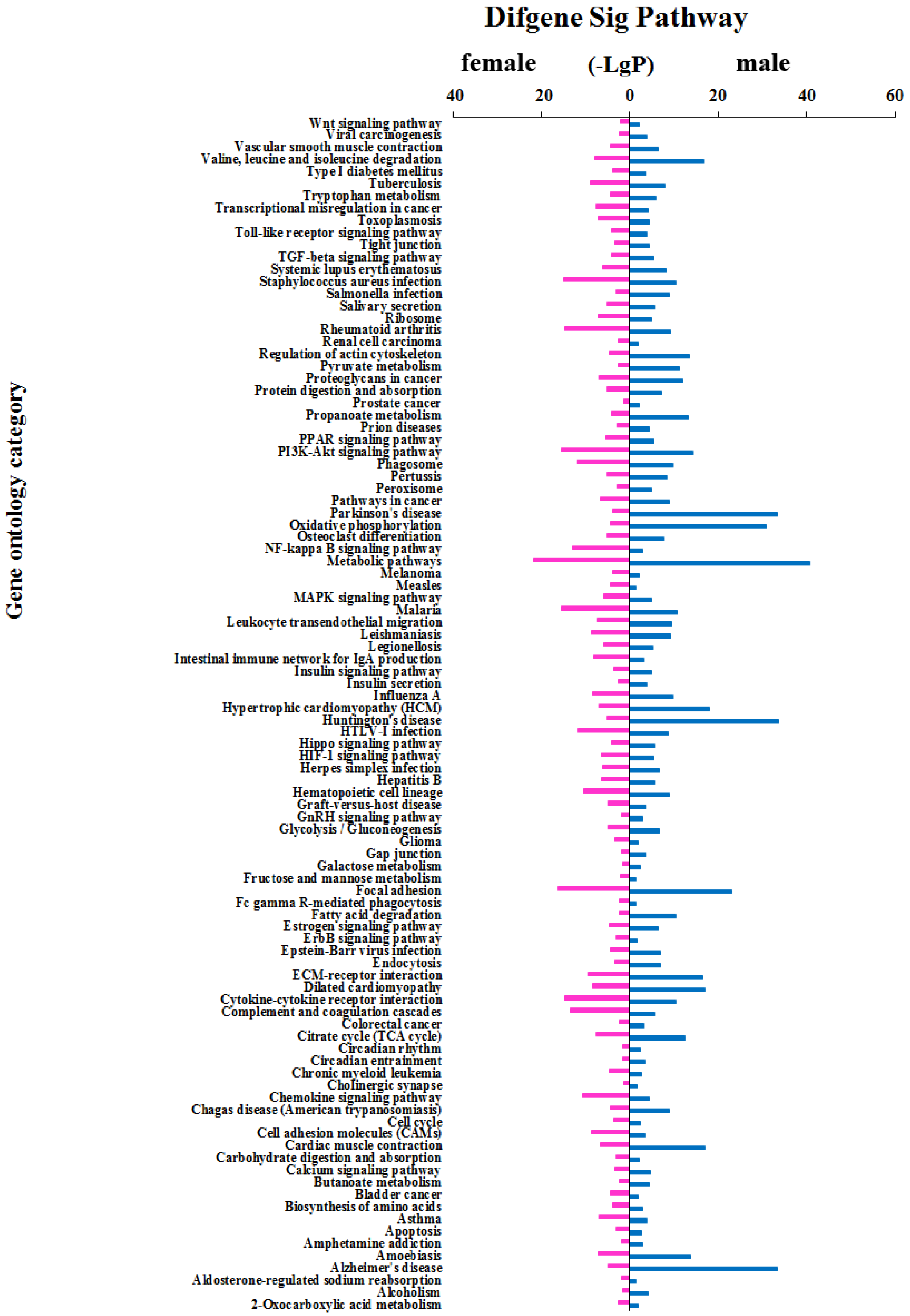

Supplementary Figure 4: Pathway analysis: overlapping categories of AF-related gene. female, pink bar; male, blue bar.

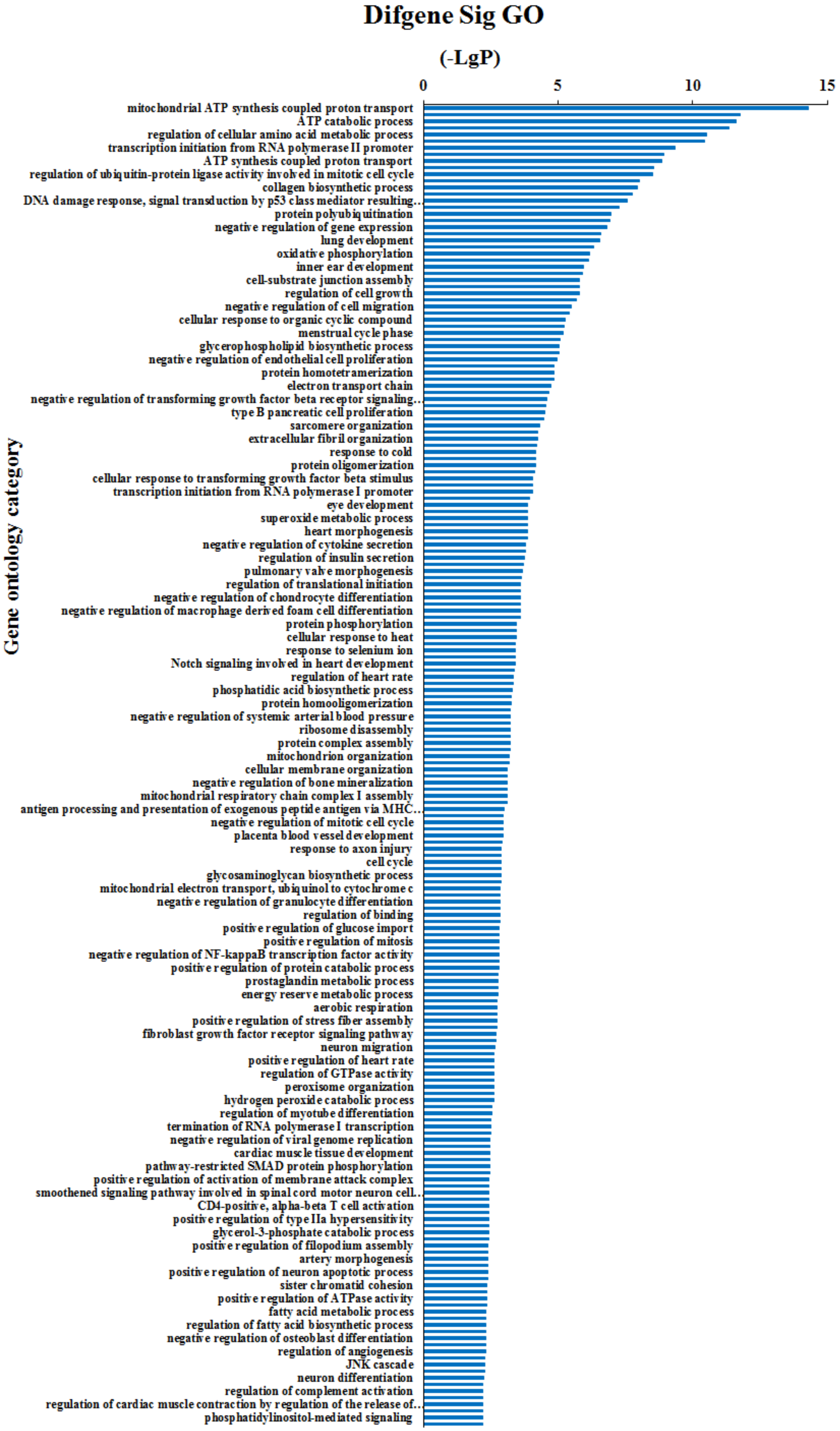

Supplementary Figure 5: Pathway analysis: female-specific categories of AF-related gene.

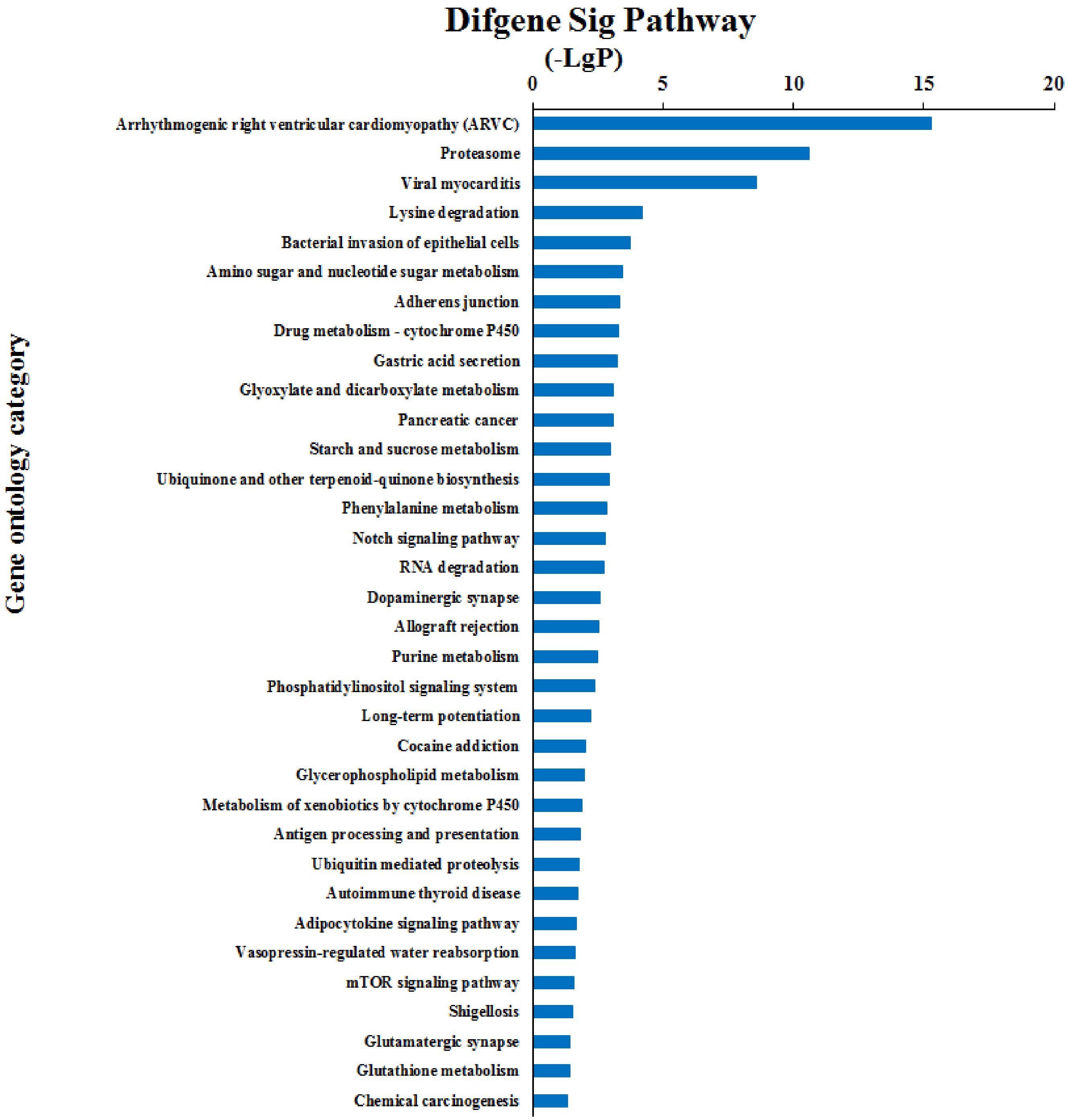

Supplementary Figure 6: Pathway analysis: male-specific categories of AF-related gene.

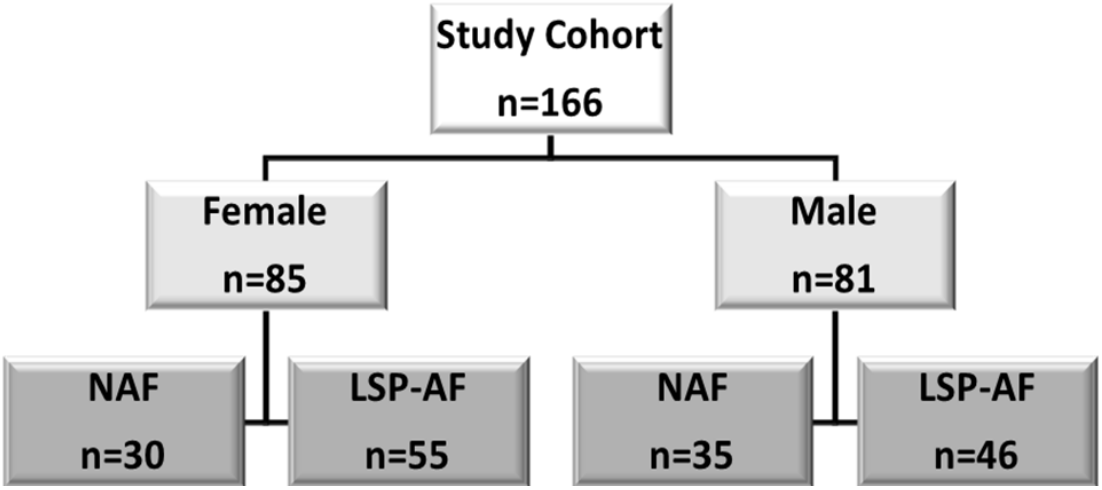

Supplementary Figure 7: Group of selected patients.

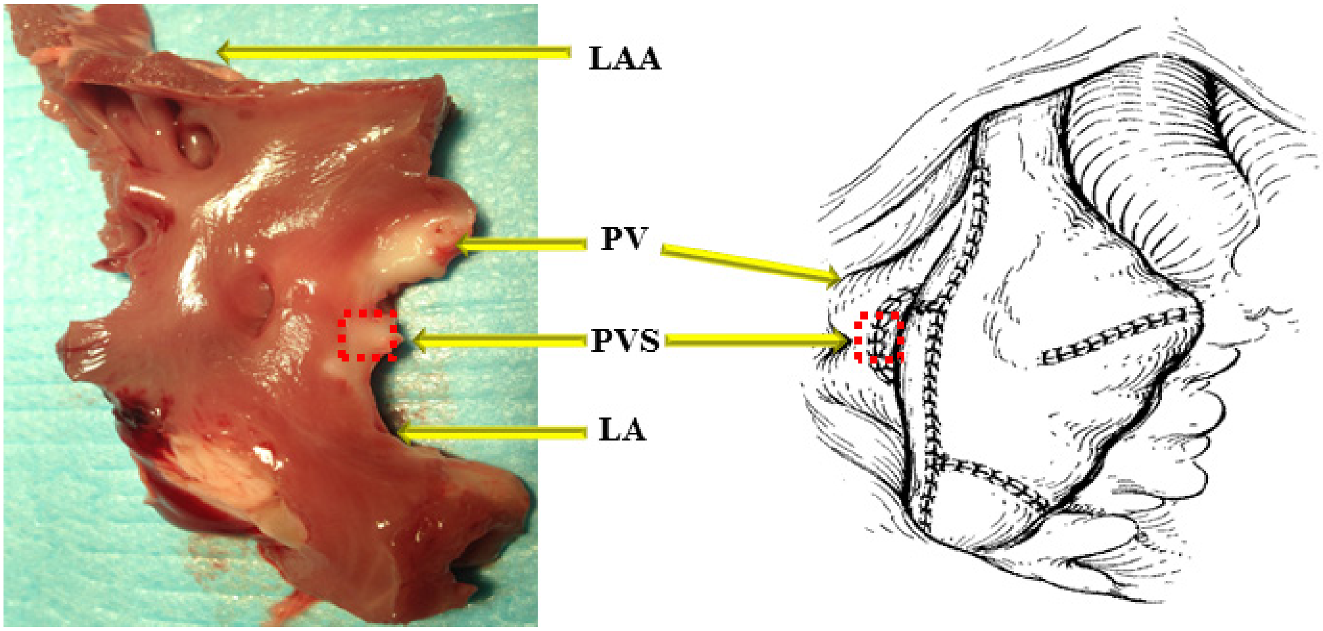

Supplementary Figure 8: Schematic drawing of PVS. LAA, left atrial appendage; LA, left atrium; PV, pulmonary vein; PVS, pulmonary vein sleeve (red dotted line box).
