# Supplementary material for: Gender differences in fibrosis remodeling in patients with long-standing persistent atrial fibrillation
Source: Oncotarget. 2017 Mar 17;8(32):53714–29. doi: 10.18632/oncotarget.16342 (PMC5581144; doi:10.18632/oncotarget.16342)
Supplement: Supplementary file 2 [file oncotarget-08-53714-s002.doc]

**Supplementary Table 1** Comparative analysis of Clinical Characteristics between female and male

| **Classification** | **Female**  **(n=85)** | **Male**  **(n=81)** | **P-value** |
| --- | --- | --- | --- |
| **Age, y** | 58.1±0.6 | 57.9±0.6 | 0.88 |
| **Clinical diagnosis（n%）** |  |  |  |
| MS+MR | 54(63.5%) | 20(24.7%) | < 0.0001 |
| MR | 29(34.1%) | 59(72.8%) | < 0.0001 |
| MS | 2(2.4%) | 2(2.5%) | 1 |
| **Accompanied disease (n%）** |  |  |  |
| AVD | 29(34.1%) | 28(34.6%) | 1 |
| TVD | 22(25.9%) | 25(30.9%) | 0.48 |
| CHF | 2(2.4%) | 0 (0%) | 0.16 |
| CHD | 28(3.29%) | 23(28.4%) | 0.53 |
| Cerebral infarction | 12(14.1%) | 3(3.7%) | 0.02 |
| Left atrial thrombus | 6(7.1%) | 12(14.8%) | 0.11 |
| Pulmonary hypertension | 8(9.4%) | 18(22.2%) | 0.02 |
| Hyperthyroidism | 2(2.4%) | 2(2.5%) | 1 |
| Gout | 0(0%) | 1(1.2%) | 0.30 |
| Hypertension |  |  |  |
| I | 1(1.2%) | 1(1.2%) | 1 |
| II | 2(2.4%) | 6(7.4%) | 0.13 |
| III | 16(18.8%) | 12(14.8%) | 0.49 |
| **Echocardiography** |  |  |  |
| LAD(mm) | 48.3±0.6 | 50.4±0.6 | < 0.05 |
| LAER | 1.3±0.02 | 1.3±0.01 | 0.62 |
| LVEF | 0.58±0.008 | 0.56±0.006 | 0.16 |
| LVFS | 0.29±0.005 | 0.28±0.005 | 0.08 |
| LVEDD(mm) | 50.8±0.6 | 55.2±0.7 | < 0.0001 |

Data is presented as means±SEM. LSP-AF, long standing persistent atrial fibrillation; MS, mitral stenosis; MR, mitral regurgitation; AVD, Aortic valve disease; TVD, tricuspid valve disease; CHF, congestive heart failure ;CHD, coronary heart disease; LAD, left atrial diameter; LAER, left atrial enlargement ratio: female, LAD/38;male, LAD/40; LVEF, left ventricular ejection fraction; LVFS, left ventricular fractional shortening; LVEDD, left ventricular end diastolic dimension; P-value（female vs male）.

**Supplementary Tab2 Correlation between LSP-AF and fibrosis in male and female**

| **Correlation** | **female** | **male** |
| --- | --- | --- |
| Number of XY Pairs | 85 | 81 |
| NAF | 30 | 35 |
| LSP-AF | 55 | 46 |
| Pearson r | 0.3319 | 0.04050 |
| 95% confidence interval | 0.1277 to 0.5090 | -0.1795 to 0.2566 |
| P value (two-tailed) | 0.0019 | 0.7196 |
| P value summary | ** | ns |
| Is the correlation significant? (alpha=0.05) | Yes | No |
| R squared | 0.1101 | 0.001640 |

LSP-AF, long standing persistent atrial fibrillation

**Supplemental Tab3.** Female dysregulated mRNA of PVS from patients with LSP-AF

(Total mRNA: n=516; upregulated mRNA, n=459; downregulated mRNA, n=57)

| **Gene**  **Symbol** | **Accession Number** | **Gene Feature** | **Gene Symbol** | **Accession**  **Number** | **Gene Feature** |
| --- | --- | --- | --- | --- | --- |
| SMYD2 | NM_020197 | up | HBA2 | ENST00000534957 | down |
| ATP1B4 | NM_001142447 | up | HBA1 | NM_000558 | down |
| MYL3 | NM_000258 | up | CH25H | NM_003956 | down |
| NPR3 | NM_000908 | up | MT2A | NM_005953 | down |
| CXCL12 | NM_000609 | up | NR4A1 | NM_001202233 | down |
| KCNIP2 | NM_014591 | up | ALAS2 | NM_000032 | down |
| CES2 | NM_003869 | up | ATF3 | NM_001030287 | down |
| SLN | NM_003063 | up | RNF122 | NM_024787 | down |
| ID1 | NM_002165 | up | OR1A1 | ENST00000304094 | down |
| MRPS14 | NM_022100 | up | C19orf71 | NM_001135580 | down |
| NRP1 | NM_001024628 | up | CEACAM8 | NM_001816 | down |
| FHL2 | BC014397 | up | RIPPLY3 | NM_018962 | down |
| VWF | NM_000552 | up | KLF2 | NM_016270 | down |
| ECHS1 | NM_004092 | up | HES1 | NM_005524 | down |
| APLNR | NM_005161 | up | B3GNT5 | NM_032047 | down |
| HIST1H4H | NM_003543 | up | RAI2 | NM_001172732 | down |
| ZNF224 | NM_013398 | up | ZFP36 | NM_003407 | down |
| FBXL4 | NM_012160 | up | JUNB | BC009465 | down |
| EHHADH | NM_001966 | up | GADD45B | NM_015675 | down |
| NUAK1 | NM_014840 | up | CXCL2 | NM_002089 | down |
| SPOP | NM_001007226 | up | CDKN1A | NM_000389 | down |
| ZNF223 | NM_013361 | up | PER1 | NM_002616 | down |
| CD93 | NM_012072 | up | PF4 | NM_002619 | down |
| SLC25A6 | NM_001636 | up | MIDN | NM_177401 | down |
| MALL | NM_005434 | up | CXCR3 | NM_001504 | down |
| NUDT21 | NM_007006 | up | IL8 | NM_000584 | down |
| IGFBP3 | NM_000598 | up | SOCS3 | NM_003955 | down |
| FREM1 | NM_144966 | up | RHOB | NM_004040 | down |
| ZNF546 | NM_178544 | up | HEMGN | NM_018437 | down |
| SSR3 | NM_007107 | up | GGN | NM_152657 | down |
| ATP6V0E1 | NM_003945 | up | PDZK1IP1 | NM_005764 | down |
| RTCB | NM_014306 | up | MT1A | NM_005946 | down |
| MED28 | NM_025205 | up | HBB | NM_000518 | down |
| SLC25A20 | NM_000387 | up | THBS1 | NM_003246 | down |
| ITM2C | NM_001012514 | up |  | ENST00000531638 | down |
| RAB30 | NM_014488 | up | EGR1 | NM_001964 | down |
| RNF141 | NM_016422 | up | PPBP | NM_002704 | down |
| NAGA | NM_000262 | up | BTG2 | NM_006763 | down |
| MRFAP1L1 | NM_203462 | up | AREG | NM_001657 | down |
| ZNF284 | NM_001037813 | up | SIK1 | NM_173354 | down |
| GIMAP6 | NM_001244071 | up | ADAMTS4 | NM_005099 | down |
| UTP3 | NM_020368 | up | IGLJ4 | ENST00000390326 | down |
| PHB2 | NM_001144831 | up | PLAUR | NM_001005376 | down |
| PAMR1 | NM_001001991 | up | JUND | NM_005354 | down |
| PIP4K2B | NM_003559 | up | FOSL2 | NM_005253 | down |
| ARMC1 | NM_018120 | up | EFCAB8 | OTTHUMT00000332145 | down |
| CARHSP1 | NM_014316 | up | TIGD5 | ENST00000504548 | down |
| MAGT1 | NM_032121 | up | SCXA | NM_001008271 | down |
| GUCA2B | NM_007102 | up | TNFAIP8L3 | NM_207381 | down |
| HNRNPUL2 | NM_001079559 | up | SLITRK1 | NM_052910 | down |
| YIPF5 | NM_001024947 | up | FOSB | NM_001114171 | down |
| DDX23 | NM_004818 | up | NR4A2 | NM_006186 | down |
| PRPF4 | NM_001244926 | up | FOS | NM_005252 | down |
| LOC100126582 | BC108270 | up | RGS1 | NM_002922 | down |
| AK4 | NM_001005353 | up | MT1M | NM_176870 | down |
| SLC33A1 | NM_001190992 | up | MT1X | NM_005952 | down |
| FAM198B | NM_001031700 | up | MT1G | NM_005950 | down |
| CALM3 | NM_005184 | up | IL6 | NM_000600 | down |
| CHURC1 | NM_001204063 | up | NR4A3 | NM_173199 | down |
| ZNF658B | BC137089 | up | RGS2 | NM_002923 | down |
| NUP43 | NM_198887 | up | NFKBIZ | NM_001005474 | down |
| HNRNPA1L2 | NM_001011724 | up | C5AR1 | NM_001736 | down |
| TAF15 | NM_139215 | up | DEFA1B | NM_001042500 | down |
| SNX12 | NM_001256185 | up | MT1F | NM_005949 | down |
| HEXA | NM_000520 | up | TNFAIP3 | NM_006290 | down |
| JAM2 | NM_021219 | up | CLEC4E | NM_014358 | down |
| MED1 | NM_004774 | up | TREM1 | NM_001242589 | down |
| METTL10 | NM_212554 | up | MT1H | NM_005951 | down |
| ABCG2 | NM_004827 | up | PRUNE2 | NM_015225 | down |
| SRP9 | NM_001130440 | up | MT1E | NM_175617 | down |
| PI4K2A | NM_018425 | up | NFIL3 | NM_005384 | down |
| CCDC121 | NM_001142683 | up | IL1B | NM_000576 | down |
| NIF3L1 | NM_001136039 | up | ADAMTS1 | NM_006988 | down |
| CNEP1R1 | NM_153261 | up | FPR1 | NM_001193306 | down |
| EXT2 | NM_000401 | up | DUSP1 | NM_004417 | down |
| PGGT1B | NM_005023 | up | CDR1 | NM_004065 | down |
| SMDT1 | NM_033318 | up | JUN | NM_002228 | down |
| PCDHB3 | NM_018937 | up | PRELP | NM_002725 | down |
| KIAA0100 | NM_014680 | up | LOC100127983 | NM_001190972 | down |
| MLEC | NM_014730 | up | MT1B | NM_005947 | down |
| RPL8 | NM_033301 | up | CCNH | NM_001199189 | down |
| HCN1 | NM_021072 | up | AFF2 | NM_001170628 | down |
| B3GALT2 | NM_003783 | up | CSRNP1 | NM_033027 | down |
| SVEP1 | NM_153366 | up |  | ENST00000454671 | down |
| KDR | NM_002253 | up | NLRP3 | NM_001079821 | down |
| QRSL1 | NM_018292 | up | MYC | NM_002467 | down |
| ARMCX1 | NM_016608 | up | KLF4 | NM_004235 | down |
| BNIP3 | NM_004052 | up | ACTA2 | NM_001141945 | down |
| MOXD1 | NM_015529 | up | XG | NM_001141919 | down |
| PRKCH | NM_006255 | up | SERPINE1 | NM_000602 | down |
| ATRNL1 | NM_207303 | up | CD177 | NM_020406 | down |
| CEP192 | NM_032142 | up | CD177P1 | ENST00000378007 | down |
| MRPL33 | NM_004891 | up | OLR1 | NM_001172632 | down |
| ZNF436 | NM_001077195 | up | C1orf138 | BC132992 | down |
| DHTKD1 | NM_018706 | up | CLDN1 | NM_021101 | down |
| M6PR | NM_001207024 | up | APOBEC3A | NM_145699 | down |
| AKAP10 | NM_007202 | up | MAT2A | NM_005911 | down |
| PCYOX1 | NM_016297 | up | SPRR1B | NM_003125 | down |
| ZBTB6 | NM_006626 | up | SHC4 | NM_203349 | down |
| PSMB3 | NM_002795 | up | CEBPD | NM_005195 | down |
| WDR5B | NM_019069 | up | TSPYL2 | NM_022117 | down |
| TXNDC11 | NM_015914 | up | BHLHE40 | NM_003670 | down |
| LBH | NM_030915 | up | ARG1 | NM_000045 | down |
| ACLY | NM_001096 | up | RGS16 | NM_002928 | down |
| PGAM4 | NM_001029891 | up | SPANXC | NM_022661 | down |
| EEF1G | NM_001404 | up | MAFF | NM_001161572 | down |
| TNPO3 | NM_001191028 | up | TRAJ14 | ENST00000390523 | down |
| ABHD10 | NM_018394 | up | IER2 | NM_004907 | down |
| DPH5 | NM_001077394 | up | PPP1R14A | NM_001243947 | down |
| ZNF426 | NM_024106 | up | TMEM75 | BC137383 | down |
| TMEM203 | BC009461 | up | WFDC1 | NM_021197 | down |
| PSAP | NM_001042465 | up | HEYL | NM_014571 | down |
| GGA2 | NM_015044 | up | OR1K1 | NM_080859 | down |
| AASDHPPT | NM_015423 | up | TIPARP | NM_001184717 | down |
| JAM3 | NM_001205329 | up | ZNF831 | NM_178457 | down |
| ZNF75A | NM_153028 | up | CA1 | NM_001164830 | down |
| AHCYL1 | NM_001242673 | up |  | ENST00000381466 | down |
| AP3M1 | NM_012095 | up | IFRD1 | NM_001007245 | down |
| ZIK1 | NM_001010879 | up | ADAMTS8 | NM_007037 | down |
| PARP1 | NM_001618 | up | NAP1L3 | NM_004538 | down |
| METTL3 | NM_019852 | up | P2RX1 | NM_002558 | down |
| SLC2A12 | NM_145176 | up | EFHC2 | NM_025184 | down |
| UGT2B4 | NM_021139 | up | HBZ | NM_005332 | down |
| CMA1 | BC069370 | up | MEGF9 | NM_001080497 | down |
| ANGPTL2 | NM_012098 | up | C10orf10 | NM_007021 | down |
| TM4SF18 | NM_138786 | up | OR4S1 | NM_001004725 | down |
| PDK1 | NM_002610 | up | PADI4 | NM_012387 | down |
| CYP1B1 | NM_000104 | up | NEDD9 | NM_006403 | down |
| FOLR2 | NM_000803 | up | FOXQ1 | NM_033260 | down |
| TXNDC5 | NM_001145549 | up | KBTBD13 | NM_001101362 | down |
| RPS15 | NM_001018 | up | CTRB1 | NM_001906 | down |
| LRRC6 | NM_012472 | up | IGLJ7 | ENST00000390330 | down |
| ARMCX3 | NM_177948 | up | CAMP | NM_004345 | down |
| EBF2 | NM_022659 | up | KRTAP1-1 | ENST00000543328 | down |
| ALDH1A2 | NM_001206897 | up | GDF2 | NM_016204 | down |
| SPR | NM_003124 | up | ZC3H12A | NM_025079 | down |
| KRTAP19-4 | NM_181610 | up | KCNMB1 | NM_004137 | down |
| COL6A6 | NM_001102608 | up | ASAP2 | NM_001135191 | down |
| ZNF146 | NM_001099638 | up | OXER1 | NM_148962 | down |
| EBF3 | NM_001005463 | up | FKBPL | NM_022110 | down |
| PXMP2 | NM_018663 | up | TMEM86B | NM_173804 | down |
| SDHD | NM_003002 | up | C1QL4 | NM_001008223 | down |
| VPS35 | NM_018206 | up | OR52B6 | NM_001005162 | down |
| EEF2 | NM_001961 | up | USP17L1P | OTTHUMT00000318957 | down |
| ZNF552 | NM_024762 | up | AXIN2 | NM_004655 | down |
| SCO1 | NM_004589 | up | KLHDC7B | NM_138433 | down |
| WDR3 | NM_006784 | up | CDCA5 | ENST00000438878 | down |
| MECOM | NM_001105077 | up | OR6C1 | NM_001005182 | down |
| LCLAT1 | NM_182551 | up | OR2L3 | NM_001004687 | down |
| IMPAD1 | NM_017813 | up | VASP | NM_003370 | down |
| MUL1 | NM_024544 | up | APOF | NM_001638 | down |
| ZFP14 | NM_020917 | up | SFN | NM_006142 | down |
| NOP9 | NM_174913 | up | C10orf25 | NM_001039380 | down |
| PREB | NM_013388 | up | DEGS2 | NM_206918 | down |
| RNF185 | NM_001135825 | up | TMEM240 | NM_001114748 | down |
| MTX3 | NM_001010891 | up | PLK3 | NM_004073 | down |
| RRAGC | NM_022157 | up | C9orf106 | NM_001012715 | down |
| C6orf106 | NM_022758 | up | FATE1 | NM_033085 | down |
| SLC4A4 | NM_003759 | up | SPATC1 | NM_001134374 | down |
| KLHL36 | NM_024731 | up | TSSK3 | NM_052841 | down |
| SLC5A1 | NM_000343 | up | THBD | NM_000361 | down |
| ENPP2 | NM_001040092 | up | EXPH5 | NM_015065 | down |
| EYA1 | NM_000503 | up | RNF223 | NM_001205252 | down |
| PER3 | NM_016831 | up | OR8J3 | NM_001004064 | down |
| GIPC2 | NM_017655 | up | OR6S1 | NM_001001968 | down |
| PLCL1 | NM_006226 | up | SIGLEC15 | NM_213602 | down |
| SMOC2 | NM_022138 | up | CTRB2 | NM_001025200 | down |
| TM4SF1 | NM_014220 | up | VENTX | NM_014468 | down |
| GRIK2 | NM_001166247 | up | IL3 | NM_000588 | down |
| LIMCH1 | NM_014988 | up | CST5 | NM_001900 | down |
| LPPR4 | NM_001166252 | up | GPR101 | NM_054021 | down |
| ACO1 | NM_002197 | up | VASH2 | NM_001136474 | down |
| CCL13 | NM_005408 | up | BMP3 | NM_001201 | down |
| FKBP2 | NM_001135208 | up | B9D2 | NM_030578 | down |
| KLHL12 | NM_021633 | up | HBM | NM_001003938 | down |
| NIPSNAP3A | NM_015469 | up | GALR3 | NM_003614 | down |
| RPL11 | NM_000975 | up | FGFBP2 | NM_031950 | down |
| MFN1 | NM_033540 | up | TRAJ38 | ENST00000390499 | down |
| DLD | NM_000108 | up | ELN | NM_000501 | down |
| TEK | NM_000459 | up | PCDH10 | NM_032961 | down |
| ITIH5 | NM_001001851 | up | HEY2 | NM_012259 | down |
| SLC16A7 | NM_004731 | up | NPNT | NM_001033047 | down |
| WWP1 | NM_007013 | up | C1QTNF1 | NM_198593 | down |
| PDPN | NM_001006624 | up | IER3 | NM_003897 | down |
| ACAD8 | NM_014384 | up | PLP2 | NM_002668 | down |
| GNA11 | NM_002067 | up | SUSD2 | NM_019601 | down |
| HIF1AN | NM_017902 | up | HBEGF | NM_001945 | down |
| RPS16 | NM_001020 | up | TGFB2 | NM_001135599 | down |
| TMEM159 | NM_020422 | up | SOST | NM_025237 | down |
| AGO1 | NM_012199 | up | MSS51 | NM_001024593 | down |
| SLC35B4 | NM_032826 | up | LOC101060662 | ENST00000527396 | down |
| ERAP1 | NM_001040458 | up | KRT18 | NM_000224 | down |
| KNOP1 | NM_001012991 | up | GJD2 | NM_020660 | down |
| AKAP1 | NM_001242902 | up | LUZP2 | NM_001009909 | down |
| SEL1L | NM_001244984 | up | CSPG4 | NM_001897 | down |
| TRIM27 | NM_006510 | up | FAM78B | NM_001017961 | down |
| STX7 | NM_003569 | up | CREB5 | NM_182898 | down |
| WLS | NM_001002292 | up | ST6GALNAC5 | NM_030965 | down |
| SLC35A5 | NM_017945 | up | MUC21 | NM_001010909 | down |
| ECSCR | NM_001077693 | up | UBALD1 | NM_145253 | down |
| PNRC2 | BC001959 | up |  |  |  |
| GM2A | NM_000405 | up |  |  |  |
| TPRG1L | NM_182752 | up |  |  |  |
| GOLPH3L | NM_018178 | up |  |  |  |
| TMED9 | NM_017510 | up |  |  |  |
| GALC | NM_000153 | up |  |  |  |
| AARS | NM_001605 | up |  |  |  |
| CDK4 | NM_000075 | up |  |  |  |
| HMG20A | NM_018200 | up |  |  |  |
| LOC100506388 | NM_001242780 | up |  |  |  |
| TBC1D14 | NM_001113361 | up |  |  |  |
| HNRNPA3 | NM_194247 | up |  |  |  |
| HARS2 | NM_012208 | up |  |  |  |
| TOMM20 | NM_014765 | up |  |  |  |
| CRK | NM_005206 | up |  |  |  |
| RPL38 | NM_000999 | up |  |  |  |
| ACOT2 | NM_006821 | up |  |  |  |
| MRFAP1 | NM_033296 | up |  |  |  |
| OSBP | NM_002556 | up |  |  |  |
| NMT1 | NM_021079 | up |  |  |  |
| ZNF350 | NM_021632 | up |  |  |  |
| PEPD | NM_000285 | up |  |  |  |
| RBM26 | NM_022118 | up |  |  |  |
| PTTG1IP | NM_004339 | up |  |  |  |
| ALG14 | NM_144988 | up |  |  |  |
| AMZ2 | NM_001033574 | up |  |  |  |
| ADCK3 | NM_020247 | up |  |  |  |
| CALM2 | NM_001743 | up |  |  |  |
| EHD4 | NM_139265 | up |  |  |  |
| BCKDHB | NM_000056 | up |  |  |  |
| IPP | NM_001145349 | up |  |  |  |
| SLC31A1 | NM_001859 | up |  |  |  |
| OS9 | NM_001017956 | up |  |  |  |
| SLC35E2B | NM_001110781 | up |  |  |  |
| NUDT3 | NM_006703 | up |  |  |  |
| CCL18 | NM_002988 | up |  |  |  |
| CP | NM_000096 | up |  |  |  |
| RELN | NM_173054 | up |  |  |  |
| SLC39A7 | NM_001077516 | up |  |  |  |
| VWA8 | NM_001009814 | up |  |  |  |
| CTSH | NM_004390 | up |  |  |  |
| RPS29 | NM_001032 | up |  |  |  |
| C20orf26 | NM_001167816 | up |  |  |  |
| PTPN3 | NM_002829 | up |  |  |  |
| PHB | NM_002634 | up |  |  |  |
| SMIM15 | NM_001048249 | up |  |  |  |
| SCN7A | NM_002976 | up |  |  |  |
| ETS1 | NM_001162422 | up |  |  |  |
| SLC39A8 | NM_001135146 | up |  |  |  |
| TRUB1 | NM_139169 | up |  |  |  |
| CDC40 | NM_015891 | up |  |  |  |
| KLRG1 | NM_005810 | up |  |  |  |
| GANAB | NM_198334 | up |  |  |  |
| ARFGEF2 | NM_006420 | up |  |  |  |
| LAMB1 | NM_002291 | up |  |  |  |
| HIBCH | NM_014362 | up |  |  |  |
| GIMAP8 | NM_175571 | up |  |  |  |
| ZDHHC13 | NM_019028 | up |  |  |  |
| CNDP2 | NM_001168499 | up |  |  |  |
| SRP14 | NM_003134 | up |  |  |  |
| ZNF134 | NM_003435 | up |  |  |  |
| TMEM60 | NM_032936 | up |  |  |  |
| IL3RA | NM_002183 | up |  |  |  |
| PREX2 | NM_024870 | up |  |  |  |
| FGF18 | NM_003862 | up |  |  |  |
| ZNF22 | NM_006963 | up |  |  |  |
| HADHA | NM_000182 | up |  |  |  |
| THRAP3 | NM_005119 | up |  |  |  |
| P4HA1 | NM_000917 | up |  |  |  |
| LIMD1 | NM_014240 | up |  |  |  |
| TPMT | NM_000367 | up |  |  |  |
| ZNF420 | NM_144689 | up |  |  |  |
| CD47 | NM_001777 | up |  |  |  |
| MTIF2 | NM_002453 | up |  |  |  |
| GNS | NM_002076 | up |  |  |  |
| SELRC1 | NM_023077 | up |  |  |  |
| GPRIN3 | NM_198281 | up |  |  |  |
| GALM | NM_138801 | up |  |  |  |
| POSTN | NM_001135934 | up |  |  |  |
| STK19 | NM_004197 | up |  |  |  |
| TGFBI | NM_000358 | up |  |  |  |
| C6 | NM_001115131 | up |  |  |  |
| FPGT-TNNI3K | NM_001112808 | up |  |  |  |
| PAIP2B | NM_020459 | up |  |  |  |
| FMO2 | NM_001460 | up |  |  |  |
| GIMAP7 | NM_153236 | up |  |  |  |
| HMGN3 | NM_001201362 | up |  |  |  |
| HIBADH | NM_152740 | up |  |  |  |
| RABL3 | NM_173825 | up |  |  |  |
| COX7C | NM_001867 | up |  |  |  |
| PLCL2 | NM_001144382 | up |  |  |  |
| VPS8 | NM_001009921 | up |  |  |  |
| SLIRP | NM_031210 | up |  |  |  |
| CLCN3 | NM_001243372 | up |  |  |  |
| TRIQK | NM_001171795 | up |  |  |  |
| C6orf211 | NM_024573 | up |  |  |  |
| HK2 | NM_000189 | up |  |  |  |
| MYCT1 | NM_025107 | up |  |  |  |
| KARS | NM_001130089 | up |  |  |  |
| TCEAL8 | NM_001006684 | up |  |  |  |
| HNRNPH2 | NM_001032393 | up |  |  |  |
| STOML2 | NM_013442 | up |  |  |  |
| TRIT1 | NM_017646 | up |  |  |  |
| RAPGEF4 | NM_001100397 | up |  |  |  |
| KIAA1143 | NM_020696 | up |  |  |  |
| MRPS31 | NM_005830 | up |  |  |  |
| PIK3R3 | NM_001114172 | up |  |  |  |
| GIMAP1-GIMAP5 | NM_001199577 | up |  |  |  |
| GRPEL1 | NM_025196 | up |  |  |  |
| TSR2 | NM_058163 | up |  |  |  |
| TMEM14C | NM_001165258 | up |  |  |  |
| SPCS2 | NM_014752 | up |  |  |  |
| CD163L1 | NM_174941 | up |  |  |  |
| CIRBP | NM_001280 | up |  |  |  |
| VTI1B | NM_006370 | up |  |  |  |
| TVP23B | NM_016078 | up |  |  |  |

**Supplemental Tab 4.** Male dysregulated mRNA of PVS from patients with LSP-AF

(Total mRNA: n=417; upregulated mRNA, n=215; downregulated mRNA, n=202)

| **Gene Symbol** | **Accession Number** | **Gene Feature** | **Gene Symbol** | **Accession Number** | **Gene Feature** | |
| --- | --- | --- | --- | --- | --- | --- |
| ACAT1 | NM_000019 | up | ACTA1 | NM_001100 | | down |
| AKAP11 | NM_016248 | up | ACTB | NM_001101 | | down |
| ANAPC16 | NM_001242546 | up | ACTG1 | NM_001199954 | | down |
| ANKRD20A2 | NM_001012421 | up | ACTG2 | NM_001199893 | | down |
| ANKRD20A3 | NM_001012419 | up | ADAMTS2 | NM_014244 | | down |
| ANP32D | NM_012404 | up | ADAP2 | NM_018404 | | down |
| AQP7 | NM_001170 | up | ADAT3 | NM_138422 | | down |
| ARPP19 | NM_006628 | up | AGAP1 | NM_001244888 | | down |
| AS3MT | NM_020682 | up | ARL17A | NM_001113738 | | down |
| ASAH1 | NM_001127505 | up | ATF4 | NM_182810 | | down |
| ATG14 | NM_014924 | up | AZIN1 | NM_015878 | | down |
| ATP5G1 | NM_001002027 | up | BCL9L | NM_182557 | | down |
| ATP5L2 | NM_001165877 | up | C11orf24 | NM_022338 | | down |
| ATP5O | NM_001697 | up | C12orf10 | NM_021640 | | down |
| ATP6V1D | NM_015994 | up | C18orf56 | NM_001012716 | | down |
| ATPAF1 | NM_001042546 | up | C1orf64 | NM_178840 | | down |
| BCAS2 | NM_005872 | up | C1QB | NM_000491 | | down |
| BEX4 | NM_001080425 | up | C1QC | NM_001114101 | | down |
| BST2 | NM_004335 | up | C3 | NM_000064 | | down |
| C14orf28 | NM_001017923 | up | C3orf70 | NM_001025266 | | down |
| C16orf87 | NM_001001436 | up | C9orf69 | NM_152833 | | down |
| C17orf75 | NM_022344 | up | CANT1 | NM_001159772 | | down |
| C1orf51 | NM_144697 | up | CBL | NM_005188 | | down |
| CCDC147 | NM_001008723 | up | CCR7 | NM_001838 | | down |
| CCDC43 | NM_144609 | up | CD248 | NM_020404 | | down |
| CCDC47 | NM_020198 | up | CD276 | NM_001024736 | | down |
| CCDC69 | NM_015621 | up | CD97 | NM_001025160 | | down |
| CDNF | NM_001029954 | up | CDA | NM_001785 | | down |
| CLGN | NM_001130675 | up | CEBPE | NM_001805 | | down |
| CLIP4 | NM_024692 | up | CERCAM | NM_016174 | | down |
| COQ7 | NM_001190983 | up | CLEC3B | NM_003278 | | down |
| COX17 | NM_005694 | up | CLU | NM_001831 | | down |
| COX5B | NM_001862 | up | COL1A1 | NM_000088 | | down |
| COX7B | NM_001866 | up | COL1A2 | NM_000089 | | down |
| CXorf61 | NM_001017978 | up | COL3A1 | NM_000090 | | down |
| CYB5B | NM_030579 | up | CPT1A | NM_001031847 | | down |
| DNAAF2 | NM_018139 | up | CREBBP | NM_001079846 | | down |
| EGF | NM_001178130 | up | CRIM1 | NM_016441 | | down |
| EIF4E3 | NM_001134651 | up | CRTC3 | NM_001042574 | | down |
| F5 | NM_000130 | up | CSRP1 | NM_001193570 | | down |
| FAM162B | NM_001085480 | up | DAP | NM_004394 | | down |
| FAM216B | NM_182508 | up | ECM1 | NM_001202858 | | down |
| FAM219B | NM_020447 | up | EDEM2 | NM_001145025 | | down |
| FAM21D | ENST00000311663 | up | EIF6 | NM_002212 | | down |
| FAM98A | NM_015475 | up | ELF4 | NM_001127197 | | down |
| FAM98B | NM_001042429 | up | EN1 | NM_001426 | | down |
| FNBP1L | NM_001024948 | up | ENC1 | NM_003633 | | down |
| GBP4 | NM_052941 | up | EPHA2 | NM_004431 | | down |
| GHITM | NM_014394 | up | FADS3 | NM_021727 | | down |
| GIMAP4 | NM_018326 | up | FAM168A | NM_015159 | | down |
| GLYAT | NM_005838 | up | FAM83D | NM_030919 | | down |
| GPD1L | NM_015141 | up | FBLN1 | NM_001996 | | down |
| GPR116 | NM_001098518 | up | FBLN5 | NM_006329 | | down |
| GPR52 | NM_005684 | up | FBN1 | NM_000138 | | down |
| GTF3C6 | NM_138408 | up | FGF23 | NM_020638 | | down |
| HAX1 | NM_001018837 | up | FLNA | NM_001456 | | down |
| HDHD2 | NM_032124 | up | FOXO6 | OTTHUMT00000015642 | | down |
| HIGD1A | NM_001099668 | up | FSTL3 | NM_005860 | | down |
| HIST1H1C | NM_005319 | up | FZD8 | NM_031866 | | down |
| HIST1H2BG | NM_003518 | up | GDF11 | NM_005811 | | down |
| HLA-DMA | NM_006120 | up | GGT5 | NM_004121 | | down |
| HLA-DRB4 | NM_021983 | up | GLI2 | NM_005270 | | down |
| HNRNPA2B1 | NM_002137 | up | GLI3 | NM_000168 | | down |
| HSBP1 | NM_001537 | up | GPR142 | NM_181790 | | down |
| HSF2 | NM_001135564 | up | GRAMD1A | NM_020895 | | down |
| IDH3B | NM_006899 | up | GRN | NM_002087 | | down |
| IER3IP1 | NM_016097 | up | GSTT1 | NM_000853 | | down |
| IFI27 | NM_001130080 | up | HCST | NM_014266 | | down |
| IFI44L | NM_006820 | up | HS3ST3A1 | NM_006042 | | down |
| IFIT3 | NM_001031683 | up | HSPB6 | NM_144617 | | down |
| IFIT5 | NM_012420 | up | HSPG2 | NM_005529 | | down |
| IGHD2-2 | ENST00000390591 | up | IFITM3 | NM_021034 | | down |
| IGHV2-5 | ENST00000390597 | up | IGFBP5 | NM_000599 | | down |
| ISCU | NM_014301 | up | INHBA | NM_002192 | | down |
| KDM3A | NM_001146688 | up | IRF2BPL | NM_024496 | | down |
| LACTB | NM_032857 | up | ISM1 | NM_080826 | | down |
| LACTB2 | NM_016027 | up | ITGB3 | NM_000212 | | down |
| LARP4 | NM_001170803 | up | ITLN1 | NM_017625 | | down |
| LIFR | NM_001127671 | up | LAMP5 | NM_001199897 | | down |
| LIN9 | NM_173083 | up | LGMN | NM_001008530 | | down |
| LRCH2 | NM_001243963 | up | LPP | NM_005578 | | down |
| LRRN3 | NM_001099658 | up | LPPR2 | NM_001170635 | | down |
| MBNL3 | NM_001170701 | up | LRP10 | NM_014045 | | down |
| MGEA5 | NM_001142434 | up | LRRC10B | NM_001145077 | | down |
| MINOS1 | NM_001032363 | up | LTBP3 | NM_001130144 | | down |
| MLF1 | NM_001130156 | up | MAF | NM_001031804 | | down |
| MRPL15 | NM_014175 | up | MEOX2 | NM_005924 | | down |
| MRPL50 | NM_019051 | up | MFSD7 | NM_032219 | | down |
| MRPL53 | NM_053050 | up | MGC10955 | ENST00000401851 | | down |
| MTIF3 | NM_001166262 | up | MN1 | NM_002430 | | down |
| NDUFA1 | NM_004541 | up | MSC | NM_005098 | | down |
| NDUFA6 | NM_002490 | up | MVP | NM_005115 | | down |
| NDUFA8 | NM_014222 | up | NAGLU | NM_000263 | | down |
| NDUFAF4 | NM_014165 | up | NAV1 | NM_001167738 | | down |
| NDUFB6 | NM_001199987 | up | NCS1 | NM_014286 | | down |
| NEDD1 | NM_001135175 | up | NEO1 | NM_002499 | | down |
| NMD3 | NM_015938 | up | NFIC | NM_001245002 | | down |
| NR1D2 | NM_001145425 | up | NFIX | NM_002501 | | down |
| NUCKS1 | NM_022731 | up | NPIPA1 | NM_006985 | | down |
| PCDH9 | NM_020403 | up | NPIPA5 | ENST00000427999 | | down |
| PCMTD2 | NM_001104925 | up | NR2F2 | NM_001145156 | | down |
| PDE7A | NM_001242318 | up | NT5DC2 | NM_001134231 | | down |
| PEX3 | NM_003630 | up | NT5DC3 | NM_001031701 | | down |
| PGK1 | NM_000291 | up | NTRK3 | NM_001007156 | | down |
| PIFO | NM_181643 | up | OLFML2B | NM_015441 | | down |
| PJA2 | NM_014819 | up | OR10H2 | NM_013939 | | down |
| PLA2G16 | NM_001128203 | up | OR10P1 | NM_206899 | | down |
| PLCXD3 | NM_001005473 | up | OR5AN1 | NM_001004729 | | down |
| PLN | NM_002667 | up | PABPC4L | NM_001114734 | | down |
| POF1B | NM_024921 | up | PAPPA | NM_002581 | | down |
| PPIL1 | NM_016059 | up | PDLIM7 | NM_005451 | | down |
| PPP1R9A | NM_001166161 | up | PFN1 | NM_005022 | | down |
| PPP4R2 | NM_174907 | up | PHLDA1 | NM_007350 | | down |
| PRKAG1 | NM_001206709 | up | PLAU | NM_001145031 | | down |
| PRKAR1A | NM_002734 | up | PLBD2 | NM_001159727 | | down |
| PRKG1 | NM_001098512 | up | PLXNA1 | NM_032242 | | down |
| PRMT3 | NM_001145166 | up | PPP1R18 | NM_133471 | | down |
| PRNP | NM_000311 | up | PRAF2 | NM_007213 | | down |
| PSMC5 | NM_001199163 | up | PRG4 | NM_001127708 | | down |
| PSME2 | NM_002818 | up | PROCR | NM_006404 | | down |
| PTGR1 | NM_001146108 | up | PRPS1L1 | NM_175886 | | down |
| PTGR2 | NM_001146154 | up | PRSS23 | NM_007173 | | down |
| PUS10 | NM_144709 | up | PRSS45 | NM_199183 | | down |
| PYROXD1 | NM_024854 | up | PTBP1 | NM_031991 | | down |
| RAB18 | NM_001256410 | up | PTGER4 | NM_000958 | | down |
| RAB21 | NM_014999 | up | PTPRE | NM_006504 | | down |
| RAB28 | NM_001017979 | up | PTPRG | NM_002841 | | down |
| RAD54B | NM_001205262 | up | PXN | NM_001080855 | | down |
| RBM41 | NM_001171080 | up | PYDC2 | NM_001083308 | | down |
| RBMXL3 | NM_001145346 | up | QSOX1 | NM_001004128 | | down |
| RCBTB1 | NM_018191 | up | RBM38 | NM_017495 | | down |
| RGCC | NM_014059 | up | RGAG4 | NM_001024455 | | down |
| RMDN1 | NM_016033 | up | S1PR2 | NM_004230 | | down |
| RNMT | NM_003799 | up | SBSPON | NM_153225 | | down |
| RPRD1A | NM_018170 | up | SCAMP2 | BC004385 | | down |
| RPS4XP21 | ENST00000469064 | up | SCAP | NM_012235 | | down |
| RRN3 | NM_018427 | up | SCUBE3 | NM_152753 | | down |
| RSAD2 | NM_080657 | up | SEBOX | NM_001080837 | | down |
| RTN4RL1 | NM_178568 | up | SERPING1 | NM_000062 | | down |
| SDPR | NM_004657 | up | SH3BGRL3 | NM_031286 | | down |
| SEC22B | NM_004892 | up | SIDT2 | NM_001040455 | | down |
| SFXN4 | NM_213649 | up | SIRPA | NM_001040022 | | down |
| SKA2 | NM_182620 | up | SLIT3 | NM_003062 | | down |
| SKA2P1 | ENST00000425592 | up | SLPI | NM_003064 | | down |
| SMC2 | NM_001042550 | up | SMAD3 | NM_001145102 | | down |
| SNURFL | ENST00000309296 | up | SOD3 | NM_003102 | | down |
| SOHLH2 | NM_017826 | up | SPON1 | NM_006108 | | down |
| STK33 | NM_030906 | up | SYNPO2 | NM_133477 | | down |
| TAF7 | NM_005642 | up | TAGLN | NM_001001522 | | down |
| TBCK | NM_001163436 | up | TEAD3 | NM_003214 | | down |
| TDP2 | NM_016614 | up | TMEM110-  MUSTN1 | NM_001198974 | | down |
| TIMM17A | NM_006335 | up | TMEM176A | NM_018487 | | down |
| TMEM106C | NM_001143841 | up | TMEM176B | NM_001101311 | | down |
| TMEM150C | NM_001080506 | up | TMEM89 | NM_001008269 | | down |
| TMEM256 | NM_152766 | up | TNS1 | NM_022648 | | down |
| TMEM71 | NM_001145153 | up | TP53INP2 | NM_021202 | | down |
| TNFRSF19 | NM_018647 | up | TPBGL | NM_001195528 | | down |
| TRIP4 | NM_016213 | up | TPM2 | NM_003289 | | down |
| TTC33 | NM_012382 | up | TRAF7 | NM_032271 | | down |
| TTF1 | NM_001205296 | up | TRBV10-1 | ENST00000390364 | | down |
| TXNIP | NM_006472 | up | TRIL | NM_014817 | | down |
| TXNRD1 | NM_001093771 | up | VASN | NM_138440 | | down |
| UQCR10 | NM_013387 | up | VGLL3 | NM_016206 | | down |
| UQCRB | NM_001199975 | up | WISP1 | NM_001204869 | | down |
| UQCRHL | NM_001089591 | up | XPNPEP2 | NM_003399 | | down |
| UROD | NM_000374 | up | XYLT1 | NM_022166 | | down |
| VDAC3 | NM_001135694 | up | ZCCHC14 | NM_015144 | | down |
| WDR12 | NM_018256 | up | ZDHHC18 | NM_032283 | | down |
| WDR60 | NM_018051 | up | ZMIZ1 | NM_020338 | | down |
| ZNF189 | NM_003452 | up | ZNF469 | NM_001127464 | | down |
| ZNF230 | NM_006300 | up | ZSWIM5 | NM_020883 | | down |
| ZNF404 | NM_001033719 | up |  | ENST00000372387 | | down |
| ZNF615 | NM_001199324 | up |  | ENST00000545087 | | down |
| ZNF649 | NM_023074 | up |  | ENST00000416839 | | down |
| ZNF675 | NM_138330 | up |  | ENST00000430695 | | down |
| ZNF844 | NM_001136501 | up |  | ENST00000339303 | | down |
| ZNF880 | NM_001145434 | up |  | BC012036 | | down |
| ZRANB2 | NM_005455 | up | POSTN | NM_001135934 | | down |
| FGFBP2 | NM_031950 | up | STK19 | NM_004197 | | down |
| TRAJ38 | ENST00000390499 | up | TGFBI | NM_000358 | | down |
| C6 | NM_001115131 | up | ELN | NM_000501 | | down |
| FPGT-  TNNI3K | NM_001112808 | up | PCDH10 | NM_032961 | | down |
| PAIP2B | NM_020459 | up | HEY2 | NM_012259 | | down |
| FMO2 | NM_001460 | up | NPNT | NM_001033047 | | down |
| GIMAP7 | NM_153236 | up | C1QTNF1 | NM_198593 | | down |
| HMGN3 | NM_001201362 | up | IER3 | NM_003897 | | down |
| HIBADH | NM_152740 | up | PLP2 | NM_002668 | | down |
| RABL3 | NM_173825 | up | SUSD2 | NM_019601 | | down |
| COX7C | NM_001867 | up | HBEGF | NM_001945 | | down |
| PLCL2 | NM_001144382 | up | TGFB2 | NM_001135599 | | down |
| VPS8 | NM_001009921 | up | SOST | NM_025237 | | down |
| SLIRP | NM_031210 | up | MSS51 | NM_001024593 | | down |
| CLCN3 | NM_001243372 | up | LOC101060662 | ENST00000527396 | | down |
| TRIQK | NM_001171795 | up | KRT18 | NM_000224 | | down |
| C6orf211 | NM_024573 | up | GJD2 | NM_020660 | | down |
| HK2 | NM_000189 | up | LUZP2 | NM_001009909 | | down |
| MYCT1 | NM_025107 | up | CSPG4 | NM_001897 | | down |
| KARS | NM_001130089 | up | FAM78B | NM_001017961 | | down |
| TCEAL8 | NM_001006684 | up | CREB5 | NM_182898 | | down |
| HNRNPH2 | NM_001032393 | up | ST6GALNAC5 | NM_030965 | | down |
| STOML2 | NM_013442 | up | MUC21 | NM_001010909 | | down |
| TRIT1 | NM_017646 | up | UBALD1 | NM_145253 | | down |
| RAPGEF4 | NM_001100397 | up |  |  | |  |
| KIAA1143 | NM_020696 | up |  |  | |  |
| MRPS31 | NM_005830 | up |  |  | |  |
| PIK3R3 | NM_001114172 | up |  |  | |  |
| GIMAP1-  GIMAP5 | NM_001199577 | up |  |  | |  |
| GRPEL1 | NM_025196 | up |  |  | |  |
| TSR2 | NM_058163 | up |  |  | |  |
| TMEM14C | NM_001165258 | up |  |  | |  |
| SPCS2 | NM_014752 | up |  |  | |  |
| CD163L1 | NM_174941 | up |  |  | |  |
| CIRBP | NM_001280 | up |  |  | |  |
| VTI1B | NM_006370 | up |  |  | |  |
| TVP23B | NM_016078 | up |  |  | |  |

**Supplemental Tab5. Female specific dysregulated mRNA of PVS from patients with LSP-AF (Total mRNA: n=459; upregulated mRNA, n=281; downregulated mRNA, n=178)**

| **Gene Symbol** | **Accession Number** | **Gene Feature** | **Gene Symbol** | **Accession**  **Number** | **Gene Feature** |
| --- | --- | --- | --- | --- | --- |
| SMYD2 | NM_020197 | up | HBA2 | ENST00000534957 | down |
| ATP1B4 | NM_001142447 | up | HBA1 | NM_000558 | down |
| MYL3 | NM_000258 | up | CH25H | NM_003956 | down |
| NPR3 | NM_000908 | up | MT2A | NM_005953 | down |
| CXCL12 | NM_000609 | up | NR4A1 | NM_001202233 | down |
| KCNIP2 | NM_014591 | up | ALAS2 | NM_000032 | down |
| CES2 | NM_003869 | up | ATF3 | NM_001030287 | down |
| SLN | NM_003063 | up | RNF122 | NM_024787 | down |
| ID1 | NM_002165 | up | OR1A1 | ENST00000304094 | down |
| MRPS14 | NM_022100 | up | C19orf71 | NM_001135580 | down |
| NRP1 | NM_001024628 | up | CEACAM8 | NM_001816 | down |
| FHL2 | BC014397 | up | RIPPLY3 | NM_018962 | down |
| VWF | NM_000552 | up | KLF2 | NM_016270 | down |
| ECHS1 | NM_004092 | up | HES1 | NM_005524 | down |
| APLNR | NM_005161 | up | B3GNT5 | NM_032047 | down |
| HIST1H4H | NM_003543 | up | RAI2 | NM_001172732 | down |
| ZNF224 | NM_013398 | up | ZFP36 | NM_003407 | down |
| FBXL4 | NM_012160 | up | JUNB | BC009465 | down |
| EHHADH | NM_001966 | up | GADD45B | NM_015675 | down |
| NUAK1 | NM_014840 | up | CXCL2 | NM_002089 | down |
| SPOP | NM_001007226 | up | CDKN1A | NM_000389 | down |
| ZNF223 | NM_013361 | up | PER1 | NM_002616 | down |
| CD93 | NM_012072 | up | PF4 | NM_002619 | down |
| SLC25A6 | NM_001636 | up | MIDN | NM_177401 | down |
| MALL | NM_005434 | up | CXCR3 | NM_001504 | down |
| NUDT21 | NM_007006 | up | IL8 | NM_000584 | down |
| IGFBP3 | NM_000598 | up | SOCS3 | NM_003955 | down |
| FREM1 | NM_144966 | up | RHOB | NM_004040 | down |
| ZNF546 | NM_178544 | up | HEMGN | NM_018437 | down |
| SSR3 | NM_007107 | up | GGN | NM_152657 | down |
| ATP6V0E1 | NM_003945 | up | PDZK1IP1 | NM_005764 | down |
| RTCB | NM_014306 | up | MT1A | NM_005946 | down |
| MED28 | NM_025205 | up | HBB | NM_000518 | down |
| SLC25A20 | NM_000387 | up | THBS1 | NM_003246 | down |
| ITM2C | NM_001012514 | up |  | ENST00000531638 | down |
| RAB30 | NM_014488 | up | EGR1 | NM_001964 | down |
| RNF141 | NM_016422 | up | PPBP | NM_002704 | down |
| NAGA | NM_000262 | up | BTG2 | NM_006763 | down |
| MRFAP1L1 | NM_203462 | up | AREG | NM_001657 | down |
| ZNF284 | NM_001037813 | up | SIK1 | NM_173354 | down |
| GIMAP6 | NM_001244071 | up | ADAMTS4 | NM_005099 | down |
| UTP3 | NM_020368 | up | IGLJ4 | ENST0000039036 | down |
| PHB2 | NM_001144831 | up | PLAUR | NM_001005376 | down |
| PAMR1 | NM_001001991 | up | JUND | NM_005354 | down |
| PIP4K2B | NM_003559 | up | FOSL2 | NM_005253 | down |
| ARMC1 | NM_018120 | up | EFCAB8 | OTTHUMT00000332145 | down |
| CARHSP1 | NM_014316 | up | TIGD5 | ENST00000504548 | down |
| MAGT1 | NM_032121 | up | SCXA | NM_001008271 | down |
| GUCA2B | NM_007102 | up | TNFAIP8L3 | NM_207381 | down |
| HNRNPUL2 | NM_001079559 | up | SLITRK1 | NM_052910 | down |
| YIPF5 | NM_001024947 | up | FOSB | NM_001114171 | down |
| DDX23 | NM_004818 | up | NR4A2 | NM_006186 | down |
| PRPF4 | NM_001244926 | up | FOS | NM_005252 | down |
| LOC100126582 | BC108270 | up | RGS1 | NM_002922 | down |
| AK4 | NM_001005353 | up | MT1M | NM_176870 | down |
| SLC33A1 | NM_001190992 | up | MT1X | NM_005952 | down |
| FAM198B | NM_001031700 | up | MT1G | NM_005950 | down |
| CALM3 | NM_005184 | up | IL6 | NM_000600 | down |
| CHURC1 | NM_001204063 | up | NR4A3 | NM_173199 | down |
| ZNF658B | BC137089 | up | RGS2 | NM_002923 | down |
| NUP43 | NM_198887 | up | NFKBIZ | NM_001005474 | down |
| HNRNPA1L2 | NM_001011724 | up | C5AR1 | NM_001736 | down |
| TAF15 | NM_139215 | up | DEFA1B | NM_001042500 | down |
| SNX12 | NM_001256185 | up | MT1F | NM_005949 | down |
| HEXA | NM_000520 | up | TNFAIP3 | NM_006290 | down |
| JAM2 | NM_021219 | up | CLEC4E | NM_014358 | down |
| MED1 | NM_004774 | up | TREM1 | NM_001242589 | down |
| METTL10 | NM_212554 | up | MT1H | NM_005951 | down |
| ABCG2 | NM_004827 | up | PRUNE2 | NM_015225 | down |
| SRP9 | NM_001130440 | up | MT1E | NM_175617 | down |
| PI4K2A | NM_018425 | up | NFIL3 | NM_005384 | down |
| CCDC121 | NM_001142683 | up | IL1B | NM_000576 | down |
| NIF3L1 | NM_001136039 | up | ADAMTS1 | NM_006988 | down |
| CNEP1R1 | NM_153261 | up | FPR1 | NM_001193306 | down |
| EXT2 | NM_000401 | up | DUSP1 | NM_004417 | down |
| PGGT1B | NM_005023 | up | CDR1 | NM_004065 | down |
| SMDT1 | NM_033318 | up | JUN | NM_002228 | down |
| PCDHB3 | NM_018937 | up | PRELP | NM_002725 | down |
| KIAA0100 | NM_014680 | up | LOC100127983 | NM_001190972 | down |
| MLEC | NM_014730 | up | MT1B | NM_005947 | down |
| RPL8 | NM_033301 | up | CCNH | NM_001199189 | down |
| HCN1 | NM_021072 | up | AFF2 | NM_001170628 | down |
| B3GALT2 | NM_003783 | up | CSRNP1 | NM_033027 | down |
| SVEP1 | NM_153366 | up |  | ENST00000454671 | down |
| KDR | NM_002253 | up | NLRP3 | NM_001079821 | down |
| QRSL1 | NM_018292 | up | MYC | NM_002467 | down |
| ARMCX1 | NM_016608 | up | KLF4 | NM_004235 | down |
| BNIP3 | NM_004052 | up | ACTA2 | NM_001141945 | down |
| MOXD1 | NM_015529 | up | XG | NM_001141919 | down |
| PRKCH | NM_006255 | up | SERPINE1 | NM_000602 | down |
| ATRNL1 | NM_207303 | up | CD177 | NM_020406 | down |
| CEP192 | NM_032142 | up | CD177P1 | ENST00000378007 | down |
| MRPL33 | NM_004891 | up | OLR1 | NM_001172632 | down |
| ZNF436 | NM_001077195 | up | C1orf138 | BC132992 | down |
| DHTKD1 | NM_018706 | up | CLDN1 | NM_021101 | down |
| M6PR | NM_001207024 | up | APOBEC3A | NM_145699 | down |
| AKAP10 | NM_007202 | up | MAT2A | NM_005911 | down |
| PCYOX1 | NM_016297 | up | SPRR1B | NM_003125 | down |
| ZBTB6 | NM_006626 | up | SHC4 | NM_203349 | down |
| PSMB3 | NM_002795 | up | CEBPD | NM_005195 | down |
| WDR5B | NM_019069 | up | TSPYL2 | NM_022117 | down |
| TXNDC11 | NM_015914 | up | BHLHE40 | NM_003670 | down |
| LBH | NM_030915 | up | ARG1 | NM_000045 | down |
| ACLY | NM_001096 | up | RGS16 | NM_002928 | down |
| PGAM4 | NM_001029891 | up | SPANXC | NM_022661 | down |
| EEF1G | NM_001404 | up | MAFF | NM_001161572 | down |
| TNPO3 | NM_001191028 | up | TRAJ14 | ENST00000390523 | down |
| ABHD10 | NM_018394 | up | IER2 | NM_004907 | down |
| DPH5 | NM_001077394 | up | PPP1R14A | NM_001243947 | down |
| ZNF426 | NM_024106 | up | TMEM75 | BC137383 | down |
| TMEM203 | BC009461 | up | WFDC1 | NM_021197 | down |
| PSAP | NM_001042465 | up | HEYL | NM_014571 | down |
| GGA2 | NM_015044 | up | OR1K1 | NM_080859 | down |
| AASDHPPT | NM_015423 | up | TIPARP | NM_001184717 | down |
| JAM3 | NM_001205329 | up | ZNF831 | NM_178457 | down |
| ZNF75A | NM_153028 | up | CA1 | NM_001164830 | down |
| AHCYL1 | NM_001242673 | up |  | ENST00000381466 | down |
| AP3M1 | NM_012095 | up | IFRD1 | NM_001007245 | down |
| ZIK1 | NM_001010879 | up | ADAMTS8 | NM_007037 | down |
| PARP1 | NM_001618 | up | NAP1L3 | NM_004538 | down |
| METTL3 | NM_019852 | up | P2RX1 | NM_002558 | down |
| SLC2A12 | NM_145176 | up | EFHC2 | NM_025184 | down |
| UGT2B4 | NM_021139 | up | HBZ | NM_005332 | down |
| CMA1 | BC069370 | up | MEGF9 | NM_001080497 | down |
| ANGPTL2 | NM_012098 | up | C10orf10 | NM_007021 | down |
| TM4SF18 | NM_138786 | up | OR4S1 | NM_001004725 | down |
| PDK1 | NM_002610 | up | PADI4 | NM_012387 | down |
| CYP1B1 | NM_000104 | up | NEDD9 | NM_006403 | down |
| FOLR2 | NM_000803 | up | FOXQ1 | NM_033260 | down |
| TXNDC5 | NM_001145549 | up | KBTBD13 | NM_001101362 | down |
| RPS15 | NM_001018 | up | CTRB1 | NM_001906 | down |
| LRRC6 | NM_012472 | up | IGLJ7 | ENST00000390330 | down |
| ARMCX3 | NM_177948 | up | CAMP | NM_004345 | down |
| EBF2 | NM_022659 | up | KRTAP1-1 | ENST00000543328 | down |
| ALDH1A2 | NM_001206897 | up | GDF2 | NM_016204 | down |
| SPR | NM_003124 | up | ZC3H12A | NM_025079 | down |
| KRTAP19-4 | NM_181610 | up | KCNMB1 | NM_004137 | down |
| COL6A6 | NM_001102608 | up | ASAP2 | NM_001135191 | down |
| ZNF146 | NM_001099638 | up | OXER1 | NM_148962 | down |
| EBF3 | NM_001005463 | up | FKBPL | NM_022110 | down |
| PXMP2 | NM_018663 | up | TMEM86B | NM_173804 | down |
| SDHD | NM_003002 | up | C1QL4 | NM_001008223 | down |
| VPS35 | NM_018206 | up | OR52B6 | NM_001005162 | down |
| EEF2 | NM_001961 | up | USP17L1P | OTTHUMT00000318957 | down |
| ZNF552 | NM_024762 | up | AXIN2 | NM_004655 | down |
| SCO1 | NM_004589 | up | KLHDC7B | NM_138433 | down |
| WDR3 | NM_006784 | up | CDCA5 | ENST00000438878 | down |
| MECOM | NM_001105077 | up | OR6C1 | NM_001005182 | down |
| LCLAT1 | NM_182551 | up | OR2L3 | NM_001004687 | down |
| IMPAD1 | NM_017813 | up | VASP | NM_003370 | down |
| MUL1 | NM_024544 | up | APOF | NM_001638 | down |
| ZFP14 | NM_020917 | up | SFN | NM_006142 | down |
| NOP9 | NM_174913 | up | C10orf25 | NM_001039380 | down |
| PREB | NM_013388 | up | DEGS2 | NM_206918 | down |
| RNF185 | NM_001135825 | up | TMEM240 | NM_001114748 | down |
| MTX3 | NM_001010891 | up | PLK3 | NM_004073 | down |
| RRAGC | NM_022157 | up | C9orf106 | NM_001012715 | down |
| C6orf106 | NM_022758 | up | FATE1 | NM_033085 | down |
| SLC4A4 | NM_003759 | up | SPATC1 | NM_001134374 | down |
| KLHL36 | NM_024731 | up | TSSK3 | NM_052841 | down |
| SLC5A1 | NM_000343 | up | THBD | NM_000361 | down |
| ENPP2 | NM_001040092 | up | EXPH5 | NM_015065 | down |
| EYA1 | NM_000503 | up | RNF223 | NM_001205252 | down |
| PER3 | NM_016831 | up | OR8J3 | NM_001004064 | down |
| GIPC2 | NM_017655 | up | OR6S1 | NM_001001968 | down |
| PLCL1 | NM_006226 | up | SIGLEC15 | NM_213602 | down |
| SMOC2 | NM_022138 | up | CTRB2 | NM_001025200 | down |
| TM4SF1 | NM_014220 | up | VENTX | NM_014468 | down |
| GRIK2 | NM_001166247 | up | IL3 | NM_000588 | down |
| LIMCH1 | NM_014988 | up | CST5 | NM_001900 | down |
| LPPR4 | NM_001166252 | up | GPR101 | NM_054021 | down |
| ACO1 | NM_002197 | up | VASH2 | NM_001136474 | down |
| CCL13 | NM_005408 | up | BMP3 | NM_001201 | down |
| FKBP2 | NM_001135208 | up | B9D2 | NM_030578 | down |
| KLHL12 | NM_021633 | up | HBM | NM_001003938 | down |
| NIPSNAP3A | NM_015469 | up | GALR3 | NM_003614 | down |
| RPL11 | NM_000975 | up | FGFBP2 | NM_031950 | down |
| MFN1 | NM_033540 | up | TRAJ38 | ENST00000390499 | down |
| DLD | NM_000108 | up |  |  |  |
| TEK | NM_000459 | up |  |  |  |
| ITIH5 | NM_001001851 | up |  |  |  |
| SLC16A7 | NM_004731 | up |  |  |  |
| WWP1 | NM_007013 | up |  |  |  |
| PDPN | NM_001006624 | up |  |  |  |
| ACAD8 | NM_014384 | up |  |  |  |
| GNA11 | NM_002067 | up |  |  |  |
| HIF1AN | NM_017902 | up |  |  |  |
| RPS16 | NM_001020 | up |  |  |  |
| TMEM159 | NM_020422 | up |  |  |  |
| AGO1 | NM_012199 | up |  |  |  |
| SLC35B4 | NM_032826 | up |  |  |  |
| ERAP1 | NM_001040458 | up |  |  |  |
| KNOP1 | NM_001012991 | up |  |  |  |
| AKAP1 | NM_001242902 | up |  |  |  |
| SEL1L | NM_001244984 | up |  |  |  |
| TRIM27 | NM_006510 | up |  |  |  |
| STX7 | NM_003569 | up |  |  |  |
| WLS | NM_001002292 | up |  |  |  |
| SLC35A5 | NM_017945 | up |  |  |  |
| ECSCR | NM_001077693 | up |  |  |  |
| PNRC2 | BC001959 | up |  |  |  |
| GM2A | NM_000405 | up |  |  |  |
| TPRG1L | NM_182752 | up |  |  |  |
| GOLPH3L | NM_018178 | up |  |  |  |
| TMED9 | NM_017510 | up |  |  |  |
| GALC | NM_000153 | up |  |  |  |
| AARS | NM_001605 | up |  |  |  |
| CDK4 | NM_000075 | up |  |  |  |
| HMG20A | NM_018200 | up |  |  |  |
| LOC100506388 | NM_001242780 | up |  |  |  |
| TBC1D14 | NM_001113361 | up |  |  |  |
| HNRNPA3 | NM_194247 | up |  |  |  |
| HARS2 | NM_012208 | up |  |  |  |
| TOMM20 | NM_014765 | up |  |  |  |
| CRK | NM_005206 | up |  |  |  |
| RPL38 | NM_000999 | up |  |  |  |
| ACOT2 | NM_006821 | up |  |  |  |
| MRFAP1 | NM_033296 | up |  |  |  |
| OSBP | NM_002556 | up |  |  |  |
| NMT1 | NM_021079 | up |  |  |  |
| ZNF350 | NM_021632 | up |  |  |  |
| PEPD | NM_000285 | up |  |  |  |
| RBM26 | NM_022118 | up |  |  |  |
| PTTG1IP | NM_004339 | up |  |  |  |
| ALG14 | NM_144988 | up |  |  |  |
| AMZ2 | NM_001033574 | up |  |  |  |
| ADCK3 | NM_020247 | up |  |  |  |
| CALM2 | NM_001743 | up |  |  |  |
| EHD4 | NM_139265 | up |  |  |  |
| BCKDHB | NM_000056 | up |  |  |  |
| IPP | NM_001145349 | up |  |  |  |
| SLC31A1 | NM_001859 | up |  |  |  |
| OS9 | NM_001017956 | up |  |  |  |
| SLC35E2B | NM_001110781 | up |  |  |  |
| NUDT3 | NM_006703 | up |  |  |  |
| CCL18 | NM_002988 | up |  |  |  |
| CP | NM_000096 | up |  |  |  |
| RELN | NM_173054 | up |  |  |  |
| SLC39A7 | NM_001077516 | up |  |  |  |
| VWA8 | NM_001009814 | up |  |  |  |
| CTSH | NM_004390 | up |  |  |  |
| RPS29 | NM_001032 | up |  |  |  |
| C20orf26 | NM_001167816 | up |  |  |  |
| PTPN3 | NM_002829 | up |  |  |  |
| PHB | NM_002634 | up |  |  |  |
| SMIM15 | NM_001048249 | up |  |  |  |
| SCN7A | NM_002976 | up |  |  |  |
| ETS1 | NM_001162422 | up |  |  |  |
| SLC39A8 | NM_001135146 | up |  |  |  |
| TRUB1 | NM_139169 | up |  |  |  |
| CDC40 | NM_015891 | up |  |  |  |
| KLRG1 | NM_005810 | up |  |  |  |
| GANAB | NM_198334 | up |  |  |  |
| ARFGEF2 | NM_006420 | up |  |  |  |
| LAMB1 | NM_002291 | up |  |  |  |
| HIBCH | NM_014362 | up |  |  |  |
| GIMAP8 | NM_175571 | up |  |  |  |
| ZDHHC13 | NM_019028 | up |  |  |  |
| CNDP2 | NM_001168499 | up |  |  |  |
| SRP14 | NM_003134 | up |  |  |  |
| ZNF134 | NM_003435 | up |  |  |  |
| TMEM60 | NM_032936 | up |  |  |  |
| IL3RA | NM_002183 | up |  |  |  |
| PREX2 | NM_024870 | up |  |  |  |
| FGF18 | NM_003862 | up |  |  |  |
| ZNF22 | NM_006963 | up |  |  |  |
| HADHA | NM_000182 | up |  |  |  |
| THRAP3 | NM_005119 | up |  |  |  |
| P4HA1 | NM_000917 | up |  |  |  |
| LIMD1 | NM_014240 | up |  |  |  |
| TPMT | NM_000367 | up |  |  |  |
| ZNF420 | NM_144689 | up |  |  |  |
| CD47 | NM_001777 | up |  |  |  |
| MTIF2 | NM_002453 | up |  |  |  |
| GNS | NM_002076 | up |  |  |  |
| SELRC1 | NM_023077 | up |  |  |  |
| GPRIN3 | NM_198281 | up |  |  |  |
| GALM | NM_138801 | up |  |  |  |
| POSTN | NM_001135934 | up |  |  |  |
| STK19 | NM_004197 | up |  |  |  |
| TGFBI | NM_000358 | up |  |  |  |

**Supplemental Tab 6.** Overlapping mRNA between male and female dysregulated mRNA of PVS from patients with LSP-AF (Total mRNA: n=57; upregulated mRNA, n=35; downregulated mRNA, n=22)

| **Gene**  **Symbol** | **Accession Number** | **Gene Feature** | **Gene**  **Symbol** | **Accession Number** | **Gene Feature** |
| --- | --- | --- | --- | --- | --- |
| C6 | NM_001115131 | up | ELN | NM_000501 | down |
| FPGT-TNNI3K | NM_001112808 | up | PCDH10 | NM_032961 | down |
| PAIP2B | NM_020459 | up | HEY2 | NM_012259 | down |
| FMO2 | NM_001460 | up | NPNT | NM_001033047 | down |
| GIMAP7 | NM_153236 | up | C1QTNF1 | NM_198593 | down |
| HMGN3 | NM_001201362 | up | IER3 | NM_003897 | down |
| HIBADH | NM_152740 | up | PLP2 | NM_002668 | down |
| RABL3 | NM_173825 | up | SUSD2 | NM_019601 | down |
| COX7C | NM_001867 | up | HBEGF | NM_001945 | down |
| PLCL2 | NM_001144382 | up | TGFB2 | NM_001135599 | down |
| VPS8 | NM_001009921 | up | SOST | NM_025237 | down |
| SLIRP | NM_031210 | up | MSS51 | NM_001024593 | down |
| CLCN3 | NM_001243372 | up | LOC101060662 | ENST00000527396 | down |
| TRIQK | NM_001171795 | up | KRT18 | NM_000224 | down |
| C6orf211 | NM_024573 | up | GJD2 | NM_020660 | down |
| HK2 | NM_000189 | up | LUZP2 | NM_001009909 | down |
| MYCT1 | NM_025107 | up | CSPG4 | NM_001897 | down |
| KARS | NM_001130089 | up | FAM78B | NM_001017961 | down |
| TCEAL8 | NM_001006684 | up | CREB5 | NM_182898 | down |
| HNRNPH2 | NM_001032393 | up | ST6GALNAC5 | NM_030965 | down |
| STOML2 | NM_013442 | up | MUC21 | NM_001010909 | down |
| TRIT1 | NM_017646 | up | UBALD1 | NM_145253 | down |
| RAPGEF4 | NM_001100397 | up |  |  |  |
| KIAA1143 | NM_020696 | up |  |  |  |
| MRPS31 | NM_005830 | up |  |  |  |
| PIK3R3 | NM_001114172 | up |  |  |  |
| GIMAP1-GIMAP5 | NM_001199577 | up |  |  |  |
| GRPEL1 | NM_025196 | up |  |  |  |
| TSR2 | NM_058163 | up |  |  |  |
| TMEM14C | NM_001165258 | up |  |  |  |
| SPCS2 | NM_014752 | up |  |  |  |
| CD163L1 | NM_174941 | up |  |  |  |
| CIRBP | NM_001280 | up |  |  |  |
| VTI1B | NM_006370 | up |  |  |  |
| TVP23B | NM_016078 | up |  |  |  |

**Supplemental Tab 7. Male specific dysregulated mRNA of PVS from patients with LSP-AF (Total mRNA: n=360; upregulated mRNA, n=180; downregulated mRNA, n=180)**

| **Gene**  **Symbol** | **Accession Number** | **Gene Feature** | **Gene**  **Symbol** | **Accession**  **Number** | **Gene Feature** |
| --- | --- | --- | --- | --- | --- |
| ACAT1 | NM_000019 | up | ACTA1 | NM_001100 | down |
| AKAP11 | NM_016248 | up | ACTB | NM_001101 | down |
| ANAPC16 | NM_001242546 | up | ACTG1 | NM_001199954 | down |
| ANKRD20A2 | NM_001012421 | up | ACTG2 | NM_001199893 | down |
| ANKRD20A3 | NM_001012419 | up | ADAMTS2 | NM_014244 | down |
| ANP32D | NM_012404 | up | ADAP2 | NM_018404 | down |
| AQP7 | NM_001170 | up | ADAT3 | NM_138422 | down |
| ARPP19 | NM_006628 | up | AGAP1 | NM_001244888 | down |
| AS3MT | NM_020682 | up | ARL17A | NM_001113738 | down |
| ASAH1 | NM_001127505 | up | ATF4 | NM_182810 | down |
| ATG14 | NM_014924 | up | AZIN1 | NM_015878 | down |
| ATP5G1 | NM_001002027 | up | BCL9L | NM_182557 | down |
| ATP5L2 | NM_001165877 | up | C11orf24 | NM_022338 | down |
| ATP5O | NM_001697 | up | C12orf10 | NM_021640 | down |
| ATP6V1D | NM_015994 | up | C18orf56 | NM_001012716 | down |
| ATPAF1 | NM_001042546 | up | C1orf64 | NM_178840 | down |
| BCAS2 | NM_005872 | up | C1QB | NM_000491 | down |
| BEX4 | NM_001080425 | up | C1QC | NM_001114101 | down |
| BST2 | NM_004335 | up | C3 | NM_000064 | down |
| C14orf28 | NM_001017923 | up | C3orf70 | NM_001025266 | down |
| C16orf87 | NM_001001436 | up | C9orf69 | NM_152833 | down |
| C17orf75 | NM_022344 | up | CANT1 | NM_001159772 | down |
| C1orf51 | NM_144697 | up | CBL | NM_005188 | down |
| CCDC147 | NM_001008723 | up | CCR7 | NM_001838 | down |
| CCDC43 | NM_144609 | up | CD248 | NM_020404 | down |
| CCDC47 | NM_020198 | up | CD276 | NM_001024736 | down |
| CCDC69 | NM_015621 | up | CD97 | NM_001025160 | down |
| CDNF | NM_001029954 | up | CDA | NM_001785 | down |
| CLGN | NM_001130675 | up | CEBPE | NM_001805 | down |
| CLIP4 | NM_024692 | up | CERCAM | NM_016174 | down |
| COQ7 | NM_001190983 | up | CLEC3B | NM_003278 | down |
| COX17 | NM_005694 | up | CLU | NM_001831 | down |
| COX5B | NM_001862 | up | COL1A1 | NM_000088 | down |
| COX7B | NM_001866 | up | COL1A2 | NM_000089 | down |
| CXorf61 | NM_001017978 | up | COL3A1 | NM_000090 | down |
| CYB5B | NM_030579 | up | CPT1A | NM_001031847 | down |
| DNAAF2 | NM_018139 | up | CREBBP | NM_001079846 | down |
| EGF | NM_001178130 | up | CRIM1 | NM_016441 | down |
| EIF4E3 | NM_001134651 | up | CRTC3 | NM_001042574 | down |
| F5 | NM_000130 | up | CSRP1 | NM_001193570 | down |
| FAM162B | NM_001085480 | up | DAP | NM_004394 | down |
| FAM216B | NM_182508 | up | ECM1 | NM_001202858 | down |
| FAM219B | NM_020447 | up | EDEM2 | NM_001145025 | down |
| FAM21D | ENST00000311663 | up | EIF6 | NM_002212 | down |
| FAM98A | NM_015475 | up | ELF4 | NM_001127197 | down |
| FAM98B | NM_001042429 | up | EN1 | NM_001426 | down |
| FNBP1L | NM_001024948 | up | ENC1 | NM_003633 | down |
| GBP4 | NM_052941 | up | EPHA2 | NM_004431 | down |
| GHITM | NM_014394 | up | FADS3 | NM_021727 | down |
| GIMAP4 | NM_018326 | up | FAM168A | NM_015159 | down |
| GLYAT | NM_005838 | up | FAM83D | NM_030919 | down |
| GPD1L | NM_015141 | up | FBLN1 | NM_001996 | down |
| GPR116 | NM_001098518 | up | FBLN5 | NM_006329 | down |
| GPR52 | NM_005684 | up | FBN1 | NM_000138 | down |
| GTF3C6 | NM_138408 | up | FGF23 | NM_020638 | down |
| HAX1 | NM_001018837 | up | FLNA | NM_001456 | down |
| HDHD2 | NM_032124 | up | FOXO6 | OTTHUMT00000015642 | down |
| HIGD1A | NM_001099668 | up | FSTL3 | NM_005860 | down |
| HIST1H1C | NM_005319 | up | FZD8 | NM_031866 | down |
| HIST1H2BG | NM_003518 | up | GDF11 | NM_005811 | down |
| HLA-  DMA | NM_006120 | up | GGT5 | NM_004121 | down |
| HLA-  DRB4 | NM_021983 | up | GLI2 | NM_005270 | down |
| HNRNPA2B1 | NM_002137 | up | GLI3 | NM_000168 | down |
| HSBP1 | NM_001537 | up | GPR142 | NM_181790 | down |
| HSF2 | NM_001135564 | up | GRAMD1A | NM_020895 | down |
| IDH3B | NM_006899 | up | GRN | NM_002087 | down |
| IER3IP1 | NM_016097 | up | GSTT1 | NM_000853 | down |
| IFI27 | NM_001130080 | up | HCST | NM_014266 | down |
| IFI44L | NM_006820 | up | HS3ST3A1 | NM_006042 | down |
| IFIT3 | NM_001031683 | up | HSPB6 | NM_144617 | down |
| IFIT5 | NM_012420 | up | HSPG2 | NM_005529 | down |
| IGHD2-2 | ENST00000390591 | up | IFITM3 | NM_021034 | down |
| IGHV2-5 | ENST00000390597 | up | IGFBP5 | NM_000599 | down |
| ISCU | NM_014301 | up | INHBA | NM_002192 | down |
| KDM3A | NM_001146688 | up | IRF2BPL | NM_024496 | down |
| LACTB | NM_032857 | up | ISM1 | NM_080826 | down |
| LACTB2 | NM_016027 | up | ITGB3 | NM_000212 | down |
| LARP4 | NM_001170803 | up | ITLN1 | NM_017625 | down |
| LIFR | NM_001127671 | up | LAMP5 | NM_001199897 | down |
| LIN9 | NM_173083 | up | LGMN | NM_001008530 | down |
| LRCH2 | NM_001243963 | up | LPP | NM_005578 | down |
| LRRN3 | NM_001099658 | up | LPPR2 | NM_001170635 | down |
| MBNL3 | NM_001170701 | up | LRP10 | NM_014045 | down |
| MGEA5 | NM_001142434 | up | LRRC10B | NM_001145077 | down |
| MINOS1 | NM_001032363 | up | LTBP3 | NM_001130144 | down |
| MLF1 | NM_001130156 | up | MAF | NM_001031804 | down |
| MRPL15 | NM_014175 | up | MEOX2 | NM_005924 | down |
| MRPL50 | NM_019051 | up | MFSD7 | NM_032219 | down |
| MRPL53 | NM_053050 | up | MGC10955 | ENST00000401851 | down |
| MTIF3 | NM_001166262 | up | MN1 | NM_002430 | down |
| NDUFA1 | NM_004541 | up | MSC | NM_005098 | down |
| NDUFA6 | NM_002490 | up | MVP | NM_005115 | down |
| NDUFA8 | NM_014222 | up | NAGLU | NM_000263 | down |
| NDUFAF4 | NM_014165 | up | NAV1 | NM_001167738 | down |
| NDUFB6 | NM_001199987 | up | NCS1 | NM_014286 | down |
| NEDD1 | NM_001135175 | up | NEO1 | NM_002499 | down |
| NMD3 | NM_015938 | up | NFIC | NM_001245002 | down |
| NR1D2 | NM_001145425 | up | NFIX | NM_002501 | down |
| NUCKS1 | NM_022731 | up | NPIPA1 | NM_006985 | down |
| PCDH9 | NM_020403 | up | NPIPA5 | ENST00000427999 | down |
| PCMTD2 | NM_001104925 | up | NR2F2 | NM_001145156 | down |
| PDE7A | NM_001242318 | up | NT5DC2 | NM_001134231 | down |
| PEX3 | NM_003630 | up | NT5DC3 | NM_001031701 | down |
| PGK1 | NM_000291 | up | NTRK3 | NM_001007156 | down |
| PIFO | NM_181643 | up | OLFML2B | NM_015441 | down |
| PJA2 | NM_014819 | up | OR10H2 | NM_013939 | down |
| PLA2G16 | NM_001128203 | up | OR10P1 | NM_206899 | down |
| PLCXD3 | NM_001005473 | up | OR5AN1 | NM_001004729 | down |
| PLN | NM_002667 | up | PABPC4L | NM_001114734 | down |
| POF1B | NM_024921 | up | PAPPA | NM_002581 | down |
| PPIL1 | NM_016059 | up | PDLIM7 | NM_005451 | down |
| PPP1R9A | NM_001166161 | up | PFN1 | NM_005022 | down |
| PPP4R2 | NM_174907 | up | PHLDA1 | NM_007350 | down |
| PRKAG1 | NM_001206709 | up | PLAU | NM_001145031 | down |
| PRKAR1A | NM_002734 | up | PLBD2 | NM_001159727 | down |
| PRKG1 | NM_001098512 | up | PLXNA1 | NM_032242 | down |
| PRMT3 | NM_001145166 | up | PPP1R18 | NM_133471 | down |
| PRNP | NM_000311 | up | PRAF2 | NM_007213 | down |
| PSMC5 | NM_001199163 | up | PRG4 | NM_001127708 | down |
| PSME2 | NM_002818 | up | PROCR | NM_006404 | down |
| PTGR1 | NM_001146108 | up | PRPS1L1 | NM_175886 | down |
| PTGR2 | NM_001146154 | up | PRSS23 | NM_007173 | down |
| PUS10 | NM_144709 | up | PRSS45 | NM_199183 | down |
| PYROXD1 | NM_024854 | up | PTBP1 | NM_031991 | down |
| RAB18 | NM_001256410 | up | PTGER4 | NM_000958 | down |
| RAB21 | NM_014999 | up | PTPRE | NM_006504 | down |
| RAB28 | NM_001017979 | up | PTPRG | NM_002841 | down |
| RAD54B | NM_001205262 | up | PXN | NM_001080855 | down |
| RBM41 | NM_001171080 | up | PYDC2 | NM_001083308 | down |
| RBMXL3 | NM_001145346 | up | QSOX1 | NM_001004128 | down |
| RCBTB1 | NM_018191 | up | RBM38 | NM_017495 | down |
| RGCC | NM_014059 | up | RGAG4 | NM_001024455 | down |
| RMDN1 | NM_016033 | up | S1PR2 | NM_004230 | down |
| RNMT | NM_003799 | up | SBSPON | NM_153225 | down |
| RPRD1A | NM_018170 | up | SCAMP2 | BC004385 | down |
| RPS4XP21 | ENST00000469064 | up | SCAP | NM_012235 | down |
| RRN3 | NM_018427 | up | SCUBE3 | NM_152753 | down |
| RSAD2 | NM_080657 | up | SEBOX | NM_001080837 | down |
| RTN4RL1 | NM_178568 | up | SERPING1 | NM_000062 | down |
| SDPR | NM_004657 | up | SH3BGRL3 | NM_031286 | down |
| SEC22B | NM_004892 | up | SIDT2 | NM_001040455 | down |
| SFXN4 | NM_213649 | up | SIRPA | NM_001040022 | down |
| SKA2 | NM_182620 | up | SLIT3 | NM_003062 | down |
| SKA2P1 | ENST00000425592 | up | SLPI | NM_003064 | down |
| SMC2 | NM_001042550 | up | SMAD3 | NM_001145102 | down |
| SNURFL | ENST00000309296 | up | SOD3 | NM_003102 | down |
| SOHLH2 | NM_017826 | up | SPON1 | NM_006108 | down |
| STK33 | NM_030906 | up | SYNPO2 | NM_133477 | down |
| TAF7 | NM_005642 | up | TAGLN | NM_001001522 | down |
| TBCK | NM_001163436 | up | TEAD3 | NM_003214 | down |
| TDP2 | NM_016614 | up | TMEM110-MUSTN1 | NM_001198974 | down |
| TIMM17A | NM_006335 | up | TMEM176A | NM_018487 | down |
| TMEM106C | NM_001143841 | up | TMEM176B | NM_001101311 | down |
| TMEM150C | NM_001080506 | up | TMEM89 | NM_001008269 | down |
| TMEM256 | NM_152766 | up | TNS1 | NM_022648 | down |
| TMEM71 | NM_001145153 | up | TP53INP2 | NM_021202 | down |
| TNFRSF19 | NM_018647 | up | TPBGL | NM_001195528 | down |
| TRIP4 | NM_016213 | up | TPM2 | NM_003289 | down |
| TTC33 | NM_012382 | up | TRAF7 | NM_032271 | down |
| TTF1 | NM_001205296 | up | TRBV10-1 | ENST00000390364 | down |
| TXNIP | NM_006472 | up | TRIL | NM_014817 | down |
| TXNRD1 | NM_001093771 | up | VASN | NM_138440 | down |
| UQCR10 | NM_013387 | up | VGLL3 | NM_016206 | down |
| UQCRB | NM_001199975 | up | WISP1 | NM_001204869 | down |
| UQCRHL | NM_001089591 | up | XPNPEP2 | NM_003399 | down |
| UROD | NM_000374 | up | XYLT1 | NM_022166 | down |
| VDAC3 | NM_001135694 | up | ZCCHC14 | NM_015144 | down |
| WDR12 | NM_018256 | up | ZDHHC18 | NM_032283 | down |
| WDR60 | NM_018051 | up | ZMIZ1 | NM_020338 | down |
| ZNF189 | NM_003452 | up | ZNF469 | NM_001127464 | down |
| ZNF230 | NM_006300 | up | ZSWIM5 | NM_020883 | down |
| ZNF404 | NM_001033719 | up |  | ENST00000372387 | down |
| ZNF615 | NM_001199324 | up |  | ENST00000545087 | down |
| ZNF649 | NM_023074 | up |  | ENST00000416839 | down |
| ZNF675 | NM_138330 | up |  | ENST00000430695 | down |
| ZNF844 | NM_001136501 | up |  | ENST00000339303 | down |
| ZNF880 | NM_001145434 | up |  | BC012036 | down |
| ZRANB2 | NM_005455 | up | POSTN | NM_001135934 | down |
| FGFBP2 | NM_031950 | up | STK19 | NM_004197 | down |
| TRAJ38 | ENST00000390499 | up | TGFBI | NM_000358 | down |

**Supplemental Table8** GO analysis: categories of AF-related gene in female

| **GO Name** | **p-value** | **-lgP** | **Gene** |
| --- | --- | --- | --- |
| small molecule  metabolic process | 4.72E-33 | 32.33 | FHL2/ECHS1/SLC25A6/MALL/  SLC25A20/PIP4K2B/NUP43/HEXA/  MED1/ABCG2/PI4K2A/EXT2/FMO2/  PSMB3/ACLY/PSAP/AASDHPPT/PDK1/  CYP1B1/SPR/SDHD/LCLAT1/SLC5A1/DLD/ACAD8/COX7C/GM2A/GALC/RAPGEF4/BCKDHB/PIK3R3/NUDT3/CP/HK2/  HIBCH/CNDP2/HADHA/TPMT/GNS/  HBA1/CH25H/ALAS2/HBB/PRELP/MAT2A/ARG1/CA1/CSPG4/DEGS2/ |
| immune response | 4.89E-31 | 30.31 | CXCL12/ENPP2/CCL13/CCL18/ETS1/  CEACAM8/CXCL2/PF4/IL8/THBS1/SUSD2/RGS1/IL6/C5AR1/CLEC4E/NFIL3/  IL1B/IL3/ |
| inflammatory response | 1.08E-25 | 24.97 | MECOM/CCL13/PDPN/CCL18/KLRG1/C  XCL2/CXCR3/IL8/THBS1/FOS/IL6/NFKBIZ/C5AR1/TNFAIP3/IL1B/  NLRP3/OLR1/ |
| blood coagulation | 3.89E-23 | 22.41 | VWF/JAM2/PRKCH/AKAP10/PSAP/  JAM3/TEK/GNA11/AKAP1/CRK/RAPGEF4/CD47/PF4/RHOB/HBB/THBS1/PPBP/PLAUR/TREM1/SERPINE1/CD177/TGFB2/OLR1/MAFF/P2RX1/KCNMB1/THBD/ |
| cell adhesion | 5.55E-23 | 22.26 | CXCL12/NRP1/VWF/NUAK1/TGFBI/  PCDHB3/POSTN/SVEP1/COL6A6/RELN/LAMB1/CD47/HES1/CXCR3/RHOB/THBS1/CLDN1/NEDD9/ |
| innate immune  response | 2.29E-20 | 19.64 | C6/CRK/KLRG1/FGF18/NR4A1/FOS/  TNFAIP3/TREM1/JUN/NLRP3/  APOBEC3A/SIGLEC15/HBEGF/ |
| angiogenesis | 6.63E-19 | 18.18 | ID1/NRP1/MED1/TGFBI/KDR/JAM3/  CYP1B1/TEK/ERAP1/ECSCR/FGF18/CXCR3/IL8/RHOB/JUN/SERPINE1/TGFB2/ZC3H12A/CSPG4/ |
| cellular protein  metabolic process | 2.88E-16 | 15.54 | SLC25A6/IGFBP3/SSR3/SRP9/MLEC/RP  L8/EEF1G/CMA1/RPS15/SPCS2/  GRPEL1/EEF2/PREB/RPL11/RPS16/  TOMM20/RPL38/ALG14/CP/RPS29/  GANAB/SRP14/ATF3/B3GNT5/IL8/PLAUR/TSPYL2/MUC21/ |
| positive regulation  of angiogenesis | 1.39E-15 | 14.86 | C6/KDR/CMA1/TEK/ERAP1/CTSH/  ETS1/RHOB/THBS1/IL1B/SERPINE1/GDF2/VASH2/ |
| neutrophil chemotaxis | 1.56E-14 | 13.81 | IL8/PPBP/C5AR1/IL1B/TGFB2/ |
| platelet activation | 1.69E-14 | 13.77 | VWF/PRKCH/PSAP/GNA11/CRK/  RAPGEF4/PF4/RHOB/THBS1/PPBP/IL6/SERPINE1/TGFB2/P2RX1/ |
| chemotaxis | 3.37E-14 | 13.47 | CXCL12/ENPP2/CCL13/ECSCR/CCL18/  CXCL2/CXCR3/PLAUR/C5AR1/FPR1/  PLP2/ |
| platelet degranulation | 8.31E-14 | 13.08 | VWF/PSAP/PF4/THBS1/PPBP/  SERPINE1/TGFB2/ |
| signal transduction | 4.82E-13 | 12.32 | CXCL12/KCNIP2/NRP1/EXT2/PRKCH/  AKAP10/SMOC2/CCL13/TEK/WWP1/  PDPN/GNA11/AKAP1/CDK4/CCL18/  PHB/ZDHHC13/FGF18/LIMD1/NR4A1/  IL8/PLAUR/NR4A2/RGS1/C5AR1/IL1B/  FPR1/NLRP3/P2RX1/NEDD9/  SFN/HBEGF/ |
| extracellular  matrix organization | 7.55E-12 | 11.12 | TGFBI/POSTN/CMA1/COL6A6/SMOC2/  ELN/NPNT/TGFB2/CTRB1/CTRB2/ |
| cellular response  to zinc ion | 1.04E-11 | 10.98 | MT2A/MT1A/MT1M/MT1X/MT1G/  MT1F/MT1H/MT1E/MT1B/ |
| cellular lipid  metabolic process | 1.10E-11 | 10.96 | FHL2/ECHS1/SLC25A20/MED1/ACLY/  LCLAT1/HADHA/ |
| negative regulation  of apoptotic process | 1.48E-11 | 10.83 | CXCL12/FHL2/MED1/KDR/BNIP3/  TXNDC5/EYA1/TEK/CTSH/CDKN1A/SOCS3/THBS1/BTG2/IER3/SCXA/IL6/TNFAIP3/IL1B/MYC/SERPINE1/OLR1/KRT18/PLK3/ |
| proteolysis | 6.79E-11 | 10.17 | PAMR1/CMA1/SPCS2/DLD/ERAP1/  PEPD/AMZ2/CP/RELN/CTSH/CNDP2/ADAMTS4/TNFAIP3/ADAMTS1/  OLR1/ADAMTS8/CTRB1/CTRB2/ |
| apoptotic process | 7.11E-11 | 10.15 | ID1/SLC25A6/IGFBP3/BNIP3/PSMB3/  MECOM/RRAGC/NMT1/GADD45B/CXCR3/RHOB/IER3/C5AR1/PRUNE2/  IL1B/CSRNP1/KRT18/ZC3H12A/  USP17L1P/SFN/PLK3/ |
| response to drug | 7.39E-11 | 10.13 | AK4/ABCG2/CDK4/SRP14/HADHA/JUNB/CDKN1A/THBS1/FOS/IL6/JUN/MYC/TGFB2/ARG1/WFDC1/ |
| positive regulation  of cell proliferation | 1.40E-10 | 9.85 | PGGT1B/KDR/RPS15/ALDH1A2/CDK4/  CTSH/ETS1/FGF18/CD47/ATF3/HES1/SCXA/IL6/MYC/TGFB2/IL3/ |
| muscle contraction | 1.85E-10 | 9.73 | KCNIP2/SCN7A/ACTA2/ |
| leukocyte migration | 2.16E-10 | 9.67 | JAM2/JAM3/TEK/CD47/TREM1/CD177/OLR1/THBD/ |
| cytokine-mediated  signaling pathway | 3.99E-10 | 9.40 | NUP43/MT2A/PF4/SOCS3/EGR1/IL6/  IL1B/PLP2/ |
| complement activation,  classical pathway | 5.10E-10 | 9.29 | C6/ |
| G-protein coupled receptor  signaling pathway | 6.21E-10 | 9.21 | CXCL12/APLNR/ATRNL1/ENPP2/  GNA11/RAPGEF4/PREX2/IL8/AREG/  RGS1/FPR1/OXER1/ |
| translation | 9.11E-10 | 9.04 | MRPS14/SSR3/SRP9/RPL8/MRPL33/  EEF1G/RPS15/SPCS2/EEF2/RPL11/RPS16/HARS2/RPL38/RPS29/SRP14/ |
| SRP-dependent cotranslational  protein targeting to membrane | 1.12E-09 | 8.95 | SSR3/SRP9/RPL8/RPS15/SPCS2/RPL11/  RPS16/RPL38/RPS29/SRP14/ |
| gene expression | 1.30E-09 | 8.88 | NUDT21/SSR3/DDX23/PRPF4/MED1/  SRP9/RPL8/PSMB3/EEF1G/HNRNPH2/KARS/PARP1/METTL3/RPS15/SPCS2/EEF2/RPL11/RPS16/AARS/HNRNPA3/  HARS2/RPL38/RPS29/CDC40/SRP14/  NR4A1/ZFP36/JUNB/PF4/NR4A2/NR4A3/JUN/CCNH/MYC/ |
| vasculogenesis | 1.32E-09 | 8.88 | KDR/ZFP36/JUNB/JUN/HEY2/TIPARP/  GDF2/ |
| negative regulation  of cell proliferation | 1.60E-09 | 8.80 | SMYD2/IGFBP3/ALDH1A2/PHB/ETS1/  CDKN1A/IL8/BTG2/IL6/IL1B/  ADAMTS1/JUN/KLF4/  TGFB2/ADAMTS8/AXIN2/SFN/ |
| muscle filament sliding | 1.93E-09 | 8.71 | MYL3/ |
| negative regulation  of growth | 1.95E-09 | 8.71 | MT2A/MT1A/MT1M/MT1X/MT1G/  MT1F/MT1H/MT1E/MT1B/ |
| interferon-gamma-mediated  signaling pathway | 2.11E-09 | 8.68 | MT2A/SOCS3/ |
| positive regulation of  transcription from RNA  polymerase II promoter | 4.00E-09 | 8.40 | MED1/HMGN3/ARMCX3/MECOM/  EYA1/ETS1/THRAP3/KLF2/HES1/JUNB/  EGR1/JUND/SCXA/NR4A2/FOS/IL6/  IL1B/JUN/CCNH/CSRNP1/MYC/KLF4/  SERPINE1/HEY2/HEYL/FOXQ1/  GDF2/IL3/BMP3/BMP3/ |
| response to mechanical stimulus | 7.03E-09 | 8.15 | ETS1/JUNB/BTG2/JUND/FOS/IL6/JUN/  SOST/ |
| carbohydrate metabolic process | 9.93E-09 | 8.00 | NUP43/HEXA/EXT2/SLC5A1/GALC/  HK2/GANAB/GNS/PRELP/CSPG4/ |
| tricarboxylic acid cycle | 1.86E-08 | 7.73 | DHTKD1/SDHD/ACO1/DLD/ |
| positive regulation  of cell migration | 3.01E-08 | 7.52 | KDR/PDPN/CTSH/LAMB1/THBS1/  HBEGF/ |
| response to lipopolysaccharide | 3.01E-08 | 7.52 | JUND/FOS/IL6/C5AR1/IL1B/JUN/ |
| response to hypoxia | 3.24E-08 | 7.49 | BNIP3/ETS1/LIMD1/ALAS2/THBS1/  NR4A2/TGFB2/ |
| angiotensin maturation | 3.75E-08 | 7.43 | CMA1/CP/ |
| translational elongation | 5.38E-08 | 7.27 | RPL8/EEF1G/RPS15/EEF2/RPL11/  RPS16/RPL38/RPS29/ |
| branched-chain amino  acid catabolic process | 5.55E-08 | 7.26 | DLD/ACAD8/BCKDHB/HIBADH/  HIBCH/ |
| organ regeneration | 6.56E-08 | 7.18 | MED1/CDK4/CDKN1A/NR4A3/C5AR1/ |
| cell-cell signaling | 9.18E-08 | 7.04 | NRP1/CCL13/TEK/NUDT3/CCL18/  FGF18/AREG/IL1B/TGFB2/IL3/BMP3/ |
| response to  corticosterone stimulus | 1.51E-07 | 6.82 | CALM3/JUNB/CDKN1A/FOS/JUN/ |
| transcription,  NA-dependent | 1.72E-07 | 6.76 | SMYD2/ATP1B4/ID1/FHL2/ZNF224/  ZNF223/ZNF546/MED28/ZNF284/PHB2/  CHURC1/ZNF658B/ZNF436/ZBTB6/  LBH/TCEAL8/ZNF426/ZNF75A/ZIK1/  PARP1/EBF2/EBF3/ZNF552/MECOM/ZFP14/PREB/RRAGC/EYA1/PER3/ACAD8/HIF1AN/TRIM27/PNRC2/HMG20A/  SLIRP/PHB/ETS1/ZNF134/ZNF22/  LIMD1/ZNF420/ATF3/KLF2/JUNB/PER1/FOSL2/SCXA/NR4A2/FOS/ |
| glycolysis | 1.88E-07 | 6.73 | DHTKD1/PGAM4/HK2/ |
| complement  activation | 1.93E-07 | 6.71 | C6/ |
| response to virus | 2.04E-07 | 6.69 | CXCL12/EEF1G/RPS15/ACTA2/ |
| viral infectious  cycle | 3.05E-07 | 6.52 | SLC25A6/RPL8/RPS15/RPL11/  RPS16/RPL38/RPS29/ |
| viral transcription | 4.36E-07 | 6.36 | RPL8/RPS15/RPL11/RPS16/RPL38/  RPS29/ |
| response to hydrogen  peroxide | 4.45E-07 | 6.35 | HBA1/HBB/DUSP1/JUN/OLR1/ |
| collagen fibril  organization | 4.45E-07 | 6.35 | P4HA1/SCXA/TGFB2/ |
| positive regulation of  apoptotic process | 4.96E-07 | 6.30 | IGFBP3/BNIP3/ALDH1A2/CDK4/RHOB/  IER3/DUSP1/TGFB2/ |
| translational termination | 5.94E-07 | 6.23 | RPL8/RPS15/RPL11/RPS16/RPL38/  RPS29/ |
| cellular component movement | 6.23E-07 | 6.21 | ENPP2/PDPN/CXCR3/IL8/PLAUR/FPR1/ |
| cellular nitrogen  compound metabolic process | 7.02E-07 | 6.15 | PSMB3/DLD/ACAD8/BCKDHB/  HIBADH/HIBCH/CNDP2/MAT2A/ARG1/ |
| negative regulation of  transcription from RNA  polymerase II promoter | 7.64E-07 | 6.12 | SMYD2/ID1/FHL2/MED1/PARP1/PER3/  TRIM27/HMG20A/PHB/HES1/ZFP36/  PER1/EGR1/SIK1/FOSB/FOS/NFIL3/  MYC/KLF4/HEY2/BHLHE40/HBZ/PLK3/IL3/ |
| respiratory electron  transport chain | 8.02E-07 | 6.10 | SDHD/COX7C/CP/ |
| positive regulation of  endothelial cell proliferation | 9.18E-07 | 6.04 | NRP1/KDR/TEK/NR4A1/JUN/ARG1/  GDF2/VASH2/ |
| transmembrane transport | 9.48E-07 | 6.02 | C6/SLC25A6/ATP6V0E1/SLC33A1/  NUP43/ABCG2/SLC2A12/SLC4A4/  SLC5A1/SLC16A7/WWP1/CLCN3/  SLC35B4/SLC31A1/CP/SLC39A7/HK2/  SLC39A8/ |
| positive regulation of  transcription, DNA-dependent | 9.76E-07 | 6.01 | FHL2/CHURC1/TAF15/MED1/NIF3L1/  LBH/EBF2/EBF3/MECOM/PHB/ETS1/  THRAP3/KLF2/EGR1/SCXA/FOS/IL6/IL1B/JUN/MYC/KLF4/GDF2/CREB5/SOST/ |
| vascular endothelial growth  factor receptor signaling pathway | 1.13E-06 | 5.95 | NRP1/KDR/ |
| circadian rhythm | 1.20E-06 | 5.92 | PER3/CDK4/PER1/EGR1/JUND/NFIL3/  JUN/IL3/ |
| cellular response to vascular  endothelial growth factor stimulus | 1.28E-06 | 5.89 | KDR/NR4A1/MT1G/ |
| keratan sulfate catabolic process | 1.38E-06 | 5.86 | HEXA/GNS/PRELP/ |
| aging | 2.45E-06 | 5.61 | JUND/FOS/IL6/JUN/ |
| regulation of transcription,  DNA-dependent | 2.78E-06 | 5.56 | ATP1B4/ZNF224/ZNF223/ZNF546/  MED28/RNF141/ZNF284/CARHSP1/ZNF658B/ZNF436/ZBTB6/TCEAL8/ZNF426/ZNF75A/ZIK1/PARP1/EBF2/ZNF146/  EBF3/ZNF552/MECOM/ZFP14/PREB/  ACAD8/PNRC2/HMG20A/ZNF350/  SLIRP/PHB/ZNF134/ZNF22/LIMD1/  ZNF420/ZFP36/CCNH/TSPYL2/  BHLHE40/PADI4/ |
| response to cAMP | 3.73E-06 | 5.43 | BCKDHB/JUNB/FOS/DUSP1/JUN/  CAMP/ |
| transport | 3.97E-06 | 5.40 | ATP1B4/C6/SLC25A20/SLC33A1/  ABCG2/  TNPO3/SLC4A4/GRIK2/CLCN3/CA1/  P2RX1/ |
| cell surface receptor  signaling pathway | 4.09E-06 | 5.39 | PIP4K2B/KLRG1/ |
| cellular response to  cadmium ion | 4.26E-06 | 5.37 | MT1A/MT1X/MT1G/MT1F/MT1H/  MT1E/ |
| skeletal system development | 4.31E-06 | 5.37 | NPR3/HEXA/POSTN/GNA11/ADAMTS4/PRELP/BMP3/ |
| positive regulation of  cell-substrate adhesion | 5.32E-06 | 5.27 | ID1/SMOC2/THBS1/NPNT/ |
| positive regulation of  ERK1 and ERK2 cascade | 5.62E-06 | 5.25 | KDR/TEK/FGF18/IL6/C5AR1/NPNT/ |
| negative regulation of  cysteine-type endopeptidase  activity involved in  apoptotic process | 5.82E-06 | 5.24 | NR4A1/THBS1/IL6/TNFAIP3/KLF4/SFN/ |
| muscle organ development | 6.41E-06 | 5.19 | HBEGF/ |
| negative regulation of  endothelial cell apoptotic  process | 6.46E-06 | 5.19 | KDR/TEK/TNFAIP3/ |
| chronic inflammatory  response | 6.46E-06 | 5.19 | THBS1/ |
| positive regulation  of endothelial cell migration | 6.98E-06 | 5.16 | NRP1/KDR/TEK/ETS1/THBS1/ |
| nuclear-transcribed  mRNA catabolic process,  nonsense-mediated decay | 7.94E-06 | 5.10 | RPL8/RPS15/RPL11/RPS16/PNRC2/  RPL38/RPS29/ |
| translational initiation | 7.94E-06 | 5.10 | RPL8/RPS15/RPL11/RPS16/RPL38/  RPS29/ |
| negative regulation of  leukocyte apoptotic process | 8.97E-06 | 5.05 | CXCL12/ |
| response to wounding | 9.19E-06 | 5.04 | NRP1/VWF/IL6/TGFB2/ |
| negative regulation of  angiogenesis | 9.19E-06 | 5.04 | TEK/PF4/THBS1/GDF2/ |
| positive regulation of  neutrophil chemotaxis | 1.08E-05 | 4.97 | IL8/ |
| response to organic  cyclic compound | 1.12E-05 | 4.95 | BTG2/JUND/JUN/ |
| positive regulation of  peptidyl-tyrosine phosphorylation | 1.28E-05 | 4.89 | NRP1/EHD4/RELN/IL6/CSPG4/IL3/ |
| cell proliferation | 1.55E-05 | 4.81 | TGFBI/VTI1B/PDK1/PDPN/TRIM27/  ZFP36/  AREG/ELN/TGFB2/AXIN2/CSPG4/ |
| response to  progesterone stimulus | 1.60E-05 | 4.80 | JUNB/THBS1/FOS/JUN/TGFB2/ |
| platelet-derived growth  factor receptor signaling pathway | 1.60E-05 | 4.80 | NRP1/CSRNP1/TIPARP/ |
| lipid storage | 1.62E-05 | 4.79 | HEXA/GM2A/ |
| positive regulation of  NF-kappaB import into nucleus | 1.62E-05 | 4.79 | IL1B/ |
| mRNA metabolic process | 1.65E-05 | 4.78 | RPL8/PSMB3/RPS15/RPL11/RPS16/  RPL38/RPS29/ZFP36/ |
| positive regulation of  inflammatory response | 1.67E-05 | 4.78 | CD47/SERPINE1/IL3/ |
| positive regulation of  osteoblast differentiation | 1.82E-05 | 4.74 | JUND/IL6/JUN/NPNT/GDF2/ |
| regulation of cell cycle | 1.98E-05 | 4.70 | MED1/MECOM/CDK4/JUNB/GADD45B/JUN/ |
| positive regulation of  phosphatidylinositol 3-kinase cascade | 2.02E-05 | 4.70 | KDR/TEK/RELN/TGFB2/ |
| positive regulation of  I-kappaB kinase/NF-kappaB cascade | 2.03E-05 | 4.69 | MUL1/WLS/ZDHHC13/IL1B/ |
| response to amino  acid stimulus | 2.13E-05 | 4.67 | ATP6V0E1/AARS/IL6/ARG1/ |
| viral reproduction | 2.14E-05 | 4.67 | SLC25A6/NUP43/RPL8/PSMB3/RPS15/  RPL11/RPS16/RPL38/NMT1/RPS29/  CCNH/ |
| chondrocyte development | 2.25E-05 | 4.65 | IMPAD1/FGF18/ |
| liver development | 2.27E-05 | 4.64 | VWF/AK4/MED1/ALDH1A2/HES1/JUN/ARG1/ |
| negative regulation  of protein kinase activity | 2.50E-05 | 4.60 | TRIM27/GADD45B/SOCS3/IL6/SFN/ |
| leukocyte migration  involved in inflammatory response | 2.62E-05 | 4.58 | JAM3/ |
| cell-cell adhesion | 2.77E-05 | 4.56 | CD93/JAM2/TEK/PDPN/ |
| cellular response to drug | 2.90E-05 | 4.54 | MT2A/EGR1/IL1B/MYC/ |
| embryo implantation | 2.90E-05 | 4.54 | LAMB1/IL1B/ |
| regulation of blood pressure | 2.92E-05 | 4.53 | NPR3/ERAP1/HBB/ACTA2/P2RX1/ |
| protein targeting to  mitochondrion | 3.46E-05 | 4.46 | SLC25A6/GRPEL1/MTX3/TOMM20/ |
| regulation of acetyl-CoA  biosynthetic process from pyruvate | 3.75E-05 | 4.43 | PDK1/DLD/ |
| elevation of cytosolic  calcium ion concentration | 4.13E-05 | 4.38 | CXCR3/C5AR1/ |
| fat cell differentiation | 4.85E-05 | 4.31 | MED1/ERAP1/KLF4/CREB5/ |
| response to ethanol | 5.01E-05 | 4.30 | EGR1/ |
| RNA metabolic process | 5.04E-05 | 4.30 | RPL8/PSMB3/RPS15/RPL11/RPS16/  RPL38/RPS29/ZFP36/ |
| transmembrane receptor  protein tyrosine kinase  signaling pathway | 5.25E-05 | 4.28 | KDR/TEK/CSPG4/ |
| positive regulation  of protein kinase activity | 5.31E-05 | 4.27 | RELN/AXIN2/ |
| cellular response  to hydrogen peroxide | 5.90E-05 | 4.23 | BNIP3/ETS1/KLF2/RHOB/IL6/  TNFAIP3/ARG1/ |
| cellular response  to calcium ion | 5.90E-05 | 4.23 | JUNB/JUND/FOSB/FOS/JUN/ |
| positive regulation  of nitric oxide biosynthetic process | 5.90E-05 | 4.23 | HBB/IL6/IL1B/ |
| valine catabolic process | 5.95E-05 | 4.23 | ACAD8/HIBADH/HIBCH/ |
| Tie signaling pathway | 5.95E-05 | 4.23 | TEK/ |
| glomerulus vasculature development | 5.95E-05 | 4.23 | HES1/ |
| cellular iron ion homeostasis | 6.03E-05 | 4.22 | ATP6V0E1/ABCG2/ACO1/CP/ALAS2/  MYC/GDF2/ |
| extracellular matrix disassembly | 6.40E-05 | 4.19 | CMA1/COL6A6/CTRB1/CTRB2/ |
| cellular response to hypoxia | 6.40E-05 | 4.19 | BNIP3/HIF1AN/EGR1/ |
| triglyceride biosynthetic process | 6.42E-05 | 4.19 | ACLY/LCLAT1/ |
| positive regulation of  NF-kappaB transcription factor activity | 6.85E-05 | 4.16 | PRKCH/IL6/IL1B/NLRP3/ |
| cell differentiation | 6.86E-05 | 4.16 | EXT2/MECOM/ECSCR/GADD45B/  HEMGN/GGN/SCXA/NPNT/ZC3H12A/  TSSK3/BMP3/ |
| negative regulation of  canonical Wnt receptor  signaling pathway | 7.22E-05 | 4.14 | LIMD1/EGR1/AXIN2/SOST/ |
| regulation of apoptotic process | 7.69E-05 | 4.11 | PSMB3/PHB/IL6/DUSP1/ |
| keratinocyte differentiation | 7.80E-05 | 4.11 | MED1/SPR/SPRR1B/SFN/ |
| cell communication | 8.12E-05 | 4.09 | FREM1/SNX12/CCL18/GJD2/ |
| negative regulation of  transcription, DNA-dependent | 8.40E-05 | 4.08 | ID1/FHL2/ZNF224/PHB2/MECOM/  WWP1/ZNF350/PHB/ZNF22/LIMD1/  ATF3/HES1/PER1/JUN/KLF4/HEY2/  HEYL/ |
| peptide cross-linking | 8.55E-05 | 4.07 | SPR/THBS1/SPRR1B/ |
| response to light stimulus | 9.01E-05 | 4.05 | JUNB/PER1/JUND/FOS/DUSP1/JUN/ |
| endoplasmic reticulum  unfolded protein response | 1.02E-04 | 3.99 | PREB/AARS/ATF3/IL8/TSPYL2/ |
| cellular response to  lipopolysaccharide | 1.02E-04 | 3.99 | IL8/TNFAIP3/NLRP3/SERPINE1/ARG1/  ZC3H12A/ |
| cellular response to cAMP | 1.13E-04 | 3.95 | EGR1/CAMP/ |
| positive regulation  of interleukin-6 production | 1.14E-04 | 3.94 | IL6/IL1B/IL3/ |
| regulation of heart  contraction | 1.14E-04 | 3.94 | KCNIP2/HBEGF/ |
| positive regulation  of gene expression | 1.19E-04 | 3.92 | MED1/ALDH1A2/CTSH/PF4/SCXA/  NFIL3/TGFB2/ZC3H12A/IL3/ |
| cellular response to  mechanical stimulus | 1.21E-04 | 3.92 | BNIP3/EGR1/IL1B/ |
| positive regulation of  T cell proliferation | 1.30E-04 | 3.89 | HES1/IL6/IL1B/ |
| response to glucose stimulus | 1.40E-04 | 3.85 | THBS1/EGR1/ |
| learning or memory | 1.40E-04 | 3.85 | GM2A/EGR1/AFF2/GALR3/ |
| patterning of blood vessels | 1.40E-04 | 3.85 | CXCL12/NRP1/GDF2/ |
| T cell receptor  signaling pathway | 1.42E-04 | 3.85 | STOML2/VASP/ |
| negative regulation  of cell growth | 1.44E-04 | 3.84 | MUL1/PHB/CDKN1A/TGFB2/TSPYL2/  WFDC1/GDF2/ |
| G1/S transition of  mitotic cell cycle | 1.53E-04 | 3.81 | PSMB3/CDK4/CDKN1A/CCNH/CDCA5/PLK3/ |
| regulation of  inflammatory response | 1.70E-04 | 3.77 | CMA1/ |
| potassium ion  transport | 1.76E-04 | 3.75 | ATP1B4/KCNIP2/HCN1/KCNMB1/ |
| regulation of action  potential in neuron | 1.87E-04 | 3.73 | GRIK2/P2RX1/ |
| regulation of the  force of heart contraction | 1.87E-04 | 3.73 | MYL3/ |
| positive regulation  of phosphatidylinositol  3-kinase activity | 1.87E-04 | 3.73 | TEK/ |
| regulation of  immune response | 1.95E-04 | 3.71 | KLRG1/ |
| transforming growth  factor beta receptor  signaling pathway | 2.04E-04 | 3.69 | ID1/PARP1/JUNB/FOS/JUN/MYC/  SERPINE1/TGFB2/ |
| positive regulation  of cell adhesion mediated  by integrin | 2.05E-04 | 3.69 | TGFB2/ |
| glucose metabolic process | 2.06E-04 | 3.69 | PDK1/ |
| response to estradiol stimulus | 2.10E-04 | 3.68 | ALDH1A2/ETS1/DUSP1/WFDC1/ |
| brain development | 2.25E-04 | 3.65 | CXCL12/UTP3/AK4/MED1/RELN/AFF2/ |
| regulation of cell shape | 2.26E-04 | 3.65 | KDR/CCL13/PDPN/LIMD1/IL6/ |
| endothelial cell chemotaxis | 2.32E-04 | 3.63 | NRP1/NR4A1/ |
| negative regulation  of calcium ion import | 2.32E-04 | 3.63 | SLN/TRIM27/ |
| vascular smooth muscle  cell development | 2.32E-04 | 3.63 | HES1/HEY2/ |
| substrate adhesion-dependent  cell spreading | 2.37E-04 | 3.62 | SPR/TEK/LAMB1/ |
| outflow tract morphogenesis | 2.47E-04 | 3.61 | EYA1/HES1/THBS1/JUN/HEY2/HEYL/ |
| glycosaminoglycan  metabolic process | 2.71E-04 | 3.57 | HEXA/EXT2/GNS/PRELP/CSPG4/ |
| response to calcium ion | 2.82E-04 | 3.55 | THBS1/IL6/DUSP1/KCNMB1/ |
| anatomical structure morphogenesis | 2.91E-04 | 3.54 | EYA1/FGF18/IER3/KRT18/ |
| mitochondrial electron  transport, NADH to ubiquinone | 2.95E-04 | 3.53 | DLD/ |
| wound healing | 3.06E-04 | 3.51 | SPR/TGFB2/ |
| heart development | 3.07E-04 | 3.51 | ID1/NRP1/TEK/GNA11/TGFB2/ |
| regulation of cell migration | 3.25E-04 | 3.49 | ENPP2/ |
| antigen processing and  presentation of exogenous  peptide antigen via MHC class I | 3.28E-04 | 3.48 | PSMB3/ |
| defense response to virus | 3.30E-04 | 3.48 | BNIP3/IL6/NLRP3/APOBEC3A/ |
| positive regulation of  monocyte chemotaxis | 3.33E-04 | 3.48 | CXCL12/SERPINE1/ |
| virus-host interaction | 3.42E-04 | 3.47 | CD93/SLC25A6/KDR/BNIP3/PSMB3/  KARS/CLDN1/KRT18/ |
| defense response to  Gram-negative bacterium | 3.48E-04 | 3.46 | IL6/SERPINE1/CAMP/ |
| termination of G-protein  coupled receptor signaling pathway | 3.50E-04 | 3.46 | AKAP10/AKAP1/RGS1/RGS2/RGS16/  AXIN2/ |
| response to hormone stimulus | 3.50E-04 | 3.46 | FHL2/ |
| negative regulation  of neuron apoptotic process | 3.53E-04 | 3.45 | GRIK2/AARS/NR4A2/NR4A3/C5AR1/  JUN/ |
| cell cycle arrest | 3.61E-04 | 3.44 | CDKN1A/IL8/THBS1/MYC/TGFB2/ |
| post-embryonic development | 3.65E-04 | 3.44 | MECOM/IMPAD1/ACO1/NR4A2/  CSRNP1/TIPARP/ |
| positive regulation  of cell cycle | 3.68E-04 | 3.43 | PGGT1B/RPS15/NR4A3/TGFB2/ |
| receptor internalization | 3.68E-04 | 3.43 | IL8/ |
| induction of apoptosis | 3.80E-04 | 3.42 | BNIP3/ETS1/NR4A1/THBS1/NLRP3/  TGFB2/ |
| odontogenesis | 4.52E-04 | 3.34 | LAMB1/ZNF22/TGFB2/AXIN2/ |
| cellular response to  fibroblast growth factor stimulus | 4.60E-04 | 3.34 | NR4A1/IL8/ |
| positive regulation  of erythrocyte differentiation | 4.60E-04 | 3.34 | ETS1/ |
| positive regulation  of protein kinase B signaling cascade | 4.91E-04 | 3.31 | TEK/THBS1/IL6/C1QTNF1/HBEGF/ |
| positive regulation  of cell growth | 4.91E-04 | 3.31 | TGFB2/SFN/HBEGF/ |
| regulation of glucose  metabolic process | 5.10E-04 | 3.29 | IGFBP3/PDK1/C1QTNF1/ |
| in utero embryonic  development | 5.18E-04 | 3.29 | C6/MECOM/NMT1/KLF2/MAFF/ |
| response to toxic  substance | 5.49E-04 | 3.26 | CYP1B1/CDK4/CDKN1A/FOS/ |
| brown fat cell  differentiation | 5.50E-04 | 3.26 | BNIP3/CP/RGS2/ |
| face morphogenesis | 5.50E-04 | 3.26 | CSRNP1/TGFB2/TIPARP/ |
| cellular response  to interleukin-1 | 5.50E-04 | 3.26 | KLF2/IL8/ |
| negative regulation  of cell cycle | 5.66E-04 | 3.25 | ETS1/RHOB/TSPYL2/ |
| calcium-mediated signaling | 5.66E-04 | 3.25 | CXCR3/IL8/ |
| negative regulation  of cell adhesion mediated  by integrin | 5.67E-04 | 3.25 | SERPINE1/ |
| positive regulation  of keratinocyte migration | 5.67E-04 | 3.25 | HBEGF/ |
| sprouting angiogenesis | 5.97E-04 | 3.22 | SPR/TEK/THBS1/ |
| positive regulation of  interleukin-8 production | 5.97E-04 | 3.22 | IL1B/SERPINE1/GDF2/ |
| protein peptidyl-prolyl  isomerization | 5.97E-04 | 3.22 | FKBP2/FKBPL/ |
| cytolysis | 5.97E-04 | 3.22 | C6/ |
| response to oxidative stress | 6.07E-04 | 3.22 | DUSP1/ |
| osteoblast differentiation | 6.33E-04 | 3.20 | FHL2/JUNB/JUN/GDF2/ |
| defense response to bacterium | 6.61E-04 | 3.18 | PPBP/CAMP/ |
| positive regulation of  protein phosphorylation | 6.64E-04 | 3.18 | KDR/TEK/IL1B/AXIN2/ |
| negative regulation of  cell adhesion | 6.65E-04 | 3.18 | JAM2/TGFBI/LAMB1/ |
| ribosomal small subunit  biogenesis | 7.46E-04 | 3.13 | MALL/RPS15/RPS16/ |
| regulation of mRNA  stability | 7.46E-04 | 3.13 | CARHSP1/ZFP36/ |
| regulation of innate  immune response | 7.46E-04 | 3.13 | ERAP1/ |
| positive regulation  of nitric-oxide synthase  biosynthetic process | 7.46E-04 | 3.13 | KDR/ |
| xenobiotic metabolic process | 7.59E-04 | 3.12 | FMO2/UGT2B4/CYP1B1/CNDP2/TPMT/MAT2A/ |
| blood circulation | 7.61E-04 | 3.12 | CXCL12/ELN/OLR1/ |
| keratan sulfate  metabolic process | 7.97E-04 | 3.10 | HEXA/GNS/PRELP/ |
| positive regulation of  tumor necrosis factor production | 7.97E-04 | 3.10 | ARFGEF2/PF4/ |
| cellular response to  tumor necrosis factor | 8.78E-04 | 3.06 | KLF2/IL8/NPNT/ |
| integrin-mediated signaling pathway | 9.33E-04 | 3.03 | CD47/ADAMTS1/NEDD9/ |
| positive regulation  of epithelial to  mesenchymal transition | 9.64E-04 | 3.02 | TGFB2/AXIN2/ |
| cell-matrix adhesion | 1.03E-03 | 2.99 | ID1/FREM1/JAM3/TEK/NPNT/ |
| response to hyperoxia | 1.05E-03 | 2.98 | BNIP3/PDPN/CDK4/CDKN1A/ |
| S phase of mitotic  cell cycle | 1.06E-03 | 2.98 | PSMB3/CDK4/CDKN1A/CCNH/CDCA5/PLK3/ |
| intramembranous  ossification | 1.11E-03 | 2.96 | FGF18/AXIN2/ |
| cellular response  to hepatocyte growth  factor stimulus | 1.11E-03 | 2.96 | NRP1/MED1/ |
| toll-like receptor  signaling pathway | 1.17E-03 | 2.93 | FOS/JUN/ |
| cellular response  to organic substance | 1.20E-03 | 2.92 | PSAP/NR4A1/IL1B/ |
| positive regulation  of B cell proliferation | 1.32E-03 | 2.88 | CDKN1A/ |
| cardiac muscle  contraction | 1.32E-03 | 2.88 | MYL3/ |
| cell migration involved  in sprouting angiogenesis | 1.44E-03 | 2.84 | KDR/SPR/NR4A1/ |
| positive regulation  of macrophage derived  foam cell differentiation | 1.44E-03 | 2.84 | PRKCH/PF4/ |
| peptidyl-proline  modification | 1.44E-03 | 2.84 | FKBP2/FKBPL/ |
| response to  osmotic stress | 1.44E-03 | 2.84 | PLK3/ |
| insulin receptor  signaling pathway | 1.48E-03 | 2.83 | ATP6V0E1/CRK/PIK3R3/FGF18/ |
| fatty acid  biosynthetic process | 1.49E-03 | 2.83 | AASDHPPT/CH25H/DEGS2/ |
| response to stress | 1.51E-03 | 2.82 | ATF3/ZFP36/GADD45B/ |
| activation of  signaling protein  activity involved in  unfolded protein response | 1.59E-03 | 2.80 | PREB/ATF3/IL8/TSPYL2/ |
| response to insulin  stimulus | 1.69E-03 | 2.77 | HADHA/IL6/ |
| arachidonic acid  metabolic process | 1.69E-03 | 2.77 | CYP1B1/ |
| antigen processing  and presentation of  peptide antigen via  MHC class I | 1.74E-03 | 2.76 | PSMB3/ERAP1/ |
| multicellular  organismal development | 1.79E-03 | 2.75 | CHURC1/LBH/ANGPTL2/EBF2/EBF3/  LCLAT1/EYA1/LIMD1/GADD45B/  HEMGN/GGN/FOSB/FOS/IFRD1/AXIN2/TSSK3/VENTX/ |
| acute-phase response | 1.79E-03 | 2.75 | IL6/ |
| pyruvate metabolic  process | 1.80E-03 | 2.74 | PDK1/DLD/ |
| regulation of calcium  ion transport | 1.80E-03 | 2.74 | SLN/P2RX1/ |
| protein glycosylation | 1.88E-03 | 2.73 | EXT2/B3GALT2/B3GNT5/  ST6GALNAC5/ |
| protein folding | 1.89E-03 | 2.72 | MLEC/GRPEL1/FKBP2/AARS/GANAB/ |
| toxin metabolic process | 1.90E-03 | 2.72 | FMO2/CYP1B1/ |
| cellular response  to steroid hormone  stimulus | 1.90E-03 | 2.72 | MED1/EGR1/ |
| ribosomal small  subunit assembly | 1.90E-03 | 2.72 | MALL/ |
| positive regulation  of macrophage chemotaxis | 1.90E-03 | 2.72 | THBS1/ |
| positive regulation  of intracellular estrogen  receptor signaling pathway | 1.90E-03 | 2.72 | MED1/ |
| relaxation of cardiac muscle | 1.90E-03 | 2.72 | RGS2/ |
| tRNA processing | 1.91E-03 | 2.72 | KARS/AARS/TRIT1/TRUB1/ |
| positive regulation  of peptidyl-serine  phosphorylation | 1.91E-03 | 2.72 | TEK/IL6/ |
| heart trabecula formation | 1.91E-03 | 2.72 | FHL2/TEK/ADAMTS1/HEY2/ |
| response to ATP | 1.91E-03 | 2.72 | IL1B/P2RX1/ |
| monocyte chemotaxis | 1.91E-03 | 2.72 | IL6/ |
| glycolipid catabolic process | 1.92E-03 | 2.72 | NAGA/GM2A/ |
| cellular response  to erythropoietin | 1.92E-03 | 2.72 | MT2A/MT1X/ |
| negative regulation  of adaptive immune response | 1.92E-03 | 2.72 | TRIM27/ |
| atrioventricular  valve development | 1.92E-03 | 2.72 | HEY2/ |
| negative regulation  of osteoclast proliferation | 1.92E-03 | 2.72 | TNFAIP3/ |
| negative regulation  of calcium ion binding | 1.92E-03 | 2.72 | SLN/ |
| response to  glucocorticoid stimulus | 1.95E-03 | 2.71 | BCKDHB/IL6/DUSP1/ |
| defense response | 1.95E-03 | 2.71 | NLRP3/ |
| fatty acid beta-oxidation | 2.07E-03 | 2.68 | ECHS1/EHHADH/HADHA/ |
| transcription from  RNA polymerase II promoter | 2.08E-03 | 2.68 | NUDT21/PARP1/ETS1/CDC40/EGR1/  JUND/FOS/NFIL3/JUN/CCNH/KLF4/  CEBPD/BHLHE40/MAFF/CREB5/IL3/ |
| response to cytokine stimulus | 2.14E-03 | 2.67 | PGGT1B/ALDH1A2/JUNB/FOS/JUN/ |
| negative regulation  of inflammatory response | 2.14E-03 | 2.67 | TEK/IER3/TNFAIP3/NLRP3/KLF4/ |
| cell maturation | 2.39E-03 | 2.62 | KDR/HES1/ |
| cell migration | 2.43E-03 | 2.61 | LAMB1/LIMD1/HES1/THBS1/TGFB2/ |
| cellular response to  extracellular stimulus | 2.48E-03 | 2.60 | CDKN1A/NR4A2/FOS/ |
| blood vessel morphogenesis | 2.48E-03 | 2.60 | ID1/THBS1/GDF2/ |
| negative regulation of  growth of symbiont in host | 2.48E-03 | 2.60 | CAMP/ |
| positive regulation of  reactive oxygen species metabolic process | 2.60E-03 | 2.59 | CDKN1A/THBS1/ZC3H12A/ |
| leukocyte cell-cell adhesion | 2.60E-03 | 2.59 | OLR1/ |
| ventricular cardiac  muscle tissue morphogenesis | 2.60E-03 | 2.59 | MYL3/ |
| cellular calcium ion  homeostasis | 2.62E-03 | 2.58 | CXCL12/GRIK2/STOML2/CCL13/ |
| response to peptide  hormone stimulus | 2.73E-03 | 2.56 | JUNB/BTG2/JUND/NR4A3/JUN/ |
| folic acid transport | 2.96E-03 | 2.53 | FOLR2/PDPN/ |
| oxidation-reduction  process | 2.97E-03 | 2.53 | CYP1B1/SPR/HIF1AN/ |
| peptidyl-tyrosine  phosphorylation | 3.00E-03 | 2.52 | KDR/TEK/RELN/ |
| mesoderm formation | 3.08E-03 | 2.51 | EXT2/WLS/SCXA/NR4A3/ |
| positive regulation  of fat cell differentiation | 3.08E-03 | 2.51 | ZC3H12A/ |
| positive regulation  of fibroblast proliferation | 3.12E-03 | 2.51 | CDK4/CDKN1A/FOSL2/FOS/JUN/MYC/ |
| positive regulation  of smooth muscle cell  proliferation | 3.12E-03 | 2.51 | IL6/JUN/HBEGF/ |
| humoral immune  response | 3.12E-03 | 2.51 | IL6/TREM1/ |
| ossification | 3.15E-03 | 2.50 | EXT2/RPL38/GDF2/SOST/BMP3/ |
| response to bacterium | 3.17E-03 | 2.50 | MECOM/ERAP1/CD47/ |
| decidualization | 3.17E-03 | 2.50 | JUNB/JUN/ |
| long-chain fatty  acid metabolic process | 3.17E-03 | 2.50 | ACOT2/ |
| adipose tissue  development | 3.17E-03 | 2.50 | CREB5/ |
| intracellular  signal transduction | 3.32E-03 | 2.48 | CARHSP1/PLCL2/PRKCH/PLCL1/  ARFGEF2/PREX2/IL8/TREM1/DUSP1/  SHC4/CSPG4/ |
| collagen catabolic  process | 3.45E-03 | 2.46 | COL6A6/PEPD/ |
| cellular protein  localization | 3.54E-03 | 2.45 | ARMCX3/RRAGC/EYA1/AXIN2/ |
| tRNA aminoacylation  for protein translation | 3.54E-03 | 2.45 | KARS/AARS/HARS2/ |
| regulation of G-protein  coupled receptor  protein signaling pathway | 3.54E-03 | 2.45 | RGS1/RGS2/RGS16/ |
| cellular response  to stress | 3.54E-03 | 2.45 | HIF1AN/NR4A2/NR4A3/ |
| intracellular receptor  signaling pathway | 3.63E-03 | 2.44 | NR4A1/NR4A2/ |
| cell redox homeostasis | 3.71E-03 | 2.43 | TXNDC11/TXNDC5/DLD/IL6/ |
| lipid transport | 3.71E-03 | 2.43 | PSAP/OSBP/APOF/ |
| labyrinthine layer  blood vessel development | 3.97E-03 | 2.40 | HES1/JUNB/JUN/HEY2/ |
| negative regulation  of interleukin-6 production | 3.97E-03 | 2.40 | KLF2/TNFAIP3/ZC3H12A/ |
| telencephalon  development | 3.97E-03 | 2.40 | HES1/ |
| proteolysis involved  in cellular protein  catabolic process | 3.97E-03 | 2.40 | PSMB3/ |
| response to molecule  of bacterial origin | 4.34E-03 | 2.36 | CXCL2/IL8/TNFAIP3/ |
| oxygen transport | 4.34E-03 | 2.36 | HBA1/HBB/MYC/ |
| cotranslational protein  targeting to membrane | 4.34E-03 | 2.36 | SSR3/SRP14/ |
| glial cell migration | 4.34E-03 | 2.36 | TGFB2/CSPG4/ |
| positive regulation  of myoblast differentiation | 4.34E-03 | 2.36 | IGFBP3/HIF1AN/ |
| protein retention  in ER lumen | 4.34E-03 | 2.36 | OS9/ |
| hemostasis | 4.34E-03 | 2.36 | VWF/ |
| negative regulation  of catenin import into nucleus | 4.34E-03 | 2.36 | AXIN2/ |
| neutrophil activation | 4.34E-03 | 2.36 | IL8/ |
| positive regulation  of chemotaxis | 4.34E-03 | 2.36 | THBS1/ |
| regulation of reactive  oxygen species metabolic process | 4.34E-03 | 2.36 | IER3/ |
| antigen processing and  presentation of exogenous  peptide antigen via MHC  class I, TAP-dependent | 4.49E-03 | 2.35 | PSMB3/ |
| gluconeogenesis | 4.53E-03 | 2.34 | ATF3/ |
| epidermis development | 4.88E-03 | 2.31 | SPR/SPRR1B/ |
| positive regulation  of cell death | 4.91E-03 | 2.31 | HBA1/HBB/AXIN2/ |
| positive regulation  vascular endothelial  growth factor production | 4.91E-03 | 2.31 | IL1B/ |
| activation of MAPK  activity | 5.57E-03 | 2.25 | THBS1/C5AR1/IL1B/FPR1/CSPG4/ |
| cellular response  to interleukin-3 | 5.64E-03 | 2.25 | IL3RA/MT2A/IL3/ |
| negative regulation  of transcription by  transcription factor localization | 5.64E-03 | 2.25 | ID1/HEY2/ |
| ascending aorta  morphogenesis | 5.64E-03 | 2.25 | HES1/HEY2/ |
| negative regulation of  Wnt receptor signaling  pathway involved in  dorsal/ventral axis  specification | 5.64E-03 | 2.25 | AXIN2/SOST/ |
| positive regulation  of hepatocyte proliferation | 5.64E-03 | 2.25 | MED1/TNFAIP3/ |
| neutrophil apoptotic  process | 5.64E-03 | 2.25 | IL6/ |
| regulation of endothelial  cell proliferation | 5.64E-03 | 2.25 | ALDH1A2/ |
| serotonin secretion  by platelet | 5.64E-03 | 2.25 | P2RX1/ |
| endothelium development | 5.64E-03 | 2.25 | KDR/ |
| vascular smooth muscle  contraction | 5.64E-03 | 2.25 | ACTA2/ |
| sphingolipid catabolic  process | 5.64E-03 | 2.25 | GM2A/ |
| response to carbon monoxide | 5.64E-03 | 2.25 | EGR1/ |
| mitochondrial protein processing | 5.64E-03 | 2.25 | STOML2/ |
| tube morphogenesis | 5.64E-03 | 2.25 | PDPN/ |
| deltoid tuberosity development | 5.64E-03 | 2.25 | SCXA/ |
| regulation of growth rate | 5.64E-03 | 2.25 | PARP1/ |
| glutaminyl-tRNAGln  biosynthesis via  transamidation | 5.64E-03 | 2.25 | QRSL1/ |
| negative regulation  of calcium ion transmembrane  transporter activity | 5.64E-03 | 2.25 | SLN/ |
| positive regulation of  transcription from RNA  polymerase II promoter  involved in smooth muscle  cell differentiation | 5.64E-03 | 2.25 | NPNT/ |
| positive regulation of  cell division | 5.70E-03 | 2.24 | PPBP/IL1B/TGFB2/ |
| positive regulation of  cysteine-type endopeptidase  activity involved in  apoptotic process | 5.70E-03 | 2.24 | NLRP3/MYC/FOXQ1/ |
| metanephros development | 5.70E-03 | 2.24 | EYA1/CTSH/ |
| cell death | 5.73E-03 | 2.24 | SLC33A1/HEXA/BNIP3/VPS35/ADCK3/  FOSL2/FOS/TGFB2/OLR1/AXIN2/ |
| activation of cysteine-type  endopeptidase activity  involved in apoptotic process | 5.75E-03 | 2.24 | MUL1/NLRP3/P2RX1/ |
| toll-like receptor 4  signaling pathway | 5.98E-03 | 2.22 | FOS/JUN/ |
| negative regulation  of fibrinolysis | 6.06E-03 | 2.22 | THBS1/SERPINE1/THBD/ |
| positive regulation  of blood coagulation | 6.06E-03 | 2.22 | THBS1/SERPINE1/ |
| cellular response to  thyroid hormone stimulus | 6.06E-03 | 2.22 | MED1/CTSH/ |
| germ cell migration | 6.06E-03 | 2.22 | CXCL12/ |
| cellular response to  gamma radiation | 6.06E-03 | 2.22 | EGR1/ |
| biological_process | 6.25E-03 | 2.20 | C6/ZNF223/HNRNPUL2/ZNF75A/  HMGN3/PXMP2/WDR3/NOP9/C6orf106/TM4SF1/ZNF22/TIGD5/NR4A3/XG/SPANXC/MEGF9/KRTAP1-1/OR2L3/ |
| embryo development | 6.26E-03 | 2.20 | PDPN/RAI2/TGFB2/FOXQ1/THBD/ |
| phospholipid metabolic  process | 6.73E-03 | 2.17 | PIP4K2B/PI4K2A/LCLAT1/PIK3R3/  HADHA/ |
| response to DNA damage  stimulus | 6.73E-03 | 2.17 | NUAK1/CDKN1A/BTG2/MYC/PLK3/ |
|  |  |  |  |

**Supplemental Table9** Pathway analysis: categories of AF-related gene in female

| **Pathway Name** | **p-value** | **-lgP** | **Gene** | | | | | | | | | | | | | | | | | | | | | | | | | | | | | | |  | | | | | | | | | | |
| --- | --- | --- | --- | --- | --- | --- | --- | --- | --- | --- | --- | --- | --- | --- | --- | --- | --- | --- | --- | --- | --- | --- | --- | --- | --- | --- | --- | --- | --- | --- | --- | --- | --- | --- | --- | --- | --- | --- | --- | --- | --- | --- | --- | --- |
| Metabolic pathways | 1.68E-22 | 21.77 | ECHS1/EHHADH/ATP6V0E1/AK4/  SLC33A1/HEXA/PI4K2A/EXT2/  B3GALT2/ACLY/PGAM4/AHCYL1/  UGT2B4/ALDH1A2/SPR/SDHD/  LCLAT1/IMPAD1/ACO1/DLD/  ACAD8/COX7C/GALC/TRIT1/  ACOT2/ALG14/BCKDHB/HK2/  HIBADH/GANAB/HIBCH/CNDP2/  HADHA/P4HA1/GNS/ALAS2/  B3GNT5/MAT2A/ARG1/ST6GALNA/  C5DEGS2/ | | | | | | | | | | | | | | | | | | | | | | | | | | | | | | |  | | | | | | | | | | |
| Focal adhesion | 8.11E-17 | 16.09 | VWF/KDR/COL6A6/CRK/PIK3R3/  RELN/LAMB1/THBS1/JUN/SHC4/  VASP/ | | | | | | | | | | | | | | | | | | | | | | | | | | | | | | |  | | | | | | | | | | |
| PI3K-Akt signaling pathway | 2.76E-16 | 15.56 | VWF/KDR/COL6A6/TEK/CDK4/  PIK3R3/RELN/LAMB1/IL3RA/FGF18/  NR4A1/CDKN1A/THBS1/IL6/MYC/  CREB5/IL3/ | | | | | | | | | | | | | | | | | | | | | | | | | | | | | | |  | | | | | | | | | | |
| Malaria | 2.99E-16 | 15.52 | HBA2/HBA1/IL8/HBB/THBS1/IL6/  IL1B/TGFB2/ | | | | | | | | | | | | | | | | | | | | | | | | | | | | | | |  | | | | | | | | | | |
| Staphylococcus aureus infection | 1.04E-15 | 14.98 | C5AR1/FPR1 | | | | | | | | | | | | | | | | | | | | | | | | | | | | | | |  | | | | | | | | | | |
| Rheumatoid arthritis | 1.87E-15 | 14.73 | CXCL12/ATP6V0E1/TEK/IL8/FOS/IL6/  IL1B/JUN/TGFB2/ | | | | | | | | | | | | | | | | | | | | | | | | | | | | | | |  | | | | | | | | | | |
| Cytokine-cytokine receptor interaction | 2.17E-15 | 14.66 | CXCL12/KDR/CCL13/CCL18/IL3RA/CXCL2/  PF4/CXCR3/IL8/PPBP/IL6/IL1B/  TGFB2/IL3/ | | | | | | | | | | | | | | | | | | | | | | | | | | | | | | |  | | | | | | | | | | |
| Complement and coagulation cascades | 3.20E-14 | 13.50 | C6/VWF/PLAUR/C5AR1/SERPINE1/  THBD/ | | | | | | | | | | | | | | | | | | | | | | | | | | | | | | |  | | | | | | | | | | |
| NF-kappa B signaling pathway | 1.28E-13 | 12.89 | CXCL12/PARP1/CCL13/GADD45B/  CXCL2/IL8/TNFAIP3/IL1B/ | | | | | | | | | | | | | | | | | | | | | | | | | | | | | | |  | | | | | | | | | | |
| Phagosome | 1.18E-12 | 11.93 | ATP6V0E1/M6PR/STX7/THBS1/OLR1/ | | | | | | | | | | | | | | | | | | | | | | | | | | | | | | |  | | | | | | | | | | |
| HTLV-I infection | 1.82E-12 | 11.74 | NRP1/SLC25A6/CDK4/PIK3R3/ETS1/  ATF3/ZFP36/CDKN1A/EGR1/FOS/IL6/  JUN/MYC/TGFB2/ | | | | | | | | | | | | | | | | | | | | | | | | | | | | | | |  | | | | | | | | | | |
| Chemokine signaling pathway | 2.36E-11 | 10.63 | CXCL12/CCL13/CRK/PIK3R3/CCL18/  CXCL2/PF4/CXCR3/IL8/PPBP/SHC4/ | | | | | | | | | | | | | | | | | | | | | | | | | | | | | | |  | | | | | | | | | | |
| Hematopoietic cell lineage | 4.49E-11 | 10.35 | IL3RA/IL6/IL1B/IL3/ | | | | | | | | | | | | | | | | | | | | | | | | | | | | | | |  | | | | | | | | | | |
| Mineral absorption | 4.87E-11 | 10.31 | ATP1B4/SLC5A1/SLC31A1/MT2A/  MT1M/MT1X/MT1G/MT1F/MT1H/  MT1E/MT1B/ | | | | | | | | | | | | | | | | | | | | | | | | | | | | | | |  | | | | | | | | | | |
| ECM-receptor interaction | 3.18E-10 | 9.50 | VWF/COL6A6/RELN/LAMB1/CD47/  THBS1/ | | | | | | | | | | | | | | | | | | | | | | | | | | | | | | |  | | | | | | | | | | |
| Tuberculosis | 1.36E-09 | 8.87 | CALM3/CALM2/IL6/CLEC4E/IL1B/  TGFB2/CAMP/PLK3/ | | | | | | | | | | | | | | | | | | | | | | | | | | | | | | |  | | | | | | | | | | |
| Cell adhesion molecules (CAMs) | 1.95E-09 | 8.71 | JAM2/JAM3/CLDN1/ | | | | | | | | | | | | | | | | | | | | | | | | | | | | | | |  | | | | | | | | | | |
| Leishmaniasis | 2.51E-09 | 8.60 | FOS/IL1B/JUN/TGFB2/ | | | | | | | | | | | | | | | | | | | | | | | | | | | | | | |  | | | | | | | | | | |
| Influenza A | 3.89E-09 | 8.41 | SLC25A6/PIK3R3/IL8/SOCS3/IL6/IL1B/  JUN/NLRP3/IL3/ | | | | | | | | | | | | | | | | | | | | | | | | | | | | | | |  | | | | | | | | | | |
| Dilated cardiomyopathy | 5.40E-09 | 8.27 | MYL3/TGFB2/ | | | | | | | | | | | | | | | | | | | | | | | | | | | | | | |  | | | | | | | | | | |
| Intestinal immune network for IgA production | 7.35E-09 | 8.13 | CXCL12/IL6/ | | | | | | | | | | | | | | | | | | | | | | | | | | | | | | |  | | | | | | | | | | |
| Valine, leucine and isoleucine degradation | 1.27E-08 | 7.90 | ECHS1/EHHADH/DLD/ACAD8/  BCKDHB/HIBADH/HIBCH/HADHA/ | | | | | | | | | | | | | | | | | | | | | | | | | | | | | | |  | | | | | | | | | | |
| Lysosome | 1.52E-08 | 7.82 | NAGA/HEXA/M6PR/PSAP/GGA2/  AP3M1/GM2A/GALC/CTSH/GNS/CA1/ | | | | | | | | | | | | | | | | | | | | | | | | | | | | | | |  | | | | | | | | | | |
| Transcriptional misregulation in cancer | 2.13E-08 | 7.67 | IGFBP3/TAF15/EYA1/GOLPH3L/  CDKN1A/IL8/IL6/NR4A3/NFKBIZ/  MYC/IL3/ | | | | | | | | | | | | | | | | | | | | | | | | | | | | | | |  | | | | | | | | | | |
| Citrate cycle (TCA cycle) | 2.71E-08 | 7.57 | ACLY/SDHD/ACO1/DLD/ | | | | | | | | | | | | | | | | | | | | | | | | | | | | | | |  | | | | | | | | | | |
| Leukocyte transendothelial migration | 5.24E-08 | 7.28 | CXCL12/JAM2/JAM3/RAPGEF4/  PIK3R3/CLDN1/VASP/ | | | | | | | | | | | | | | | | | | | | | | | | | | | | | | |  | | | | | | | | | | |
| Toxoplasmosis | 8.91E-08 | 7.05 | PDK1/PIK3R3/LAMB1/TGFB2/ | | | | | | | | | | | | | | | | | | | | | | | | | | | | | | |  | | | | | | | | | | |
| Amoebiasis | 9.07E-08 | 7.04 | GNA11/PIK3R3/LAMB1/IL8/IL6/IL1B/  TGFB2/ARG1/ | | | | | | | | | | | | | | | | | | | | | | | | | | | | | | |  | | | | | | | | | | |
| Ribosome | 9.35E-08 | 7.03 | MRPS14/RPL8/MRPL33/RPS15/RPL11/  RPS16/RPL38/RPS29/ | | | | | | | | | | | | | | | | | | | | | | | | | | | | | | |  | | | | | | | | | | |
| Proteoglycans in cancer | 1.02E-07 | 6.99 | KDR/PDK1/PIK3R3/FGF18/CDKN1A/  THBS1/PLAUR/MYC/TGFB2/HBEGF/ | | | | | | | | | | | | | | | | | | | | | | | | | | | | | | |  | | | | | | | | | | |
| Hypertrophic cardiomyopathy (HCM) | 1.02E-07 | 6.99 | MYL3/IL6/TGFB2/ | | | | | | | | | | | | | | | | | | | | | | | | | | | | | | |  | | | | | | | | | | |
| African trypanosomiasis | 1.06E-07 | 6.98 | HBA2/HBA1/HBB/IL6/IL1B/ | | | | | | | | | | | | | | | | | | | | | | | | | | | | | | |  | | | | | | | | | | |
| Asthma | 1.06E-07 | 6.98 | IL3/ | | | | | | | | | | | | | | | | | | | | | | | | | | | | | | |  | | | | | | | | | | |
| Cardiac muscle contraction | 1.92E-07 | 6.72 | ATP1B4/MYL3/COX7C/ | | | | | | | | | | | | | | | | | | | | | | | | | | | | | | |  | | | | | | | | | | |
| Pathways in cancer | 3.09E-07 | 6.51 | MECOM/CDK4/CRK/PIK3R3/ETS1/  LAMB1/FGF18/CDKN1A/IL8/FOS/IL6/  JUN/MYC/TGFB2/AXIN2/ | | | | | | | | | | | | | | | | | | | | | | | | | | | | | | |  | | | | | | | | | | |
| HIF-1 signaling pathway | 3.58E-07 | 6.45 | PDK1/TEK/PIK3R3/HK2/CDKN1A/IL6/SERPINE1/ | | | | | | | | | | | | | | | | | | | | | | | | | | | | | | |  | | | | | | | | | | |
| Hepatitis B | 3.67E-07 | 6.44 | CDK4/PIK3R3/CDKN1A/IL8/FOS/IL6/  JUN/MYC/TGFB2/CREB5/ | | | | | | | | | | | | | | | | | | | | | | | | | | | | | | |  | | | | | | | | | | |
| Systemic lupus erythematosus | 6.02E-07 | 6.22 | C6/HIST1H4H/ | | | | | | | | | | | | | | | | | | | | | | | | | | | | | | |  | | | | | | | | | | |
| Renin-angiotensin system | 8.03E-07 | 6.10 | CMA1/CP/ | | | | | | | | | | | | | | | | | | | | | | | | | | | | | | |  | | | | | | | | | | |
| Herpes simplex infection | 9.19E-07 | 6.04 | PER3/PER1/SOCS3/FOS/IL6/IL1B/JUN/ | | | | | | | | | | | | | | | | | | | | | | | | | | | | | | |  | | | | | | | | | | |
| MAPK signaling pathway | 1.37E-06 | 5.86 | MECOM/CRK/FGF18/NR4A1/  GADD45B/JUND/FOS/IL1B/DUSP1/  JUN/MYC/TGFB2/ | | | | | | | | | | | | | | | | | | | | | | | | | | | | | | |  | | | | | | | | | | |
| Legionellosis | 1.67E-06 | 5.78 | BNIP3/EEF1G/CXCL2/IL8/IL6/IL1B/ | | | | | | | | | | | | | | | | | | | | | | | | | | | | | | |  | | | | | | | | | | |
| PPAR signaling pathway | 3.55E-06 | 5.45 | EHHADH/CP/OLR1/ | | | | | | | | | | | | | | | | | | | | | | | | | | | | | | |  | | | | | | | | | | |
| Protein digestion and absorption | 6.41E-06 | 5.19 | ATP1B4/COL6A6/CP/ELN/CTRB1/ | | | | | | | | | | | | | | | | | | | | | | | | | | | | | | |  | | | | | | | | | | |
| Pertussis | 6.47E-06 | 5.19 | CALM3/CALM2/IL8/FOS/IL6/IL1B/  JUN/NLRP3/ | | | | | | | | | | | | | | | | | | | | | | | | | | | | | | |  | | | | | | | | | | |
| Salivary secretion | 8.28E-06 | 5.08 | ATP1B4/CALM3/CALM2/CAMP/CST5/ | | | | | | | | | | | | | | | | | | | | | | | | | | | | | | |  | | | | | | | | | | |
| Huntington's disease | 9.48E-06 | 5.02 | SLC25A6/SDHD/COX7C/CP/CREB5/ | | | | | | | | | | | | | | | | | | | | | | | | | | | | | | |  | | | | | | | | | | |
| Osteoclast differentiation | 9.66E-06 | 5.02 | FHL2/PIK3R3/JUNB/SOCS3/JUND/  FOSL2/FOSB/FOS/IL1B/JUN/TGFB2/ | | | | | | | | | | | | | | | | | | | | | | | | | | | | | | |  | | | | | | | | | | |
| Glycolysis / Gluconeogenesis | 1.10E-05 | 4.96 | PGAM4/DLD/HK2/GALM/ | | | | | | | | | | | | | | | | | | | | | | | | | | | | | | |  | | | | | | | | | | |
| Alzheimer's disease | 1.27E-05 | 4.90 | CALM3/SDHD/COX7C/CALM2/IL1B/ | | | | | | | | | | | | | | | | | | | | | | | | | | | | | | |  | | | | | | | | | | |
| p53 signaling pathway | 1.48E-05 | 4.83 | IGFBP3/CDK4/GADD45B/CDKN1A/  THBS1/SERPINE1/SFN/ | | | | | | | | | | | | | | | | | | | | | | | | | | | | | | |  | | | | | | | | | | |
| Graft-versus-host disease | 1.67E-05 | 4.78 | IL6/IL1B/ | | | | | | | | | | | | | | | | | | | | | | | | | | | | | | |  | | | | | | | | | | |
| NOD-like receptor signaling pathway | 1.82E-05 | 4.74 | CXCL2/IL8/IL6/TNFAIP3/IL1B/NLRP3/ | | | | | | | | | | | | | | | | | | | | | | | | | | | | | | |  | | | | | | | | | | |
| Estrogen signaling pathway | 2.69E-05 | 4.57 | CALM3/CALM2/PIK3R3/FOS/JUN/  SHC4/CREB5/HBEGF/ | | | | | | | | | | | | | | | | | | | | | | | | | | | | | | |  | | | | | | | | | | |
| Small cell lung cancer | 2.77E-05 | 4.56 | MALL/CDK4/PIK3R3/LAMB1/MYC/ | | | | | | | | | | | | | | | | | | | | | | | | | | | | | | |  | | | | | | | | | | |
| Regulation of actin cytoskeleton | 2.82E-05 | 4.55 | PIP4K2B/CRK/PIK3R3/FGF18/ | | | | | | | | | | | | | | | | | | | | | | | | | | | | | | |  | | | | | | | | | | |
| Chronic myeloid leukemia | 2.99E-05 | 4.52 | MECOM/CDK4/CRK/PIK3R3/CDKN1A/  MYC/TGFB2/SHC4/ | | | | | | | | | | | | | | | | | | | | | | | | | | | | | | |  | | | | | | | | | | |
| Oxidative phosphorylation | 3.46E-05 | 4.46 | ATP6V0E1/SDHD/COX7C/ | | | | | | | | | | | | | | | | | | | | | | | | | | | | | | |  | | | | | | | | | | |
| Bladder cancer | 3.57E-05 | 4.45 | CDK4/CDKN1A/IL8/THBS1/MYC/ | | | | | | | | | | | | | | | | | | | | | | | | | | | | | | |  | | | | | | | | | | |
| Measles | 3.79E-05 | 4.42 | CDK4/PIK3R3/IL6/TNFAIP3/IL1B/ | | | | | | | | | | | | | | | | | | | | | | | | | | | | | | |  | | | | | | | | | | |
| Vascular smooth muscle contraction | 4.46E-05 | 4.35 | CALM3/PRKCH/GNA11/CALM2/ACTA2/PPP1R14A/KCNMB1/ | | | | | | | | | | | | | | | | | | | | | | | | | | | | | | |  | | | | | | | | | | |
| Epstein-Barr virus infection | 4.49E-05 | 4.35 | PIK3R3/CDKN1A/TNFAIP3/JUN/MYC/ | | | | | | | | | | | | | | | | | | | | | | | | | | | | | | |  | | | | | | | | | | |
| Chagas disease (American trypanosomiasis) | 4.59E-05 | 4.34 | GNA11PIK3R3/IL8/FOS/IL6/IL1B/  JUN/SERPINE1/TGFB2/ | | | | | | | | | | | | | | | | | | | | | | | | | | | | | | |  | | | | | | | | | | |
| Tryptophan metabolism | 5.31E-05 | 4.27 | ECHS1/EHHADH/CYP1B1/HADHA/ | | | | | | | | | | | | | | | | | | | | | | | | | | | | | | |  | | | | | | | | | | |
| Hippo signaling pathway | 6.06E-05 | 4.22 | ID1/IPP/LIMD1/AREG/MYC/  SERPINE1/TGFB2/AXIN2/ | | | | | | | | | | | | | | | | | | | | | | | | | | | | | | |  | | | | | | | | | | |
| Toll-like receptor signaling pathway | 6.21E-05 | 4.21 | PIK3R3/IL8/FOS/IL6/IL1B/JUN/ | | | | | | | | | | | | | | | | | | | | | | | | | | | | | | |  | | | | | | | | | | |
| TGF-beta signaling pathway | 8.12E-05 | 4.09 | ID1/THBS1/MYC/TGFB2/ | | | | | | | | | | | | | | | | | | | | | | | | | | | | | | |  | | | | | | | | | | |
| Epithelial cell signaling in Helicobacter pylori infection | 9.00E-05 | 4.05 | ATP6V0E1/JAM2/JAM3/IL8/JUN/  HBEGF/ | | | | | | | | | | | | | | | | | | | | | | | | | | | | | | |  | | | | | | | | | | |
| Propanoate metabolism | 9.24E-05 | 4.03 | ECHS1/EHHADH/HIBCH/HADHA/ | | | | | | | | | | | | | | | | | | | | | | | | | | | | | | |  | | | | | | | | | | |
| Biosynthesis of amino acids | 1.16E-04 | 3.93 | PGAM4/ACO1/ARG1/ | | | | | | | | | | | | | | | | | | | | | | | | | | | | | | |  | | | | | | | | | | |
| Parkinson's disease | 1.19E-04 | 3.92 | SLC25A6/SDHD/COX7C/ | | | | | | | | | | | | | | | | | | | | | | | | | | | | | | |  | | | | | | | | | | |
| Melanoma | 1.31E-04 | 3.88 | CDK4/PIK3R3/FGF18/CDKN1A/ | | | | | | | | | | | | | | | | | | | | | | | | | | | | | | |  | | | | | | | | | | |
| Type I diabetes mellitus | 1.79E-04 | 3.75 | IL1B/ | | | | | | | | | | | | | | | | | | | | | | | | | | | | | | |  | | | | | | | | | | |
| Insulin signaling pathway | 2.45E-04 | 3.61 | CALM3/CRK/CALM2/PIK3R3/HK2/  SOCS3/SHC4 | | | | | | | | | | | | | | | | | | | | | | | | | | | | | | |  | | | | | | | | | | |
| Cell cycle | 2.62E-04 | 3.58 | CDK4/GADD45B/CDKN1A/CCNH/  MYC/TGFB2/SFN/ | | | | | | | | | | | | | | | | | | | | | | | | | | | | | | |  | | | | | | | | | | |
| Glioma | 3.46E-04 | 3.46 | CALM3/CDK4/CALM2/PIK3R3/  CDKN1A/SHC4/ | | | | | | | | | | | | | | | | | | | | | | | | | | | | | | |  | | | | | | | | | | |
| Calcium signaling pathway | 4.04E-04 | 3.39 | SLC25A6/CALM3/GNA11/CALM2/  P2RX1/ | | | | | | | | | | | | | | | | | | | | | | | | | | | | | | |  | | | | | | | | | | |
| beta-Alanine metabolism | 4.52E-04 | 3.34 | ECHS1/EHHADH/HIBCH/CNDP2/  HADHA/ | | | | | | | | | | | | | | | | | | | | | | | | | | | | | | |  | | | | | | | | | | |
| Endocytosis | 4.61E-04 | 3.34 | KDR/FOLR2/WWP1/EHD4/TGFB2/  ASAP2/ | | | | | | | | | | | | | | | | | | | | | | | | | | | | | | |  | | | | | | | | | | |
| Tight junction | 5.69E-04 | 3.24 | JAM2/PRKCH/JAM3/CDK4/JUN/  CLDN1/ | | | | | | | | | | | | | | | | | | | | | | | | | | | | | | |  | | | | | | | | | | |
| Bile secretion | 7.59E-04 | 3.12 | ATP1B4/ABCG2/UGT2B4/SLC4A4/  SLC5A1/ | | | | | | | | | | | | | | | | | | | | | | | | | | | | | | |  | | | | | | | | | | |
| ErbB signaling pathway | 8.00E-04 | 3.10 | CRK/PIK3R3/CDKN1A/AREG/JUN/  MYC/SHC4/HBEGF/ | | | | | | | | | | | | | | | | | | | | | | | | | | | | | | |  | | | | | | | | | | |
| Salmonella infection | 8.00E-04 | 3.10 | CXCL2/IL8/FOS/IL6/IL1B/JUN/ | | | | | | | | | | | | | | | | | | | | | | | | | | | | | | |  | | | | | | | | | | |
| Apoptosis | 8.00E-04 | 3.10 | PIK3R3/IL3RA/IL1B/IL3/ | | | | | | | | | | | | | | | | | | | | | | | | | | | | | | |  | | | | | | | | | | |
| Carbohydrate digestion and absorption | 8.78E-04 | 3.06 | ATP1B4/SLC5A1/PIK3R3/HK2/ | | | | | | | | | | | | | | | | | | | | | | | | | | | | | | |  | | | | | | | | | | |
| Endocrine and other factor-regulated calcium reabsorption | 1.49E-03 | 2.83 | ATP1B4/ | | | | | | | | | | | | | | | | | | | | | | | | | | | | | | |  | | | | | | | | | | |
| Protein processing in endoplasmic reticulum | 1.50E-03 | 2.82 | SSR3/TXNDC5/PREB/RNF185/SEL1L/  OS9/GANAB/ | | | | | | | | | | | | | | | | | | | | | | | | | | | | | | |  | | | | | | | | | | |
| Prion diseases | 1.54E-03 | 2.81 | C6/EGR1/IL6/IL1B | | | | | | | | | | | | | | | | | | | | | | | | | | | | | | |  | | | | | | | | | | |
| Glycine, serine and threonine metabolism | 1.79E-03 | 2.75 | PGAM4/DLD/ALAS2/ | | | | | | | | | | | | | | | | | | | | | | | | | | | | | | |  | | | | | | | | | | |
| Peroxisome | 1.82E-03 | 2.74 | EHHADH/PXMP2/ | | | | | | | | | | | | | | | | | | | | | | | | | | | | | | |  | | | | | | | | | | |
| Hepatitis C | 1.84E-03 | 2.73 | PDK1/PIK3R3/CDKN1A/IL8/SOCS3/  CLDN1/ | | | | | | | | | | | | | | | | | | | | | | | | | | | | | | |  | | | | | | | | | | |
| Renal cell carcinoma | 1.95E-03 | 2.71 | CRK/PIK3R3/ETS1/JUN/TGFB2/ | | | | | | | | | | | | | | | | | | | | | | | | | | | | | | |  | | | | | | | | | | |
| Neurotrophin signaling pathway | 2.61E-03 | 2.58 | CALM3/PDK1/CRK/CALM2/PIK3R3/  JUN/SHC4/ | | | | | | | | | | | | | | | | | | | | | | | | | | | | | | |  | | | | | | | | | | |
| Insulin secretion | 3.03E-03 | 2.52 | ATP1B4/GNA11/RAPGEF4/CREB5/  KCNMB1/ | | | | | | | | | | | | | | | | | | | | | | | | | | | | | | |  | | | | | | | | | | |
| Pyruvate metabolism | 3.12E-03 | 2.51 | DLD/ | | | | | | | | | | | | | | | | | | | | | | | | | | | | | | |  | | | | | | | | | | |
| 2-Oxocarboxylic acid metabolism | 3.17E-03 | 2.50 | ACO1/ | | | | | | | | | | | | | | | | | | | | | | | | | | | | | | |  | | | | | | | | | | |
| Butanoate metabolism | 3.63E-03 | 2.44 | ECHS1/EHHADH/HADHA/ | | | | | | | | | | | | | | | | | | | | | | | | | | | | | | |  | | | | | | | | | | |
| Arginine and proline metabolism | 3.71E-03 | 2.43 | CNDP2/P4HA1/ARG1/ | | | | | | | | | | | | | | | | | | | | | | | | | | | | | | |  | | | | | | | | | | |
| Viral carcinogenesis | 4.29E-03 | 2.37 | HIST1H4H/CDK4/PIK3R3/CDKN1A/  JUN/CREB5/ | | | | | | | | | | | | | | | | | | | | | | | | | | | | | | |  | | | | | | | | | | |
| Fatty acid degradation | 4.53E-03 | 2.34 | ECHS1/EHHADH/HADHA/ | | | | | | | | | | | | | | | | | | | | | | | | | | | | | | |  | | | | | | | | | | |
| B cell receptor signaling pathway | 4.88E-03 | 2.31 | PIK3R3/FOS/JUN/ | | | | | | | | | | | | | | | | | | | | | | | | | | | | | | |  | | | | | | | | | | |
| Fc gamma R-mediated phagocytosis | 5.19E-03 | 2.29 | CRK/PIK3R3/ASAP2/VASP/ | | | | | | | | | | | | | | | | | | | | | | | | | | | | | | |  | | | | | | | | | | |
| Pancreatic secretion | 5.98E-03 | 2.22 | ATP1B4/SLC4A4/CP/CTRB1/ | | | | | | | | | | | | | | | | | | | | | | | | | | | | | | |  | | | | | | | | | | |
| Colorectal cancer | 6.03E-03 | 2.22 | PIK3R3/FOS/JUN/MYC/TGFB2/AXIN2/ | | | | | | | | | | | | | | | | | | | | | | | | | | | | | | |  | | | | | | | | | | |
| Biosynthesis of unsaturated fatty acids | 7.22E-03 | 2.14 | ACOT2/HADHA/ | | | | | | | | | | | | | | | | | | | | | | | | | | | | | | |  | | | | | | | | | | |
| Jak-STAT signaling pathway | 7.85E-03 | 2.10 | PIK3R3/IL3RA/SOCS3/IL6/MYC/IL3/ | | | | | | | | | | | | | | | | | | | | | | | | | | | | | | |  | | | | | | | | | | |
| Cytosolic DNA-sensing pathway | 8.59E-03 | 2.07 | IL6/IL1B/IL3/ | | | | | | | | | | | | | | | | | | | | | | | | | | | | | | |  | | | | | | | | | | |
| Fructose and mannose metabolism | 9.64E-03 | 2.02 | HK2/ | | | | | | | | | | | | | | | | | | | | | | | | | | | | | | |  | | | | | | | | | | |
| Fatty acid elongation | 1.02E-02 | 1.99 | ECHS1/ACOT2/HADHA/ | | | | | | | | | | | | | | | | | | | | | | | | | | | | | | |  | | | | | | | | | | |
| Wnt signaling pathway | 1.02E-02 | 1.99 | JUN/MYC/AXIN2/SOST/ | | | | | | | | | | | | | | | | | | | | | | | | | | | | | | |  | | | | | | | | | | |
| Amphetamine addiction | 1.19E-02 | 1.92 | CALM3/CALM2/FOSB/FOS/JUN/  CREB5/ | | | | | | | | | | | | | | | | | | | | | | | | | | | | | | |  | | | | | | | | | | |
| Gap junction | 1.29E-02 | 1.89 | GNA11/JUN/GJD2/ | | | | | | | | | | | | | | | | | | | | | | | | | | | | | | |  | | | | | | | | | | |
| T cell receptor signaling pathway | 1.30E-02 | 1.89 | PDK1/CDK4/PIK3R3/FOS/JUN/ | | | | | | | | | | | | | | | | | | | | | | | | | | | | | | |  | | | | | | | | | | |
| Aldosterone-regulated sodium reabsorption | 1.36E-02 | 1.86 | ATP1B4/PIK3R3/SFN/ | | | | | | | | | | | | | | | | | | | | | | | | | | | | | | |  | | | | | | | | | | |
| Glycerolipid metabolism | 1.39E-02 | 1.86 | LCLAT1/ | | | | | | | | | | | | | | | | | | | | | | | | | | | | | | |  | | | | | | | | | | |
| GnRH signaling pathway | 1.57E-02 | 1.81 | CALM3/GNA11/JUN/HBEGF/ | | | | | | | | | | | | | | | | | | | | | | | | | | | | | | |  | | | | | | | | | | |
| Glycosphingolipid biosynthesis - globo series | 1.68E-02 | 1.78 | NAGA/HEXA/CALM2/ | | | | | | | | | | | | | | | | | | | | | | | | | | | | | | |  | | | | | | | | | | |
| Glycosphingolipid biosynthesis - ganglio series | 2.05E-02 | 1.69 | SLC33A1/HEXA/ST6GALNAC5/ | | | | | | | | | | | | | | | | | | | | | | | | | | | | | | |  | | | | | | | | | | |
| Circadian entrainment | 2.13E-02 | 1.67 | CALM3/PER3/CALM2/PER1/FOS/ | | | | | | | | | | | | | | | | | | | | | | | | | | | | | | |  | | | | | | | | | | |
| Galactose metabolism | 2.65E-02 | 1.58 | HK2/ | | | | | | | | | | | | | | | | | | | | | | | | | | | | | | |  | | | | | | | | | | |
| Aminoacyl-tRNA biosynthesis | 2.66E-02 | 1.58 | KARS/AARS/HARS2/ | | | | | | | | | | | | | | | | | | | | | | | | | | | | | | |  | | | | | | | | | | |
| Alcoholism | 2.83E-02 | 1.55 | HIST1H4H/CALM3/CALM2/FOSB/  SHC4/CREB5/ | | | | | | | | | | | | | | | | | | | | | | | | | | | | | | |  | | | | | | | | | | |
| Circadian rhythm | 2.97E-02 | 1.53 | PER3/PER1/BHLHE40/ | | | | | | | | | | | | | | | | | | | | | | | | | | | | | | |  | | | | | | | | | | |
| Ovarian steroidogenesis | 4.11E-02 | 1.39 | CYP1B1/ACOT2/ | | | | | | | | | | | | | | | | | | | | | | | | | | | | | | |  | | | | | | | | | | |
| Prostate cancer | 4.14E-02 | 1.38 | PIK3R3/CDKN1A/CREB5/ | | | | | | | | | | | | | | | | | | | | | | | | | | | | | | |  | | | | | | | | | | |
| Neuroactive ligand-receptor interaction | 4.16E-02 | 1.38 | APLNR/GRIK2/C5AR1/FPR1/P2RX1/  GALR3/ | | | | | | | | | | | | | | | | | | | | | | | | | | | | | | |  | | | | | | | | | | |
| Fc epsilon RI signaling pathway | 4.28E-02 | 1.37 | PDK1/PIK3R3/IL3/ | | | | | | | | | | | | | | | | | | | | | | | | | | | | | | |  | | | | | | | | | | |
| Endometrial cancer | 4.44E-02 | 1.35 | PIK3R3/MYC/AXIN2/ | | | | | | | | | | | | | | | | | | | | | | | | | | | | | | |  | | | | | | | | | | |
| Cholinergic synapse | 4.92E-02 | 1.31 | GNA11/PIK3R3/FOS/CREB5/ | | | | | | | | | | | | | | | | | | | | | | | | | | | | | | |  | | | | | | | | | | |
|  |  |  |  |  |  |  |  |  |  |  |  |  |  |  |  |  |  |  |  |  |  |  |  |  |  |  |  |  |  |  |  |  |  | |  |  |  |  |  |  |  |  |  |  |

**Supplemental Table10** GO analysis: categories of AF-related gene in male

| **GO Name** | **p-value** | **-lgP** | **Gene** |  | | | | | | | | | | | | | | | | | | | | | | | | | | | | | | | | | | | | | | | | | | | |
| --- | --- | --- | --- | --- | --- | --- | --- | --- | --- | --- | --- | --- | --- | --- | --- | --- | --- | --- | --- | --- | --- | --- | --- | --- | --- | --- | --- | --- | --- | --- | --- | --- | --- | --- | --- | --- | --- | --- | --- | --- | --- | --- | --- | --- | --- | --- | --- |
| small molecule  metabolic process | 1.52E-74 | 73.82 | C6/FMO2/GPD1L/ACAT1/SMC2/  PGK1/UQCRB/ISCU/HIBADH/  NDUFB6/COX7C/NDUFA1/  PRKAR1A/NDUFA6/ASAH1/NDUFA8/  COX5B/HK2/PTGR1/UQCR10/GLYAT/  PLA2G16/COX7B/IDH3B/RAPGEF4/  COQ7/PSME2/ATP5O/ATP5G1/  PIK3R3/TXNRD1/PSMC5/UROD/C3/  HSPG2/CDA/GGT5/CPT1A/NAGLU/  HS3ST3A1/CSPG4/LGMN/SCAP/  AZIN1/CREBBP/ |  | | | | | | | | | | | | | | | | | | | | | | | | | | | | | | | | | | | | | | | | | | | |
| respiratory electron  transport chain | 8.91E-46 | 45.05 | UQCRB/NDUFB6/COX7C/NDUFA1/  NDUFA6/NDUFA8/COX5B/UQCR10/  COX7B/ATP5O/ATP5G1/ |  | | | | | | | | | | | | | | | | | | | | | | | | | | | | | | | | | | | | | | | | | | | |
| cell adhesion | 3.51E-30 | 29.45 | F5/POSTN/C3/SPON1/WISP1/NEO1/  LPP/CD97/SIRPA/TGFBI/PXN/  CERCAM/ITGB3/EPHA2/ |  | | | | | | | | | | | | | | | | | | | | | | | | | | | | | | | | | | | | | | | | | | | |
| extracellular  matrix organization | 8.65E-30 | 29.06 | COL1A1/COL3A1/POSTN/COL1A2/  FBN1/ELN/NPNT/HSPG2/FBLN5/  ADAMTS2/OLFML2B/FBLN1/  TGFB2/LTBP3/TGFBI/ITGB3/ |  | | | | | | | | | | | | | | | | | | | | | | | | | | | | | | | | | | | | | | | | | | | |
| signal transduction | 1.71E-21 | 20.77 | PDE7A/PRKAR1A/PRKAG1/PRKG1/  BST2/TXNRD1/C3/IGFBP5/GRN/  HBEGF/WISP1/NR2F2/ECM1/PXN/  PLAU/CANT1/CREBBP/ |  | | | | | | | | | | | | | | | | | | | | | | | | | | | | | | | | | | | | | | | | | | | |
| positive regulation  of transcription from RNA  polymerase II promoter | 3.55E-18 | 17.45 | C6/HSF2/HMGN3/KDM3A/HAX1/  TAF7/C3/HEY2/INHBA/FSTL3/FZD8/ELF4/PFN1/GLI3/FOXO6/NFIC/ATF4/EN1/TEAD3/BCL9L/ZMIZ1/NFIX/SMAD3/GLI2/MAF/CREBBP/ |  | | | | | | | | | | | | | | | | | | | | | | | | | | | | | | | | | | | | | | | | | | | |
| negative regulation  of cell proliferation | 1.25E-17 | 16.90 | IFIT3/RGCC/INHBA/SLIT3/TGFB2/  GDF11/RBM38/GLI3/FOXO6/ |  | | | | | | | | | | | | | | | | | | | | | | | | | | | | | | | | | | | | | | | | | | | |
| inflammatory response | 1.98E-17 | 16.70 | C3/TRIL/ECM1/GGT5/CD97/CCR7/ |  | | | | | | | | | | | | | | | | | | | | | | | | | | | | | | | | | | | | | | | | | | | |
| gene expression | 3.92E-17 | 16.41 | F5/C6/SMC2/NR1D2/TTF1/KARS/  HNRNPH2/RNMT/RRN3/PSME2/  HNRNPA2B1/SPCS2/PSMC5/C3/  PTBP1/TEAD3/SMAD3/CREBBP/ |  | | | | | | | | | | | | | | | | | | | | | | | | | | | | | | | | | | | | | | | | | | | |
| mitochondrial electron  transport, NADH to ubiquinone | 2.23E-16 | 15.65 | NDUFB6/NDUFA1/NDUFA6/NDUFA8/ |  | | | | | | | | | | | | | | | | | | | | | | | | | | | | | | | | | | | | | | | | | | | |
| blood coagulation | 7.80E-16 | 15.11 | F5/PRKAR1A/EGF/RAPGEF4/PRKG1/  COL1A1/COL1A2/FLNA/FBLN5/CLU/TGFB2/SERPING1/C1QB/PFN1/  PROCR/SIRPA/ACTB/PLAU/ITGB3/ |  | | | | | | | | | | | | | | | | | | | | | | | | | | | | | | | | | | | | | | | | | | | |
| apoptotic process | 3.93E-15 | 14.41 | F5/C6/TNFRSF19/SMC2/GHITM/  PSME2/PSMC5/IER3/C1QB/PHLDA1/KRT18/TRAF7/GLI2/DAP/ |  | | | | | | | | | | | | | | | | | | | | | | | | | | | | | | | | | | | | | | | | | | | |
| mitochondrial ATP synthesis  coupled proton transport | 5.11E-15 | 14.29 | STOML2/ATP5O/ATP5G1/ |  | | | | | | | | | | | | | | | | | | | | | | | | | | | | | | | | | | | | | | | | | | | |
| tricarboxylic acid cycle | 7.51E-15 | 14.12 | IDH3B/ |  | | | | | | | | | | | | | | | | | | | | | | | | | | | | | | | | | | | | | | | | | | | |
| collagen fibril organization | 8.70E-15 | 14.06 | COL1A1/COL3A1/COL1A2/  ADAMTS2/TGFB2/ |  | | | | | | | | | | | | | | | | | | | | | | | | | | | | | | | | | | | | | | | | | | | |
| skeletal system development | 1.97E-14 | 13.71 | COL1A1/COL3A1/POSTN/COL1A2/  FBN1/C3/GDF11/EN1/EPHA2/CLEC3B/GLI2/ |  | | | | | | | | | | | | | | | | | | | | | | | | | | | | | | | | | | | | | | | | | | | |
| muscle contraction | 2.66E-14 | 13.58 | HSBP1/ACTA1/ACTG2/TPM2/PXN/ |  | | | | | | | | | | | | | | | | | | | | | | | | | | | | | | | | | | | | | | | | | | | |
| immune response | 3.03E-14 | 13.52 | IFI44L/HLA-DMA/PRG4/C3/SBSPON/  SUSD2/C1QB/C1QC/CD276/CD97/PROCR/PTGER4/SMAD3/IFITM3/CCR7/ |  | | | | | | | | | | | | | | | | | | | | | | | | | | | | | | | | | | | | | | | | | | | |
| platelet degranulation | 3.87E-14 | 13.41 | F5/EGF/FLNA/CLU/TGFB2/  SERPING1/PFN1/ITGB3/ |  | | | | | | | | | | | | | | | | | | | | | | | | | | | | | | | | | | | | | | | | | | | |
| negative regulation of  transcription from  RNA polymerase II promoter | 5.66E-14 | 13.25 | F5/ZNF189/TXNIP/HSBP1/ZNF675/  TAF7/HEY2/C1QB/NR2F2/FZD8/GLI3/NFIC/NFIX/SMAD3/GLI2/MAF/  CREBBP/ |  | | | | | | | | | | | | | | | | | | | | | | | | | | | | | | | | | | | | | | | | | | | |
| proteolysis | 9.30E-14 | 13.03 | SMC2/SPCS2/ADAMTS2/SERPING1/  PAPPA/XPNPEP2/LGMN/PLAU/  PRSS23/PRSS45/ |  | | | | | | | | | | | | | | | | | | | | | | | | | | | | | | | | | | | | | | | | | | | |
| platelet activation | 6.40E-13 | 12.19 | F5/EGF/RAPGEF4/COL1A1/COL3A1/  COL1A2/FLNA/CLU/TGFB2/  SERPING1/PFN1/ |  | | | | | | | | | | | | | | | | | | | | | | | | | | | | | | | | | | | | | | | | | | | |
| innate immune response | 8.49E-13 | 12.07 | C6/PRKAR1A/TXNIP/RSAD2/IFIT5/  BST2/C3/ACTG1/CLU/HBEGF/  SERPING1/C1QB/TRIL/C1QC/LGMN/ACTB/ITGB3/FGF23/CREBBP/ |  | | | | | | | | | | | | | | | | | | | | | | | | | | | | | | | | | | | | | | | | | | | |
| cellular lipid metabolic process | 1.45E-12 | 11.84 | GPD1L/ACAT1/TXNRD1/CPT1A/  CREBBP/ |  | | | | | | | | | | | | | | | | | | | | | | | | | | | | | | | | | | | | | | | | | | | |
| mitotic cell cycle | 1.66E-12 | 11.78 | C6/NEDD1/SMC2/SKA2/LIN9/PSME2/  PSMC5/ARPP19/C3/ |  | | | | | | | | | | | | | | | | | | | | | | | | | | | | | | | | | | | | | | | | | | | |
| angiogenesis | 2.03E-12 | 11.69 | EGF/HSPG2/TGFB2/ECM1/MEOX2/  TGFBI/CSPG4/EPHA2/ |  | | | | | | | | | | | | | | | | | | | | | | | | | | | | | | | | | | | | | | | | | | | |
| ATP catabolic process | 2.31E-12 | 11.64 | C6/SMC2/ATP5O/ |  | | | | | | | | | | | | | | | | | | | | | | | | | | | | | | | | | | | | | | | | | | | |
| heart development | 3.45E-12 | 11.46 | COX17/COL3A1/FBN1/TGFB2/  ADAP2/GLI3/GLI2/DAP/ |  | | | | | | | | | | | | | | | | | | | | | | | | | | | | | | | | | | | | | | | | | | | |
| lipid metabolic process | 4.50E-12 | 11.35 | PLCL2/ASAH1/HSPG2CLU/NR2F2/  FADS3/LRP10/ |  | | | | | | | | | | | | | | | | | | | | | | | | | | | | | | | | | | | | | | | | | | | |
| response to hypoxia | 5.09E-12 | 11.29 | TGFB2/SOD3/PLAU/SMAD3/SCAP/  CREBBP/ |  | | | | | | | | | | | | | | | | | | | | | | | | | | | | | | | | | | | | | | | | | | | |
| transforming growth factor  beta receptor signaling pathway | 1.20E-11 | 10.92 | COL3A1/COL1A2/TGFB2/LTBP3/  SMAD3/ |  | | | | | | | | | | | | | | | | | | | | | | | | | | | | | | | | | | | | | | | | | | | |
| glucose metabolic process | 1.90E-11 | 10.72 | PGK1/IGFBP5/CPT1A/ |  | | | | | | | | | | | | | | | | | | | | | | | | | | | | | | | | | | | | | | | | | | | |
| cellular nitrogen compound  metabolic process | 2.25E-11 | 10.65 | C6/ACAT1/SMC2/HIBADH/PSME2/  PSMC5/AZIN1/ |  | | | | | | | | | | | | | | | | | | | | | | | | | | | | | | | | | | | | | | | | | | | |
| chemotaxis | 2.83E-11 | 10.55 | PLP2/PLAU/CCR7/ |  | | | | | | | | | | | | | | | | | | | | | | | | | | | | | | | | | | | | | | | | | | | |
| regulation of cellular  amino acid metabolic process | 2.94E-11 | 10.53 | C6/SMC2/PSME2/PSMC5/AZIN1/ |  | | | | | | | | | | | | | | | | | | | | | | | | | | | | | | | | | | | | | | | | | | | |
| axon guidance | 3.48E-11 | 10.46 | PRNP/COL3A1/ACTG1/SLIT3/TGFB2/  NEO1/ACTB/GLI3/ITGB3/PLXNA1/  GLI2/ |  | | | | | | | | | | | | | | | | | | | | | | | | | | | | | | | | | | | | | | | | | | | |
| carbohydrate metabolic process | 4.02E-11 | 10.40 | GPD1L/PGK1/HK2/HSPG2/NAGLU/  HS3ST3A1/CSPG4/ |  | | | | | | | | | | | | | | | | | | | | | | | | | | | | | | | | | | | | | | | | | | | |
| positive regulation of  transcription, DNA-dependent | 4.05E-11 | 10.39 | C6/EGF/RRN3/KDM3A/TRIP4/  PSMC5/COL1A1/INHBA/NR2F2/  SOST/ELF4/GLI3/TP53INP2/CREB5/  ATF4/SMAD3/GLI2/FGF23/CREBBP/ |  | | | | | | | | | | | | | | | | | | | | | | | | | | | | | | | | | | | | | | | | | | | |
| virus-host interaction | 5.57E-11 | 10.25 | SMC2/RSAD2/KARS/C3/FBLN1/  C1QB/KRT18/CRTC3/ITGB3/C9orf69/CREBBP/ |  | | | | | | | | | | | | | | | | | | | | | | | | | | | | | | | | | | | | | | | | | | | |
| response to drug | 6.90E-11 | 10.16 | TXNIP/AQP7/TXNRD1/GSTT1/  INHBA/TGFB2/CPT1A/ |  | | | | | | | | | | | | | | | | | | | | | | | | | | | | | | | | | | | | | | | | | | | |
| negative regulation of  apoptotic process | 7.04E-11 | 10.15 | HIGD1A/IFIT3/RRN3/PRNP/IER3/  CLU/KRT18/CBL/GLI3/SMAD3/ |  | | | | | | | | | | | | | | | | | | | | | | | | | | | | | | | | | | | | | | | | | | | |
| cellular protein  metabolic process | 8.81E-11 | 10.05 | F5/GRPEL1/TIMM17A/SPCS2/  IGFBP5/PAPPA/ACTB/PLAU/ATF4/  MUC21/EDEM2/ |  | | | | | | | | | | | | | | | | | | | | | | | | | | | | | | | | | | | | | | | | | | | |
| cellular component movement | 1.62E-10 | 9.79 | ACTG1/CD97/ACTB/PXN/PLAU/  CERCAM/ |  | | | | | | | | | | | | | | | | | | | | | | | | | | | | | | | | | | | | | | | | | | | |
| fatty acid beta-oxidation | 3.97E-10 | 9.40 | CPT1A/ |  | | | | | | | | | | | | | | | | | | | | | | | | | | | | | | | | | | | | | | | | | | | |
| transcription initiation from  RNA polymerase II promoter | 4.42E-10 | 9.35 | NR1D2/TAF7/TEAD3/SMAD3/  CREBBP/ |  | | | | | | | | | | | | | | | | | | | | | | | | | | | | | | | | | | | | | | | | | | | |
| oxidation-reduction process | 6.83E-10 | 9.17 | UQCRB/COQ7/ |  | | | | | | | | | | | | | | | | | | | | | | | | | | | | | | | | | | | | | | | | | | | |
| cell proliferation | 7.93E-10 | 9.10 | RRN3/BST2/TXNRD1/WDR12/VTI1B/  PRG4/ELN/TGFB2/CD276/TGFBI/  CSPG4/GLI2/ |  | | | | | | | | | | | | | | | | | | | | | | | | | | | | | | | | | | | | | | | | | | | |
| S phase of mitotic cell cycle | 9.06E-10 | 9.04 | C6/SMC2/PSME2/PSMC5/C3/ |  | | | | | | | | | | | | | | | | | | | | | | | | | | | | | | | | | | | | | | | | | | | |
| antigen processing and  presentation of exogenous  peptide antigen via MHC class I | 1.07E-09 | 8.97 | C6/SMC2/PSME2/PSMC5/ |  | | | | | | | | | | | | | | | | | | | | | | | | | | | | | | | | | | | | | | | | | | | |
| extracellular matrix  disassembly | 1.07E-09 | 8.97 | COL1A1/COL3A1/COL1A2/ |  | | | | | | | | | | | | | | | | | | | | | | | | | | | | | | | | | | | | | | | | | | | |
| positive regulation of  ubiquitin-protein ligase  activity involved in  mitotic cell cycle | 1.20E-09 | 8.92 | C6/SMC2/PSME2/PSMC5/ |  | | | | | | | | | | | | | | | | | | | | | | | | | | | | | | | | | | | | | | | | | | | |
| ATP synthesis coupled  proton transport | 1.36E-09 | 8.87 | ATP5L2/ATP5O/ATP5G1/ |  | | | | | | | | | | | | | | | | | | | | | | | | | | | | | | | | | | | | | | | | | | | |
| collagen catabolic process | 1.53E-09 | 8.81 | COL1A1/COL3A1/COL1A2/  ADAMTS2/ |  | | | | | | | | | | | | | | | | | | | | | | | | | | | | | | | | | | | | | | | | | | | |
| transcription, DNA-dependent | 1.91E-09 | 8.72 | F5/C6/MLF1/ZNF844/NR1D2/ZNF404/  SLIRP/ZNF189/TXNIP/SOHLH2/  TCEAL8/ZNF230/ZNF615/KDM3A/  GTF3C6/ZNF675/ZNF649/RCBTB1/  C3/FSTL3/VGLL3/SEBOX/ZNF469/  C1QB/ELF4/GLI3/FOXO6/TP53INP2/  NFIC/CRTC3/CEBPE/TRAF7/BCL9L/  ZMIZ1/NFIX/SMAD3/GLI2/MAF/ |  | | | | | | | | | | | | | | | | | | | | | | | | | | | | | | | | | | | | | | | | | | | |
| M phase of mitotic cell cycle | 2.72E-09 | 8.57 | C6/SMC2/SKA2/PSME2/PSMC5/  ARPP19/C3/ |  | | | | | | | | | | | | | | | | | | | | | | | | | | | | | | | | | | | | | | | | | | | |
| antigen processing and  presentation of  exogenous peptide antigen  via MHC class I, TAP-dependent | 3.14E-09 | 8.50 | C6/SMC2/PSME2/PSMC5/ |  | | | | | | | | | | | | | | | | | | | | | | | | | | | | | | | | | | | | | | | | | | | |
| regulation of ubiquitin-protein  ligase activity involved  in mitotic cell cycle | 3.14E-09 | 8.50 | C6/SMC2/PSME2/PSMC5/ |  | | | | | | | | | | | | | | | | | | | | | | | | | | | | | | | | | | | | | | | | | | | |
| RNA metabolic process | 3.48E-09 | 8.46 | C6/SMC2/PSME2/PSMC5/ |  | | | | | | | | | | | | | | | | | | | | | | | | | | | | | | | | | | | | | | | | | | | |
| positive regulation of protein  kinase B signaling cascade | 4.52E-09 | 8.34 | HAX1/IGFBP5/C1QTNF1/HBEGF/  C1QB/CCR7/ |  | | | | | | | | | | | | | | | | | | | | | | | | | | | | | | | | | | | | | | | | | | | |
| negative regulation of angiogenesis | 6.12E-09 | 8.21 | RGCC/FOXO6/ |  | | | | | | | | | | | | | | | | | | | | | | | | | | | | | | | | | | | | | | | | | | | |
| antigen processing and presentation  of peptide antigen via MHC class I | 6.88E-09 | 8.16 | C6/SMC2/PSME2/PSMC5/ |  | | | | | | | | | | | | | | | | | | | | | | | | | | | | | | | | | | | | | | | | | | | |
| regulation of the force  of heart contraction | 7.20E-09 | 8.14 | PLN/ |  | | | | | | | | | | | | | | | | | | | | | | | | | | | | | | | | | | | | | | | | | | | |
| ossification | 9.24E-09 | 8.03 | RSAD2/C3/FSTL3/ECM1/SOST/  PDLIM7/CLEC3B/ |  | | | | | | | | | | | | | | | | | | | | | | | | | | | | | | | | | | | | | | | | | | | |
| anaphase-promoting complex-dependent  proteasomal ubiquitin-dependent  protein catabolic process | 9.54E-09 | 8.02 | C6/SMC2/PSME2/PSMC5/ |  | | | | | | | | | | | | | | | | | | | | | | | | | | | | | | | | | | | | | | | | | | | |
| collagen biosynthetic process | 1.11E-08 | 7.95 | COL1A1/COL3A1 |  | | | | | | | | | | | | | | | | | | | | | | | | | | | | | | | | | | | | | | | | | | | |
| positive regulation of  epithelial to mesenchymal transition | 1.40E-08 | 7.85 | RGCC/COL1A1/TGFB2/BCL9L/  SMAD3/ |  | | | | | | | | | | | | | | | | | | | | | | | | | | | | | | | | | | | | | | | | | | | |
| negative regulation of ubiquitin  -protein ligase activity involved  in mitotic cell cycle | 1.70E-08 | 7.77 | C6/SMC2/PSME2/PSMC5/ |  | | | | | | | | | | | | | | | | | | | | | | | | | | | | | | | | | | | | | | | | | | | |
| regulation of transcription,  DNA-dependent | 2.04E-08 | 7.69 | F5/ZNF844/NR1D2/ZNF404/ZRANB2/  SLIRP/ZNF880/SOHLH2/TTF1/  TCEAL8/ZNF230/ZNF615/TRIP4/  ZNF675/ZNF649/RCBTB1/C3/VGLL3/  ZNF469/NEO1/ELF4/CBL/ATF4/  TRAF7/SMAD3/GLI2/MAF/CREBBP/ |  | | | | | | | | | | | | | | | | | | | | | | | | | | | | | | | | | | | | | | | | | | | |
| positive regulation of  cell migration | 2.36E-08 | 7.63 | COL1A1/HBEGF/NTRK3/SMAD3/ |  | | | | | | | | | | | | | | | | | | | | | | | | | | | | | | | | | | | | | | | | | | | |
| DNA damage response, signal  transduction by p53 class  mediator resulting in cell  cycle arrest | 2.72E-08 | 7.56 | C6/SMC2/PSME2/PSMC5/RBM38/ |  | | | | | | | | | | | | | | | | | | | | | | | | | | | | | | | | | | | | | | | | | | | |
| response to virus | 2.78E-08 | 7.56 | RSAD2/IFIT3/BST2/C3/CLU/IFITM3/ |  | | | | | | | | | | | | | | | | | | | | | | | | | | | | | | | | | | | | | | | | | | | |
| protein folding | 3.09E-08 | 7.51 | CLGN/PPIL1/GRPEL1/QSOX1/ACTB/  ITGB3/EDEM2/ |  | | | | | | | | | | | | | | | | | | | | | | | | | | | | | | | | | | | | | | | | | | | |
| leukocyte migration | 4.04E-08 | 7.39 | COL1A1/COL1A2/SIRPA/ITGB3/ |  | | | | | | | | | | | | | | | | | | | | | | | | | | | | | | | | | | | | | | | | | | | |
| mitotic anaphase | 5.50E-08 | 7.26 | C6/SMC2/SKA2/PSME2/PSMC5/C3/ |  | | | | | | | | | | | | | | | | | | | | | | | | | | | | | | | | | | | | | | | | | | | |
| mRNA metabolic process | 5.60E-08 | 7.25 | C6/SMC2/PSME2/PSMC5/ |  | | | | | | | | | | | | | | | | | | | | | | | | | | | | | | | | | | | | | | | | | | | |
| translation | 7.70E-08 | 7.11 | F5/MRPL15/RPS4XP21/SPCS2/ |  | | | | | | | | | | | | | | | | | | | | | | | | | | | | | | | | | | | | | | | | | | | |
| protein polyubiquitination | 1.05E-07 | 6.98 | C6/SMC2/PSME2/PSMC5/ |  | | | | | | | | | | | | | | | | | | | | | | | | | | | | | | | | | | | | | | | | | | | |
| ureteric bud development | 1.13E-07 | 6.95 | NPNT/GDF11/SMAD3/ |  | | | | | | | | | | | | | | | | | | | | | | | | | | | | | | | | | | | | | | | | | | | |
| integrin-mediated  signaling pathway | 1.23E-07 | 6.91 | COL3A1/PXN/ITGB3/ |  | | | | | | | | | | | | | | | | | | | | | | | | | | | | | | | | | | | | | | | | | | | |
| regulation of  glucose metabolic process | 1.33E-07 | 6.88 | IGFBP5/C1QTNF1/ |  | | | | | | | | | | | | | | | | | | | | | | | | | | | | | | | | | | | | | | | | | | | |
| negative regulation  of gene expression | 1.57E-07 | 6.80 | HEY2/SLIT3/ |  | | | | | | | | | | | | | | | | | | | | | | | | | | | | | | | | | | | | | | | | | | | |
| xenobiotic metabolic process | 1.63E-07 | 6.79 | FMO2/GLYAT/ |  | | | | | | | | | | | | | | | | | | | | | | | | | | | | | | | | | | | | | | | | | | | |
| cell cycle arrest | 1.92E-07 | 6.72 | MLF1/PRKAG1/PRNP/INHBA/TGFB2/RBM38/SMAD3/ |  | | | | | | | | | | | | | | | | | | | | | | | | | | | | | | | | | | | | | | | | | | | |
| G1/S transition  of mitotic cell cycle | 1.98E-07 | 6.70 | C6/SMC2/PSME2/PSMC5/C3/INHBA/ |  | | | | | | | | | | | | | | | | | | | | | | | | | | | | | | | | | | | | | | | | | | | |
| response to mechanical  stimulus | 2.07E-07 | 6.68 | TXNIP/ACTA1/SOST/PTGER4/GLI2/ |  | | | | | | | | | | | | | | | | | | | | | | | | | | | | | | | | | | | | | | | | | | | |
| positive regulation  of cell growth | 2.39E-07 | 6.62 | HBEGF/TGFB2/ |  | | | | | | | | | | | | | | | | | | | | | | | | | | | | | | | | | | | | | | | | | | | |
| protein transport | 2.66E-07 | 6.58 | NMD3/RAB21/RAB18/HLA-DMA/  SEC22B/COL1A1/PRAF2/ARL17A/  SCAMP2/MVP/AGAP1/ |  | | | | | | | | | | | | | | | | | | | | | | | | | | | | | | | | | | | | | | | | | | | |
| lung development | 2.69E-07 | 6.57 | ASAH1/FSTL3/ADAMTS2/GLI3/GLI2/ |  | | | | | | | | | | | | | | | | | | | | | | | | | | | | | | | | | | | | | | | | | | | |
| muscle organ development | 2.77E-07 | 6.56 | TAGLN/HBEGF/ |  | | | | | | | | | | | | | | | | | | | | | | | | | | | | | | | | | | | | | | | | | | | |
| cellular response to  lipopolysaccharide | 2.94E-07 | 6.53 | CEBPE/ |  | | | | | | | | | | | | | | | | | | | | | | | | | | | | | | | | | | | | | | | | | | | |
| odontogenesis | 3.32E-07 | 6.48 | COL1A2/INHBA/TGFB2/ |  | | | | | | | | | | | | | | | | | | | | | | | | | | | | | | | | | | | | | | | | | | | |
| defense response to virus | 3.53E-07 | 6.45 | IFI44L/RSAD2/IFIT3/IFIT5/BST2/C3/  IFITM3/ |  | | | | | | | | | | | | | | | | | | | | | | | | | | | | | | | | | | | | | | | | | | | |
| negative regulation of  canonical Wnt receptor  signaling pathway | 3.89E-07 | 6.41 | SOST/GLI3/ |  | | | | | | | | | | | | | | | | | | | | | | | | | | | | | | | | | | | | | | | | | | | |
| transmembrane transport | 4.36E-07 | 6.36 | ATP6V1D/PRKAR1A/CLCN3/HK2/  AQP7/VDAC3/PEX3/C3/MFSD7/ |  | | | | | | | | | | | | | | | | | | | | | | | | | | | | | | | | | | | | | | | | | | | |
| cellular response to  tumor necrosis factor | 4.78E-07 | 6.32 | NPNT/ |  | | | | | | | | | | | | | | | | | | | | | | | | | | | | | | | | | | | | | | | | | | | |
| cell cycle checkpoint | 4.88E-07 | 6.31 | C6/SMC2/PSME2/PSMC5/C3/ |  | | | | | | | | | | | | | | | | | | | | | | | | | | | | | | | | | | | | | | | | | | | |
| positive regulation o  f apoptotic process | 5.63E-07 | 6.25 | TXNIP/IER3/CLU/TGFB2/C1QB/ |  | | | | | | | | | | | | | | | | | | | | | | | | | | | | | | | | | | | | | | | | | | | |
| oxidative phosphorylation | 6.41E-07 | 6.19 | UQCRB/ |  | | | | | | | | | | | | | | | | | | | | | | | | | | | | | | | | | | | | | | | | | | | |
| positive regulation  of MAPK cascade | 7.02E-07 | 6.15 | C1QTNF1/TRAF7/ |  | | | | | | | | | | | | | | | | | | | | | | | | | | | | | | | | | | | | | | | | | | | |
| positive regulation  of angiogenesis | 7.87E-07 | 6.10 | C6/C3/ECM1/ |  | | | | | | | | | | | | | | | | | | | | | | | | | | | | | | | | | | | | | | | | | | | |
| osteoblast differentiation | 8.81E-07 | 6.06 | COL1A1/IGFBP5/TP53INP2/EPHA2/  GLI2/ |  | | | | | | | | | | | | | | | | | | | | | | | | | | | | | | | | | | | | | | | | | | | |
| cell-matrix adhesion | 9.32E-07 | 6.03 | COL3A1/NPNT/FBLN5/PXN/ITGB3/ |  | | | | | | | | | | | | | | | | | | | | | | | | | | | | | | | | | | | | | | | | | | | |
| cell-cell signaling | 9.52E-07 | 6.02 | BST2/INHBA/TGFB2/WISP1/CD97/ |  | | | | | | | | | | | | | | | | | | | | | | | | | | | | | | | | | | | | | | | | | | | |
| cytokine-mediated  signaling pathway | 1.01E-06 | 6.00 | LIFR/IFI27/HLA-DRB4/IFIT3/ZNF675/  EIF4E3/PLP2/IFITM3/ |  | | | | | | | | | | | | | | | | | | | | | | | | | | | | | | | | | | | | | | | | | | | |
| inner ear development | 1.08E-06 | 5.97 | HLA-DMA/C1QB/LRP10/MAF/ |  | | | | | | | | | | | | | | | | | | | | | | | | | | | | | | | | | | | | | | | | | | | |
| response to copper ion | 1.24E-06 | 5.91 | PRNP/SOD3/ |  | | | | | | | | | | | | | | | | | | | | | | | | | | | | | | | | | | | | | | | | | | | |
| cell-substrate  junction assembly | 1.53E-06 | 5.81 | TNS1/ITGB3/ |  | | | | | | | | | | | | | | | | | | | | | | | | | | | | | | | | | | | | | | | | | | | |
| elastic fiber assembly | 1.53E-06 | 5.81 | FBLN5/ |  | | | | | | | | | | | | | | | | | | | | | | | | | | | | | | | | | | | | | | | | | | | |
| regulation of cell growth | 1.54E-06 | 5.81 | IGFBP5/FBLN5/WISP1/CRIM1/ |  | | | | | | | | | | | | | | | | | | | | | | | | | | | | | | | | | | | | | | | | | | | |
| response to calcium ion | 1.66E-06 | 5.78 | TXNIP |  | | | | | | | | | | | | | | | | | | | | | | | | | | | | | | | | | | | | | | | | | | | |
| complement activation,  classical pathway | 1.85E-06 | 5.73 | C6/C3/CLU/SERPING1/C1QB/C1QC/ |  | | | | | | | | | | | | | | | | | | | | | | | | | | | | | | | | | | | | | | | | | | | |
| protein heterotrimerization | 2.02E-06 | 5.70 | COL1A1/COL1A2/C1QTNF1/ |  | | | | | | | | | | | | | | | | | | | | | | | | | | | | | | | | | | | | | | | | | | | |
| response to estradiol  stimulus | 2.51E-06 | 5.60 | TXNIP/NR2F2/ |  | | | | | | | | | | | | | | | | | | | | | | | | | | | | | | | | | | | | | | | | | | | |
| negative regulation of  cell migration | 3.20E-06 | 5.50 | BST2/IGFBP5/ |  | | | | | | | | | | | | | | | | | | | | | | | | | | | | | | | | | | | | | | | | | | | |
| response to wounding | 3.24E-06 | 5.49 | TGFB2/ |  | | | | | | | | | | | | | | | | | | | | | | | | | | | | | | | | | | | | | | | | | | | |
| cell-cell junction  organization | 3.71E-06 | 5.43 | ACTG1/TGFB2/ACTB/ |  | | | | | | | | | | | | | | | | | | | | | | | | | | | | | | | | | | | | | | | | | | | |
| positive regulation of  I-kappaB kinase/NF-kappaB  cascade | 5.20E-06 | 5.28 | TNFRSF19/BST2/C3/FLNA/ECM1/  SMAD3/CANT1/CCR7/ |  | | | | | | | | | | | | | | | | | | | | | | | | | | | | | | | | | | | | | | | | | | | |
| cell migration | 5.22E-06 | 5.28 | TGFB2/ |  | | | | | | | | | | | | | | | | | | | | | | | | | | | | | | | | | | | | | | | | | | | |
| cellular response to  organic cyclic compound | 5.27E-06 | 5.28 | IGFBP5/GLI2/ |  | | | | | | | | | | | | | | | | | | | | | | | | | | | | | | | | | | | | | | | | | | | |
| positive regulation of  cell-substrate adhesion | 5.27E-06 | 5.28 | NPNT/ |  | | | | | | | | | | | | | | | | | | | | | | | | | | | | | | | | | | | | | | | | | | | |
| muscle filament sliding | 5.53E-06 | 5.26 | ACTA1/C3/TPM2/ |  | | | | | | | | | | | | | | | | | | | | | | | | | | | | | | | | | | | | | | | | | | | |
| neutrophil chemotaxis | 5.53E-06 | 5.26 | TGFB2 |  | | | | | | | | | | | | | | | | | | | | | | | | | | | | | | | | | | | | | | | | | | | |
| glycosaminoglycan  metabolic process | 5.62E-06 | 5.25 | HSPG2/NAGLU/HS3ST3A1/CSPG4/ |  | | | | | | | | | | | | | | | | | | | | | | | | | | | | | | | | | | | | | | | | | | | |
| Notch signaling pathway | 5.62E-06 | 5.25 | WDR12/HEY2/CREBBP/ |  | | | | | | | | | | | | | | | | | | | | | | | | | | | | | | | | | | | | | | | | | | | |
| positive regulation of  cell proliferation | 5.72E-06 | 5.24 | LIFR/EGF/TGFB2/NTRK3/S1PR2/  C9orf69/ |  | | | | | | | | | | | | | | | | | | | | | | | | | | | | | | | | | | | | | | | | | | | |
| menstrual cycle phase | 6.29E-06 | 5.20 | TGFB2/ |  | | | | | | | | | | | | | | | | | | | | | | | | | | | | | | | | | | | | | | | | | | | |
| response to glucocorticoid  stimulus | 6.43E-06 | 5.19 | PAPPA/ |  | | | | | | | | | | | | | | | | | | | | | | | | | | | | | | | | | | | | | | | | | | | |
| positive regulation of  ERK1 and ERK2 cascade | 6.70E-06 | 5.17 | NPNT/FGF23/CCR7/ |  | | | | | | | | | | | | | | | | | | | | | | | | | | | | | | | | | | | | | | | | | | | |
| regulation of ventricular  cardiac muscle cell  action potential | 8.25E-06 | 5.08 | GPD1L/ |  | | | | | | | | | | | | | | | | | | | | | | | | | | | | | | | | | | | | | | | | | | | |
| glycerophospholipid  biosynthetic process | 8.97E-06 | 5.05 | GPD1L/PLA2G16/ |  | | | | | | | | | | | | | | | | | | | | | | | | | | | | | | | | | | | | | | | | | | | |
| brain development | 9.19E-06 | 5.04 | ACAT1/COX17/RAB18/HSPG2/FZD8/ |  | | | | | | | | | | | | | | | | | | | | | | | | | | | | | | | | | | | | | | | | | | | |
| ubiquitin-dependent protein  catabolic process | 9.19E-06 | 5.04 | C6/SMC2/ |  | | | | | | | | | | | | | | | | | | | | | | | | | | | | | | | | | | | | | | | | | | | |
| branched-chain amino  acid catabolic process | 9.87E-06 | 5.01 | ACAT1/HIBADH/ |  | | | | | | | | | | | | | | | | | | | | | | | | | | | | | | | | | | | | | | | | | | | |
| response to oxidative stress | 1.00E-05 | 5.00 | NDUFA1/NDUFA6/PRNP/ |  | | | | | | | | | | | | | | | | | | | | | | | | | | | | | | | | | | | | | | | | | | | |
| negative regulation of  endothelial cell proliferation | 1.12E-05 | 4.95 | RGCC/NR2F2/ |  | | | | | | | | | | | | | | | | | | | | | | | | | | | | | | | | | | | | | | | | | | | |
| response to progesterone  stimulus | 1.12E-05 | 4.95 | TXNIP/TGFB2/ |  | | | | | | | | | | | | | | | | | | | | | | | | | | | | | | | | | | | | | | | | | | | |
| cell death | 1.34E-05 | 4.87 | ASAH1/CLU/GRN/TGFB2/PFN1/ |  | | | | | | | | | | | | | | | | | | | | | | | | | | | | | | | | | | | | | | | | | | | |
| generation of precursor  metabolites and energy | 1.45E-05 | 4.84 | COX7C/COX17/AQP7/ |  | | | | | | | | | | | | | | | | | | | | | | | | | | | | | | | | | | | | | | | | | | | |
| protein homotetramerization | 1.45E-05 | 4.84 | C3/CDA/CRTC3/ |  | | | | | | | | | | | | | | | | | | | | | | | | | | | | | | | | | | | | | | | | | | | |
| cell growth | 1.45E-05 | 4.84 | ACTA1/TGFB2/ |  | | | | | | | | | | | | | | | | | | | | | | | | | | | | | | | | | | | | | | | | | | | |
| regulation of apoptotic  process | 1.67E-05 | 4.78 | C6/SMC2/PSME2/PSMC5/TRAF7/  DAP/ |  | | | | | | | | | | | | | | | | | | | | | | | | | | | | | | | | | | | | | | | | | | | |
| negative regulation  of cell growth | 1.71E-05 | 4.77 | BST2/INHBA/SLIT3/TGFB2/CDA/  SMAD3/ |  | | | | | | | | | | | | | | | | | | | | | | | | | | | | | | | | | | | | | | | | | | | |
| phospholipid metabolic  process | 1.80E-05 | 4.74 | GPD1L/PLA2G16/PIK3R3/C3/ |  | | | | | | | | | | | | | | | | | | | | | | | | | | | | | | | | | | | | | | | | | | | |
| electron transport chain | 1.85E-05 | 4.73 | HIGD1A/TXNRD1/CYB5B/FADS3/ |  | | | | | | | | | | | | | | | | | | | | | | | | | | | | | | | | | | | | | | | | | | | |
| response to organic  cyclic compound | 1.85E-05 | 4.73 | ACAT1/GSTT1/CPT1A/ |  | | | | | | | | | | | | | | | | | | | | | | | | | | | | | | | | | | | | | | | | | | | |
| positive regulation of  gene expression | 2.08E-05 | 4.68 | PRKAG1/C3/TGFB2/NTRK3/ |  | | | | | | | | | | | | | | | | | | | | | | | | | | | | | | | | | | | | | | | | | | | |
| ventricular cardiac  muscle tissue morphogenesis | 2.12E-05 | 4.67 | C3/ |  | | | | | | | | | | | | | | | | | | | | | | | | | | | | | | | | | | | | | | | | | | | |
| cellular response to  amino acid stimulus | 2.15E-05 | 4.67 | COL1A1/COL3A1/COL1A2/ |  | | | | | | | | | | | | | | | | | | | | | | | | | | | | | | | | | | | | | | | | | | | |
| gluconeogenesis | 2.31E-05 | 4.64 | PGK1/ATF4/ |  | | | | | | | | | | | | | | | | | | | | | | | | | | | | | | | | | | | | | | | | | | | |
| transcription from RNA  polymerase II promoter | 2.46E-05 | 4.61 | HSF2/RNMT/TRIP4/PSMC5/TAF7/C3/  MSC/NFIC/CREB5/ATF4/NFIX/MAF/ |  | | | | | | | | | | | | | | | | | | | | | | | | | | | | | | | | | | | | | | | | | | | |
| negative regulation of  transforming growth factor  beta receptor signaling pathway | 2.60E-05 | 4.59 | BCL9L/SMAD3/ |  | | | | | | | | | | | | | | | | | | | | | | | | | | | | | | | | | | | | | | | | | | | |
| complement activation | 2.76E-05 | 4.56 | C6/RGCC/C3/CLU/C1QB/C1QC/ |  | | | | | | | | | | | | | | | | | | | | | | | | | | | | | | | | | | | | | | | | | | | |
| positive regulation of  protein phosphorylation | 2.80E-05 | 4.55 | LRRN3/RRN3/ITLN1/C3/ITGB3/ |  | | | | | | | | | | | | | | | | | | | | | | | | | | | | | | | | | | | | | | | | | | | |
| negative regulation of  smooth muscle cell migration | 2.89E-05 | 4.54 | IGFBP5/ |  | | | | | | | | | | | | | | | | | | | | | | | | | | | | | | | | | | | | | | | | | | | |
| type B pancreatic  cell proliferation | 3.04E-05 | 4.52 | IGFBP5/ |  | | | | | | | | | | | | | | | | | | | | | | | | | | | | | | | | | | | | | | | | | | | |
| ATP biosynthetic process | 3.23E-05 | 4.49 | ATP5O/ |  | | | | | | | | | | | | | | | | | | | | | | | | | | | | | | | | | | | | | | | | | | | |
| viral reproduction | 3.25E-05 | 4.49 | C6/SMC2/RNMT/PSME2/PSMC5/ |  | | | | | | | | | | | | | | | | | | | | | | | | | | | | | | | | | | | | | | | | | | | |
| response to ethanol | 3.76E-05 | 4.42 | NTRK3/ |  | | | | | | | | | | | | | | | | | | | | | | | | | | | | | | | | | | | | | | | | | | | |
| response to cAMP | 4.43E-05 | 4.35 | COL1A1/ |  | | | | | | | | | | | | | | | | | | | | | | | | | | | | | | | | | | | | | | | | | | | |
| in utero embryonic development | 4.46E-05 | 4.35 | C6/RRN3/COQ7/GLI3/ZMIZ1/SMAD3/GLI2/ |  | | | | | | | | | | | | | | | | | | | | | | | | | | | | | | | | | | | | | | | | | | | |
| positive regulation of  osteoblast differentiation | 4.46E-05 | 4.35 | NPNT/CD276/GLI3/PDLIM7/ |  | | | | | | | | | | | | | | | | | | | | | | | | | | | | | | | | | | | | | | | | | | | |
| sarcomere organization | 4.56E-05 | 4.34 | PRKAR1A/C3/ACTG1/ |  | | | | | | | | | | | | | | | | | | | | | | | | | | | | | | | | | | | | | | | | | | | |
| response to lipopolysaccharide | 4.84E-05 | 4.32 | PTGER4/CCR7/ |  | | | | | | | | | | | | | | | | | | | | | | | | | | | | | | | | | | | | | | | | | | | |
| response to hydrogen peroxide | 5.54E-05 | 4.26 | TXNIP/COL1A1/ |  | | | | | | | | | | | | | | | | | | | | | | | | | | | | | | | | | | | | | | | | | | | |
| antigen processing and presentation  of peptide or polysaccharide  antigen via MHC class II | 5.58E-05 | 4.25 | HLA-DMA/ |  | | | | | | | | | | | | | | | | | | | | | | | | | | | | | | | | | | | | | | | | | | | |
| extracellular fibril organization | 5.58E-05 | 4.25 | COL3A1/ |  | | | | | | | | | | | | | | | | | | | | | | | | | | | | | | | | | | | | | | | | | | | |
| biological_process | 5.64E-05 | 4.25 | HMGN3/GHITM/EGF/CCDC47/  TMEM106C/MINOS1/PRMT3/C3/  CD248/SEBOX/MN1/LPP/IRF2BPL/ |  | | | | | | | | | | | | | | | | | | | | | | | | | | | | | | | | | | | | | | | | | | | |
| vasculature development | 6.31E-05 | 4.20 | FZD8/ |  | | | | | | | | | | | | | | | | | | | | | | | | | | | | | | | | | | | | | | | | | | | |
| cellular response to cAMP | 6.31E-05 | 4.20 | IGFBP5/ |  | | | | | | | | | | | | | | | | | | | | | | | | | | | | | | | | | | | | | | | | | | | |
| response to cold | 6.49E-05 | 4.19 | CIRBP/ |  | | | | | | | | | | | | | | | | | | | | | | | | | | | | | | | | | | | | | | | | | | | |
| nervous system development | 6.83E-05 | 4.17 | INHBA/NAGLU/GDF11/ENC1/CRIM1/ |  | | | | | | | | | | | | | | | | | | | | | | | | | | | | | | | | | | | | | | | | | | | |
| response to peptide  hormone stimulus | 6.88E-05 | 4.16 | COL1A1/ |  | | | | | | | | | | | | | | | | | | | | | | | | | | | | | | | | | | | | | | | | | | | |
| protein oligomerization | 6.88E-05 | 4.16 | STOML2/ |  | | | | | | | | | | | | | | | | | | | | | | | | | | | | | | | | | | | | | | | | | | | |
| regulation of blood pressure | 7.38E-05 | 4.13 | COL1A2/ |  | | | | | | | | | | | | | | | | | | | | | | | | | | | | | | | | | | | | | | | | | | | |
| cell surface receptor  signaling pathway | 7.42E-05 | 4.13 | LIFR/TDP2/INHBA/CDA/CD97/CBL/ |  | | | | | | | | | | | | | | | | | | | | | | | | | | | | | | | | | | | | | | | | | | | |
| Wnt receptor signaling pathway | 7.42E-05 | 4.13 | WISP1/SOST/ |  | | | | | | | | | | | | | | | | | | | | | | | | | | | | | | | | | | | | | | | | | | | |
| aging | 7.85E-05 | 4.11 | MGEA5/SCAP/ |  | | | | | | | | | | | | | | | | | | | | | | | | | | | | | | | | | | | | | | | | | | | |
| cellular response to transforming  growth factor beta stimulus | 8.33E-05 | 4.0 | COL1A1/C3/CLEC3B/ |  | | | | | | | | | | | | | | | | | | | | | | | | | | | | | | | | | | | | | | | | | | | |
| transcription from RNA  polymerase I promoter | 8.59E-05 | 4.07 | TTF1/RRN3/ |  | | | | | | | | | | | | | | | | | | | | | | | | | | | | | | | | | | | | | | | | | | | |
| transcription initiation  from RNA polymerase I promoter | 8.59E-05 | 4.07 | TTF1/RRN3/ |  | | | | | | | | | | | | | | | | | | | | | | | | | | | | | | | | | | | | | | | | | | | |
| transport | 8.80E-05 | 4.06 | CLCN3/SMAD3/ |  | | | | | | | | | | | | | | | | | | | | | | | | | | | | | | | | | | | | | | | | | | | |
| intramembranous ossification | 8.81E-05 | 4.05 | COL1A1/MN1/ |  | | | | | | | | | | | | | | | | | | | | | | | | | | | | | | | | | | | | | | | | | | | |
| negative regulation  of inflammatory response | 1.08E-04 | 3.97 | IER3/CD276/PTGER4/SMAD3/ |  | | | | | | | | | | | | | | | | | | | | | | | | | | | | | | | | | | | | | | | | | | | |
| positive regulation of  NF-kappaB transcription  factor activity | 1.09E-04 | 3.96 | CLU/ |  | | | | | | | | | | | | | | | | | | | | | | | | | | | | | | | | | | | | | | | | | | | |
| positive regulation of  cytokine secretion | 1.15E-04 | 3.94 | RGCC/PTGER4/ |  | | | | | | | | | | | | | | | | | | | | | | | | | | | | | | | | | | | | | | | | | | | |
| eye development | 1.33E-04 | 3.88 | RAB18/TGFB2/ |  | | | | | | | | | | | | | | | | | | | | | | | | | | | | | | | | | | | | | | | | | | | |
| negative regulation of  osteoclast differentiation | 1.33E-04 | 3.88 | ZNF675/FSTL3/ |  | | | | | | | | | | | | | | | | | | | | | | | | | | | | | | | | | | | | | | | | | | | |
| superoxide metabolic process | 1.33E-04 | 3.88 | SOD3/ |  | | | | | | | | | | | | | | | | | | | | | | | | | | | | | | | | | | | | | | | | | | | |
| negative regulation of  protein phosphorylation | 1.33E-04 | 3.87 | PRNP/NTRK3/SMAD3/ |  | | | | | | | | | | | | | | | | | | | | | | | | | | | | | | | | | | | | | | | | | | | |
| heart morphogenesis | 1.33E-04 | 3.87 | C3/TGFB2/ZMIZ1/ |  | | | | | | | | | | | | | | | | | | | | | | | | | | | | | | | | | | | | | | | | | | | |
| cell junction assembly | 1.35E-04 | 3.87 | FLNA/ACTG1/ACTB/PXN/ |  | | | | | | | | | | | | | | | | | | | | | | | | | | | | | | | | | | | | | | | | | | | |
| wound healing | 1.37E-04 | 3.86 | COL3A1/TGFB2/ITGB3/SMAD3/ |  | | | | | | | | | | | | | | | | | | | | | | | | | | | | | | | | | | | | | | | | | | | |
| blood circulation | 1.53E-04 | 3.81 | F5/PLN/ELN/SERPING1/MEOX2/ |  | | | | | | | | | | | | | | | | | | | | | | | | | | | | | | | | | | | | | | | | | | | |
| interferon-gamma-mediated  signaling pathway | 1.59E-04 | 3.80 | HLA-DRB4/ |  | | | | | | | | | | | | | | | | | | | | | | | | | | | | | | | | | | | | | | | | | | | |
| negative regulation of  cytokine secretion | 1.63E-04 | 3.79 | RGCC/C3/PTGER4/ |  | | | | | | | | | | | | | | | | | | | | | | | | | | | | | | | | | | | | | | | | | | | |
| negative regulation of  JUN kinase activity | 1.63E-04 | 3.79 | ZNF675/ |  | | | | | | | | | | | | | | | | | | | | | | | | | | | | | | | | | | | | | | | | | | | |
| regulation of  insulin secretion | 1.76E-04 | 3.76 | PRKAR1A/RAPGEF4/SPCS2/CPT1A/ |  | | | | | | | | | | | | | | | | | | | | | | | | | | | | | | | | | | | | | | | | | | | |
| induction of apoptosis | 1.86E-04 | 3.73 | TNFRSF19/INHBA/TGFB2/PHLDA1/  SMAD3/DAP/ |  | | | | | | | | | | | | | | | | | | | | | | | | | | | | | | | | | | | | | | | | | | | |
| leukocyte cell-cell adhesion | 1.97E-04 | 3.71 | CERCAM/ |  | | | | | | | | | | | | | | | | | | | | | | | | | | | | | | | | | | | | | | | | | | | |
| response to starvation | 1.97E-04 | 3.71 | ACAT1/ |  | | | | | | | | | | | | | | | | | | | | | | | | | | | | | | | | | | | | | | | | | | | |
| toxin metabolic process | 1.99E-04 | 3.70 | FMO2/AS3MT/ |  | | | | | | | | | | | | | | | | | | | | | | | | | | | | | | | | | | | | | | | | | | | |
| pulmonary valve morphogenesis | 1.99E-04 | 3.70 | HEY2/ |  | | | | | | | | | | | | | | | | | | | | | | | | | | | | | | | | | | | | | | | | | | | |
| negative regulation of  transcription, DNA-dependent | 2.30E-04 | 3.64 | C6/PSMC5/TAF7/HEY2/NR2F2/GLI3/DAP/ |  | | | | | | | | | | | | | | | | | | | | | | | | | | | | | | | | | | | | | | | | | | | |
| epidermal growth factor  receptor signaling pathway | 2.33E-04 | 3.63 | PRKAR1A/EGF/HBEGF/CBL/PXN/  FGF23/ |  | | | | | | | | | | | | | | | | | | | | | | | | | | | | | | | | | | | | | | | | | | | |
| response to stress | 2.43E-04 | 3.62 | HSF2/HIGD1A/HSPB6/ |  | | | | | | | | | | | | | | | | | | | | | | | | | | | | | | | | | | | | | | | | | | | |
| cellular response to  mechanical stimulus | 2.44E-04 | 3.61 | COL1A1/PTGER4/ |  | | | | | | | | | | | | | | | | | | | | | | | | | | | | | | | | | | | | | | | | | | | |
| regulation of  translational initiation | 2.54E-04 | 3.60 | F5/MTIF3/ |  | | | | | | | | | | | | | | | | | | | | | | | | | | | | | | | | | | | | | | | | | | | |
| embryo implantation | 2.54E-04 | 3.60 | GRN/FBLN1/ |  | | | | | | | | | | | | | | | | | | | | | | | | | | | | | | | | | | | | | | | | | | | |
| protein ubiquitination | 2.54E-04 | 3.60 | ANAPC16/PJA2/CBL/TRAF7/ENC1/ |  | | | | | | | | | | | | | | | | | | | | | | | | | | | | | | | | | | | | | | | | | | | |
| negative regulation of  chondrocyte differentiation | 2.56E-04 | 3.59 | LTBP3/GLI2/ |  | | | | | | | | | | | | | | | | | | | | | | | | | | | | | | | | | | | | | | | | | | | |
| bone remodeling | 2.56E-04 | 3.59 | LTBP3/EPHA2/ |  | | | | | | | | | | | | | | | | | | | | | | | | | | | | | | | | | | | | | | | | | | | |
| negative regulation of  macrophage derived foam  cell differentiation | 2.56E-04 | 3.59 | ITGB3/ |  | | | | | | | | | | | | | | | | | | | | | | | | | | | | | | | | | | | | | | | | | | | |
| macrophage differentiation | 2.56E-04 | 3.59 | CEBPE/ |  | | | | | | | | | | | | | | | | | | | | | | | | | | | | | | | | | | | | | | | | | | | |
| response to glucose  stimulus | 2.84E-04 | 3.55 | TXNIP/ |  | | | | | | | | | | | | | | | | | | | | | | | | | | | | | | | | | | | | | | | | | | | |
| anatomical structure  morphogenesis | 2.85E-04 | 3.55 | IER3/KRT18/EN1/ |  | | | | | | | | | | | | | | | | | | | | | | | | | | | | | | | | | | | | | | | | | | | |
| multicellular organismal  development | 3.04E-04 | 3.52 | MBNL3/SOHLH2/BST2/SEBOX/  MEOX2/PDLIM7/ENC1/EPHA2/  PLXNA1/ |  | | | | | | | | | | | | | | | | | | | | | | | | | | | | | | | | | | | | | | | | | | | |
| protein phosphorylation | 3.46E-04 | 3.46 | PRKAG1/PRKG1/TBCK/TGFB2/  STK19/DAP/ |  | | | | | | | | | | | | | | | | | | | | | | | | | | | | | | | | | | | | | | | | | | | |
| osteoclast differentiation | 3.49E-04 | 3.46 | EPHA2/ |  | | | | | | | | | | | | | | | | | | | | | | | | | | | | | | | | | | | | | | | | | | | |
| cellular response to heat | 3.49E-04 | 3.46 | XYLT1/ |  | | | | | | | | | | | | | | | | | | | | | | | | | | | | | | | | | | | | | | | | | | | |
| negative regulation of  neuron apoptotic process | 3.54E-04 | 3.45 | LGMN/ |  | | | | | | | | | | | | | | | | | | | | | | | | | | | | | | | | | | | | | | | | | | | |
| protein targeting to  mitochondrion | 3.68E-04 | 3.43 | GRPEL1/TIMM17A/ |  | | | | | | | | | | | | | | | | | | | | | | | | | | | | | | | | | | | | | | | | | | | |
| fatty acid biosynthetic  process | 3.68E-04 | 3.43 | PRKAG1/FADS3/ |  | | | | | | | | | | | | | | | | | | | | | | | | | | | | | | | | | | | | | | | | | | | |
| response to hormone  stimulus | 3.75E-04 | 3.43 | ACAT1/ |  | | | | | | | | | | | | | | | | | | | | | | | | | | | | | | | | | | | | | | | | | | | |
| SMAD protein import  into nucleus | 3.84E-04 | 3.42 | TGFB2/ |  | | | | | | | | | | | | | | | | | | | | | | | | | | | | | | | | | | | | | | | | | | | |
| response to selenium  ion | 3.84E-04 | 3.42 | TXNRD1/ |  | | | | | | | | | | | | | | | | | | | | | | | | | | | | | | | | | | | | | | | | | | | |
| actin crosslink formation | 3.84E-04 | 3.42 | FLNA/ |  | | | | | | | | | | | | | | | | | | | | | | | | | | | | | | | | | | | | | | | | | | | |
| Notch signaling involved  in heart development | 3.84E-04 | 3.42 | HEY2/ |  | | | | | | | | | | | | | | | | | | | | | | | | | | | | | | | | | | | | | | | | | | | |
| face morphogenesis | 4.05E-04 | 3.39 | COL1A1/TGFB2/ |  | | | | | | | | | | | | | | | | | | | | | | | | | | | | | | | | | | | | | | | | | | | |
| lysosome organization | 4.05E-04 | 3.39 | NAGLU/ |  | | | | | | | | | | | | | | | | | | | | | | | | | | | | | | | | | | | | | | | | | | | |
| regulation of heart rate | 4.63E-04 | 3.33 | GPD1L/C3 |  | | | | | | | | | | | | | | | | | | | | | | | | | | | | | | | | | | | | | | | | | | | |
| endosome organization | 4.63E-04 | 3.33 | C3/ |  | | | | | | | | | | | | | | | | | | | | | | | | | | | | | | | | | | | | | | | | | | | |
| phosphatidic acid  biosynthetic process | 5.04E-04 | 3.30 | GPD1L/ |  | | | | | | | | | | | | | | | | | | | | | | | | | | | | | | | | | | | | | | | | | | | |
| regulation of cell  migration | 5.05E-04 | 3.30 | ITGB3/ |  | | | | | | | | | | | | | | | | | | | | | | | | | | | | | | | | | | | | | | | | | | | |
| intracellular signal  transduction | 5.20E-04 | 3.28 | F5/PLCL2/PRKAR1A/IGFBP5/CSPG4/TNS1/ |  | | | | | | | | | | | | | | | | | | | | | | | | | | | | | | | | | | | | | | | | | | | |
| RNA splicing | 5.26E-04 | 3.28 | MBNL3/ZRANB2/HNRNPH2/PPP4R2/HNRNPA2B1/BCAS2/  C3/C1QB/PTBP1/RBM38/ |  | | | | | | | | | | | | | | | | | | | | | | | | | | | | | | | | | | | | | | | | | | | |
| protein homooligomerization | 5.29E-04 | 3.28 | ACAT1/PRNP/C1QTNF1/SCUBE3/  CPT1A/ |  | | | | | | | | | | | | | | | | | | | | | | | | | | | | | | | | | | | | | | | | | | | |
| cell differentiation | 5.34E-04 | 3.27 | SOHLH2/NPNT/INHBA/SEBOX/  PAPPA/TMEM176B/  RBM38/PDLIM7/FGF23/ |  | | | | | | | | | | | | | | | | | | | | | | | | | | | | | | | | | | | | | | | | | | | |
| positive regulation of  smooth muscle cell  proliferation | 5.40E-04 | 3.27 | HBEGF/ |  | | | | | | | | | | | | | | | | | | | | | | | | | | | | | | | | | | | | | | | | | | | |
| palate development | 5.74E-04 | 3.24 | INHBA/MEOX2/MSC/GLI3/ |  | | | | | | | | | | | | | | | | | | | | | | | | | | | | | | | | | | | | | | | | | | | |
| negative regulation of  systemic arterial  blood pressure | 5.79E-04 | 3.24 | IER3/ |  | | | | | | | | | | | | | | | | | | | | | | | | | | | | | | | | | | | | | | | | | | | |
| negative regulation of  macrophage cytokine  production | 5.79E-04 | 3.24 | TGFB2/ |  | | | | | | | | | | | | | | | | | | | | | | | | | | | | | | | | | | | | | | | | | | | |
| ribosome disassembly | 5.79E-04 | 3.24 | MTIF3/ |  | | | | | | | | | | | | | | | | | | | | | | | | | | | | | | | | | | | | | | | | | | | |
| frontal suture  morphogenesis | 5.79E-04 | 3.24 | GLI3/ |  | | | | | | | | | | | | | | | | | | | | | | | | | | | | | | | | | | | | | | | | | | | |
| vascular smooth muscle  cell development | 5.79E-04 | 3.24 | HEY2/ |  | | | | | | | | | | | | | | | | | | | | | | | | | | | | | | | | | | | | | | | | | | | |
| protein complex assembly | 5.88E-04 | 3.23 | CLGN/ATPAF1/HLA-DMA/CREBBP/ |  | | | | | | | | | | | | | | | | | | | | | | | | | | | | | | | | | | | | | | | | | | | |
| positive regulation of  endothelial cell proliferation | 5.88E-04 | 3.23 | ECM1/ITGB3/ |  | | | | | | | | | | | | | | | | | | | | | | | | | | | | | | | | | | | | | | | | | | | |
| response to cytokine stimulus | 5.88E-04 | 3.23 | LIFR/COL3A1/ |  | | | | | | | | | | | | | | | | | | | | | | | | | | | | | | | | | | | | | | | | | | | |
| response to unfolded protein | 5.88E-04 | 3.23 | EDEM2/ |  | | | | | | | | | | | | | | | | | | | | | | | | | | | | | | | | | | | | | | | | | | | |
| cellular response to  organic substance | 6.04E-04 | 3.22 | C3/CLEC3B/ |  | | | | | | | | | | | | | | | | | | | | | | | | | | | | | | | | | | | | | | | | | | | |
| mitochondrion organization | 6.21E-04 | 3.21 | STOML2/ |  | | | | | | | | | | | | | | | | | | | | | | | | | | | | | | | | | | | | | | | | | | | |
| chaperone-mediated  protein folding | 6.68E-04 | 3.17 | CLU/ |  | | | | | | | | | | | | | | | | | | | | | | | | | | | | | | | | | | | | | | | | | | | |
| cellular membrane  organization | 7.32E-04 | 3.14 | C6/PRKAG1/ACTG1/ACTB/ |  | | | | | | | | | | | | | | | | | | | | | | | | | | | | | | | | | | | | | | | | | | | |
| regulation of heart  contraction | 7.60E-04 | 3.12 | PLN/HBEGF/ |  | | | | | | | | | | | | | | | | | | | | | | | | | | | | | | | | | | | | | | | | | | | |
| negative regulation of  BMP signaling pathway | 7.60E-04 | 3.12 | FSTL3/SOST/ |  | | | | | | | | | | | | | | | | | | | | | | | | | | | | | | | | | | | | | | | | | | | |
| negative regulation of  bone mineralization | 7.83E-04 | 3.11 | ECM1/LTBP3/FGF23/ |  | | | | | | | | | | | | | | | | | | | | | | | | | | | | | | | | | | | | | | | | | | | |
| positive regulation of  vascular endothelial growth  factor receptor signaling pathway | 7.83E-04 | 3.11 | ITGB3/ |  | | | | | | | | | | | | | | | | | | | | | | | | | | | | | | | | | | | | | | | | | | | |
| mitochondrial respiratory  chain complex I assembly | 7.83E-04 | 3.11 | NDUFAF4/ |  | | | | | | | | | | | | | | | | | | | | | | | | | | | | | | | | | | | | | | | | | | | |
| blood vessel morphogenesis | 7.83E-04 | 3.11 | NR2F2/ |  | | | | | | | | | | | | | | | | | | | | | | | | | | | | | | | | | | | | | | | | | | | |
| positive regulation of  epithelial cell proliferation | 7.88E-04 | 3.10 | GRN/ |  | | | | | | | | | | | | | | | | | | | | | | | | | | | | | | | | | | | | | | | | | | | |
| defense response | 8.45E-04 | 3.07 | INHBA/CEBPE/ |  | | | | | | | | | | | | | | | | | | | | | | | | | | | | | | | | | | | | | | | | | | | |
| cellular response to hypoxia | 9.38E-04 | 3.03 | RGCC/CREBBP/ |  | | | | | | | | | | | | | | | | | | | | | | | | | | | | | | | | | | | | | | | | | | | |
| positive regulation of  peptidyl-tyrosine  phosphorylation | 9.56E-04 | 3.02 | HAX1/CSPG4/ITGB3/ |  | | | | | | | | | | | | | | | | | | | | | | | | | | | | | | | | | | | | | | | | | | | |
| antigen processing and  presentation of exogenous  peptide antigen via MHC class II | 9.71E-04 | 3.01 | HLA-DRB4/HLA-DMA/LGMN/ |  | | | | | | | | | | | | | | | | | | | | | | | | | | | | | | | | | | | | | | | | | | | |
| positive regulation of  inflammatory response | 1.05E-03 | 2.98 | PTGER4/ |  | | | | | | | | | | | | | | | | | | | | | | | | | | | | | | | | | | | | | | | | | | | |
| adipose tissue development | 1.07E-03 | 2.97 | ACAT1/CREB5/ |  | | | | | | | | | | | | | | | | | | | | | | | | | | | | | | | | | | | | | | | | | | | |
| cAMP-mediated signaling | 1.07E-03 | 2.97 | RAPGEF4 |  | | | | | | | | | | | | | | | | | | | | | | | | | | | | | | | | | | | | | | | | | | | |
| negative regulation of  mitotic cell cycle | 1.07E-03 | 2.97 | SMAD3/ |  | | | | | | | | | | | | | | | | | | | | | | | | | | | | | | | | | | | | | | | | | | | |
| positive regulation of  catenin import into nucleus | 1.08E-03 | 2.97 | EGF/SMAD3/ |  | | | | | | | | | | | | | | | | | | | | | | | | | | | | | | | | | | | | | | | | | | | |
| placenta blood  vessel development | 1.08E-03 | 2.97 | NR2F2/ |  | | | | | | | | | | | | | | | | | | | | | | | | | | | | | | | | | | | | | | | | | | | |
| cardiac muscle contraction | 1.11E-03 | 2.95 | C3/ |  | | | | | | | | | | | | | | | | | | | | | | | | | | | | | | | | | | | | | | | | | | | |
| cell communication | 1.17E-03 | 2.93 | GJD2/ZCCHC14/ |  | | | | | | | | | | | | | | | | | | | | | | | | | | | | | | | | | | | | | | | | | | | |
| protein stabilization | 1.22E-03 | 2.92 | FLNA/CLU/SMAD3/ |  | | | | | | | | | | | | | | | | | | | | | | | | | | | | | | | | | | | | | | | | | | | |
| positive regulation of  phosphatidylinositol  3-kinase cascade | 1.22E-03 | 2.91 | HAX1/TGFB2/CBL/HCST/ |  | | | | | | | | | | | | | | | | | | | | | | | | | | | | | | | | | | | | | | | | | | | |
| response to axon injury | 1.24E-03 | 2.91 | TXNRD1/NTRK3/ |  | | | | | | | | | | | | | | | | | | | | | | | | | | | | | | | | | | | | | | | | | | | |
| negative regulation of  smooth muscle cell  proliferation | 1.24E-03 | 2.91 | IGFBP5/ |  | | | | | | | | | | | | | | | | | | | | | | | | | | | | | | | | | | | | | | | | | | | |
| cell cycle | 1.26E-03 | 2.90 | TXNIP/RCBTB1/KRT18/RBM38/  C9orf69/ |  | | | | | | | | | | | | | | | | | | | | | | | | | | | | | | | | | | | | | | | | | | | |
| mRNA processing | 1.28E-03 | 2.89 | MBNL3/ZRANB2/PPP4R2/  HNRNPA2B1/BCAS2/C3/  C1QB/PTBP1/RBM38/ |  | | | | | | | | | | | | | | | | | | | | | | | | | | | | | | | | | | | | | | | | | | | |
| activation of MAPK activity | 1.30E-03 | 2.89 | NTRK3/S1PR2/CSPG4/PXN/ |  | | | | | | | | | | | | | | | | | | | | | | | | | | | | | | | | | | | | | | | | | | | |
| glycosaminoglycan  biosynthetic process | 1.33E-03 | 2.88 | HSPG2/HS3ST3A1/XYLT1/ |  | | | | | | | | | | | | | | | | | | | | | | | | | | | | | | | | | | | | | | | | | | | |
| adherens junction organization | 1.33E-03 | 2.88 | ACTG1/ACTB/ |  | | | | | | | | | | | | | | | | | | | | | | | | | | | | | | | | | | | | | | | | | | | |
| mitochondrial electron  transport, ubiquinol  to cytochrome c | 1.40E-03 | 2.85 | UQCRB/UQCR10/ |  | | | | | | | | | | | | | | | | | | | | | | | | | | | | | | | | | | | | | | | | | | | |
| NADH metabolic process | 1.40E-03 | 2.85 | GPD1L/IDH3B/ |  | | | | | | | | | | | | | | | | | | | | | | | | | | | | | | | | | | | | | | | | | | | |
| negative regulation of  granulocyte differentiation | 1.40E-03 | 2.85 | C1QC/ |  | | | | | | | | | | | | | | | | | | | | | | | | | | | | | | | | | | | | | | | | | | | |
| negative regulation  of RNA splicing | 1.40E-03 | 2.85 | PTBP1/ |  | | | | | | | | | | | | | | | | | | | | | | | | | | | | | | | | | | | | | | | | | | | |
| regulation of binding | 1.40E-03 | 2.85 | SMAD3/ |  | | | | | | | | | | | | | | | | | | | | | | | | | | | | | | | | | | | | | | | | | | | |
| positive regulation of  sodium ion transport | 1.43E-03 | 2.84 | GPD1L/ |  | | | | | | | | | | | | | | | | | | | | | | | | | | | | | | | | | | | | | | | | | | | |
| positive regulation of  glucose import | 1.53E-03 | 2.81 | MGEA5/ARPP19/ITLN1/ |  | | | | | | | | | | | | | | | | | | | | | | | | | | | | | | | | | | | | | | | | | | | |
| branching morphogenesis  of an epithelial tube | 1.53E-03 | 2.81 | EGF/PXN/GLI2/ |  | | | | | | | | | | | | | | | | | | | | | | | | | | | | | | | | | | | | | | | | | | | |
| positive regulation  of mitosis | 1.53E-03 | 2.81 | EGF/RGCC/ |  | | | | | | | | | | | | | | | | | | | | | | | | | | | | | | | | | | | | | | | | | | | |
| gonad development | 1.53E-03 | 2.81 | FZD8/ |  | | | | | | | | | | | | | | | | | | | | | | | | | | | | | | | | | | | | | | | | | | | |
| negative regulation of  protein kinase activity | 1.54E-03 | 2.81 | PRKAR1A/TAF7/ |  | | | | | | | | | | | | | | | | | | | | | | | | | | | | | | | | | | | | | | | | | | | |
| negative regulation of  NF-kappaB transcription  factor activity | 1.54E-03 | 2.81 | ZNF675/DAP/ |  | | | | | | | | | | | | | | | | | | | | | | | | | | | | | | | | | | | | | | | | | | | |
| mRNA splicing, via  spliceosome | 1.57E-03 | 2.81 | PPIL1/HNRNPH2/HNRNPA2B1/  PTBP1/ |  | | | | | | | | | | | | | | | | | | | | | | | | | | | | | | | | | | | | | | | | | | | |
| outflow tract morphogenesis | 1.58E-03 | 2.80 | HEY2/ |  | | | | | | | | | | | | | | | | | | | | | | | | | | | | | | | | | | | | | | | | | | | |
| positive regulation of  protein catabolic process | 1.58E-03 | 2.80 | IER3/ |  | | | | | | | | | | | | | | | | | | | | | | | | | | | | | | | | | | | | | | | | | | | |
| limb development | 1.64E-03 | 2.79 | NR2F2/MEOX2/ |  | | | | | | | | | | | | | | | | | | | | | | | | | | | | | | | | | | | | | | | | | | | |
| prostaglandin metabolic  process | 1.64E-03 | 2.79 | PTGR2/ |  | | | | | | | | | | | | | | | | | | | | | | | | | | | | | | | | | | | | | | | | | | | |
| mitochondrion morphogenesis | 1.64E-03 | 2.79 | COQ7/ |  | | | | | | | | | | | | | | | | | | | | | | | | | | | | | | | | | | | | | | | | | | | |
| energy reserve  metabolic process | 1.73E-03 | 2.76 | PRKAR1A/RAPGEF4/ |  | | | | | | | | | | | | | | | | | | | | | | | | | | | | | | | | | | | | | | | | | | | |
| positive regulation of  DNA replication | 1.87E-03 | 2.73 | GLI2/ |  | | | | | | | | | | | | | | | | | | | | | | | | | | | | | | | | | | | | | | | | | | | |
| aerobic respiration | 1.87E-03 | 2.73 | UQCRB/ |  | | | | | | | | | | | | | | | | | | | | | | | | | | | | | | | | | | | | | | | | | | | |
| positive regulation vascular  endothelial growth  factor production | 1.87E-03 | 2.73 | C3/ |  | | | | | | | | | | | | | | | | | | | | | | | | | | | | | | | | | | | | | | | | | | | |
| developmental growth | 1.88E-03 | 2.73 | GLI3/ZMIZ1/SMAD3/GLI2/ |  | | | | | | | | | | | | | | | | | | | | | | | | | | | | | | | | | | | | | | | | | | | |
| positive regulation of  stress fiber assembly | 1.88E-03 | 2.73 | RGCC/PFN1/SMAD3/ |  | | | | | | | | | | | | | | | | | | | | | | | | | | | | | | | | | | | | | | | | | | | |
| protein kinase B  signaling cascade | 1.88E-03 | 2.73 | EPHA2/ |  | | | | | | | | | | | | | | | | | | | | | | | | | | | | | | | | | | | | | | | | | | | |
| fibroblast growth factor  receptor signaling pathway | 1.91E-03 | 2.72 | PRKAR1A/EGF/HBEGF/CBL/FGF23/ |  | | | | | | | | | | | | | | | | | | | | | | | | | | | | | | | | | | | | | | | | | | | |
| regulation of transcription  from RNA polymerase II promoter | 2.06E-03 | 2.69 | PRKAR1A/INHBA/FSTL3/NR2F2/  ECM1/TEAD3/ |  | | | | | | | | | | | | | | | | | | | | | | | | | | | | | | | | | | | | | | | | | | | |
| neuron migration | 2.26E-03 | 2.65 | PRKG1/NAV1/NR2F2/NTRK3/ |  | | | | | | | | | | | | | | | | | | | | | | | | | | | | | | | | | | | | | | | | | | | |
| positive regulation of  sequence-specific DNA binding  transcription factor activity | 2.36E-03 | 2.63 | HMGN3/RGCC/ |  | | | | | | | | | | | | | | | | | | | | | | | | | | | | | | | | | | | | | | | | | | | |
| positive regulation of heart rate | 2.37E-03 | 2.62 | HEY2/ |  | | | | | | | | | | | | | | | | | | | | | | | | | | | | | | | | | | | | | | | | | | | |
| negative regulation of  interleukin-12 production | 2.37E-03 | 2.62 | C1QB/ |  | | | | | | | | | | | | | | | | | | | | | | | | | | | | | | | | | | | | | | | | | | | |
| regulation of GTPase activity | 2.37E-03 | 2.62 | PRKG1/ |  | | | | | | | | | | | | | | | | | | | | | | | | | | | | | | | | | | | | | | | | | | | |
| positive regulation of  insulin-like growth factor  receptor signaling pathway | 2.37E-03 | 2.62 | IGFBP5/ |  | | | | | | | | | | | | | | | | | | | | | | | | | | | | | | | | | | | | | | | | | | | |
| peroxisome organization | 2.41E-03 | 2.62 | PEX3/ |  | | | | | | | | | | | | | | | | | | | | | | | | | | | | | | | | | | | | | | | | | | | |
| positive regulation of  collagen biosynthetic process | 2.41E-03 | 2.62 | RGCC/ |  | | | | | | | | | | | | | | | | | | | | | | | | | | | | | | | | | | | | | | | | | | | |
| hydrogen peroxide  catabolic process | 2.41E-03 | 2.62 | TXNRD1/ |  | | | | | | | | | | | | | | | | | | | | | | | | | | | | | | | | | | | | | | | | | | | |
| positive regulation of  peptidyl-serine phosphorylation | 2.48E-03 | 2.61 | HAX1/NTRK3/ |  | | | | | | | | | | | | | | | | | | | | | | | | | | | | | | | | | | | | | | | | | | | |
| positive regulation of  protein kinase activity | 2.57E-03 | 2.59 | PRKAG1/CCR7/ |  | | | | | | | | | | | | | | | | | | | | | | | | | | | | | | | | | | | | | | | | | | | |
| negative regulation of  macrophage differentiation | 2.72E-03 | 2.57 | INHBA/C1QC/ |  | | | | | | | | | | | | | | | | | | | | | | | | | | | | | | | | | | | | | | | | | | | |
| regulation of myotube  differentiation | 2.72E-03 | 2.57 | RBM38/ |  | | | | | | | | | | | | | | | | | | | | | | | | | | | | | | | | | | | | | | | | | | | |
| translational initiation | 2.89E-03 | 2.54 | F5/ |  | | | | | | | | | | | | | | | | | | | | | | | | | | | | | | | | | | | | | | | | | | | |
| behavior | 2.98E-03 | 2.53 | S1PR2/ |  | | | | | | | | | | | | | | | | | | | | | | | | | | | | | | | | | | | | | | | | | | | |
| termination of RNA  polymerase I transcription | 3.06E-03 | 2.51 | TTF1/ |  | | | | | | | | | | | | | | | | | | | | | | | | | | | | | | | | | | | | | | | | | | | |
| negative regulation of  endopeptidase activity | 3.13E-03 | 2.50 | SLPI/SERPING1/ |  | | | | | | | | | | | | | | | | | | | | | | | | | | | | | | | | | | | | | | | | | | | |
| activation of cysteine-type  endopeptidase activity  involved in apoptotic process | 3.20E-03 | 2.50 | IFI27/SMAD3/DAP/ |  | | | | | | | | | | | | | | | | | | | | | | | | | | | | | | | | | | | | | | | | | | | |
| negative regulation of  viral genome replication | 3.30E-03 | 2.48 | RSAD2/BST2/SLPI/C3/IFITM3/ |  | | | | | | | | | | | | | | | | | | | | | | | | | | | | | | | | | | | | | | | | | | | |
| early endosome to late  endosome transport | 3.31E-03 | 2.48 | C3/FLNA/ |  | | | | | | | | | | | | | | | | | | | | | | | | | | | | | | | | | | | | | | | | | | | |
| cardiac muscle  tissue development | 3.31E-03 | 2.48 | PLN/HSPG2/ |  | | | | | | | | | | | | | | | | | | | | | | | | | | | | | | | | | | | | | | | | | | | |
| cyclooxygenase pathway | 3.31E-03 | 2.48 | PTGR1/ |  | | | | | | | | | | | | | | | | | | | | | | | | | | | | | | | | | | | | | | | | | | | |
| pathway-restricted SMAD  protein phosphorylation | 3.31E-03 | 2.48 | TGFB2/ |  | | | | | | | | | | | | | | | | | | | | | | | | | | | | | | | | | | | | | | | | | | | |
| negative regulation of  translation | 3.45E-03 | 2.46 | CIRBP/IGFBP5/ENC1/ |  | | | | | | | | | | | | | | | | | | | | | | | | | | | | | | | | | | | | | | | | | | | |
| positive regulation of  activation of membrane  attack complex | 3.55E-03 | 2.45 | C6/C3/ |  | | | | | | | | | | | | | | | | | | | | | | | | | | | | | | | | | | | | | | | | | | | |
| smoothened signaling  pathway involved in  ventral spinal cord  interneuron specification | 3.55E-03 | 2.45 | GLI3/GLI2/ |  | | | | | | | | | | | | | | | | | | | | | | | | | | | | | | | | | | | | | | | | | | | |
| smoothened signaling pathway  involved in spinal cord motor  neuron cell fate specification | 3.55E-03 | 2.45 | GLI3/GLI2/ |  | | | | | | | | | | | | | | | | | | | | | | | | | | | | | | | | | | | | | | | | | | | |
| tube development | 3.55E-03 | 2.45 | ITGB3/GLI2/ |  | | | | | | | | | | | | | | | | | | | | | | | | | | | | | | | | | | | | | | | | | | | |
| CD4-positive, alpha-beta  T cell activation | 3.55E-03 | 2.45 | RSAD2/STOML2/ |  | | | | | | | | | | | | | | | | | | | | | | | | | | | | | | | | | | | | | | | | | | | |
| positive regulation of  gene expression involved  in extracellular  matrix organization | 3.55E-03 | 2.45 | RGCC/SMAD3/ |  | | | | | | | | | | | | | | | | | | | | | | | | | | | | | | | | | | | | | | | | | | | |
| positive regulation  of type IIa hypersensitivity | 3.55E-03 | 2.45 | C3/ |  | | | | | | | | | | | | | | | | | | | | | | | | | | | | | | | | | | | | | | | | | | | |
| evasion or tolerance  of host defenses by virus | 3.55E-03 | 2.45 | SMAD3/ |  | | | | | | | | | | | | | | | | | | | | | | | | | | | | | | | | | | | | | | | | | | | |
| glycerol-3-phosphate  catabolic process | 3.55E-03 | 2.45 | GPD1L/ |  | | | | | | | | | | | | | | | | | | | | | | | | | | | | | | | | | | | | | | | | | | | |
| negative regulation  of calcium ion binding | 3.55E-03 | 2.45 | PLN/ |  | | | | | | | | | | | | | | | | | | | | | | | | | | | | | | | | | | | | | | | | | | | |
| G2/M transition of  mitotic cell cycle | 3.64E-03 | 2.44 | NEDD1/ARPP19/ |  | | | | | | | | | | | | | | | | | | | | | | | | | | | | | | | | | | | | | | | | | | | |
| positive regulation  of filopodium assembly | 3.82E-03 | 2.42 | FNBP1L/CCR7/ |  | | | | | | | | | | | | | | | | | | | | | | | | | | | | | | | | | | | | | | | | | | | |
| negative regulation  of DNA replication | 3.82E-03 | 2.42 | TTF1/ |  | | | | | | | | | | | | | | | | | | | | | | | | | | | | | | | | | | | | | | | | | | | |
| artery morphogenesis | 3.82E-03 | 2.42 | ZMIZ1/ |  | | | | | | | | | | | | | | | | | | | | | | | | | | | | | | | | | | | | | | | | | | | |
| post-embryonic development | 3.88E-03 | 2.41 | CCDC47/ |  | | | | | | | | | | | | | | | | | | | | | | | | | | | | | | | | | | | | | | | | | | | |
| response to nutrient | 3.89E-03 | 2.41 | COL1A1/ |  | | | | | | | | | | | | | | | | | | | | | | | | | | | | | | | | | | | | | | | | | | | |
| metanephros development | 3.92E-03 | 2.41 | GDF11/GLI3/ |  | | | | | | | | | | | | | | | | | | | | | | | | | | | | | | | | | | | | | | | | | | | |
| positive regulation of  neuron apoptotic process | 3.97E-03 | 2.40 | TGFB2/ATF4/ |  | | | | | | | | | | | | | | | | | | | | | | | | | | | | | | | | | | | | | | | | | | | |
| negative regulation of  sequence-specific DNA binding  transcription factor activity | 4.09E-03 | 2.39 | PRNP/FLNA/ |  | | | | | | | | | | | | | | | | | | | | | | | | | | | | | | | | | | | | | | | | | | | |
| sister chromatid cohesion | 4.48E-03 | 2.35 | SMC2/C3/ |  | | | | | | | | | | | | | | | | | | | | | | | | | | | | | | | | | | | | | | | | | | | |
| cAMP catabolic process | 4.48E-03 | 2.35 | PDE7A |  | | | | | | | | | | | | | | | | | | | | | | | | | | | | | | | | | | | | | | | | | | | |
| positive regulation of  ATPase activity | 4.48E-03 | 2.35 | C3/ |  | | | | | | | | | | | | | | | | | | | | | | | | | | | | | | | | | | | | | | | | | | | |
| insulin-like growth factor  receptor signaling pathway | 4.48E-03 | 2.35 | CRIM1/ |  | | | | | | | | | | | | | | | | | | | | | | | | | | | | | | | | | | | | | | | | | | | |
| fatty acid metabolic process | 4.60E-03 | 2.34 | C3/ |  | | | | | | | | | | | | | | | | | | | | | | | | | | | | | | | | | | | | | | | | | | | |
| salivary gland morphogenesis | 4.61E-03 | 2.34 | TGFB2/ |  | | | | | | | | | | | | | | | | | | | | | | | | | | | | | | | | | | | | | | | | | | | |
| regulation of fatty  acid biosynthetic process | 4.61E-03 | 2.34 | SCAP/ |  | | | | | | | | | | | | | | | | | | | | | | | | | | | | | | | | | | | | | | | | | | | |
| relaxation of cardiac muscle | 4.61E-03 | 2.34 | PLN/ |  | | | | | | | | | | | | | | | | | | | | | | | | | | | | | | | | | | | | | | | | | | | |
| mammary gland involution | 4.61E-03 | 2.34 | IGFBP5/ |  | | | | | | | | | | | | | | | | | | | | | | | | | | | | | | | | | | | | | | | | | | | |
| negative regulation of  osteoblast differentiation | 4.63E-03 | 2.33 | IGFBP5/SMAD3/FGF23/ |  | | | | | | | | | | | | | | | | | | | | | | | | | | | | | | | | | | | | | | | | | | | |
| negative regulation of  interferon-gamma production | 4.71E-03 | 2.33 | PRNP/C1QB/ |  | | | | | | | | | | | | | | | | | | | | | | | | | | | | | | | | | | | | | | | | | | | |
| regulation of angiogenesis | 4.71E-03 | 2.33 | EPHA2/ |  | | | | | | | | | | | | | | | | | | | | | | | | | | | | | | | | | | | | | | | | | | | |
| response to toxic substance | 4.78E-03 | 2.32 | PTGR1/GLYAT/ |  | | | | | | | | | | | | | | | | | | | | | | | | | | | | | | | | | | | | | | | | | | | |
| central nervous  system development | 5.09E-03 | 2.29 | COX7B/ |  | | | | | | | | | | | | | | | | | | | | | | | | | | | | | | | | | | | | | | | | | | | |
| G-protein coupled  receptor signaling pathway | 5.14E-03 | 2.29 | GPR116/RAPGEF4/GPR52/C3/S1PR2/CD97/CCR7/ |  | | | | | | | | | | | | | | | | | | | | | | | | | | | | | | | | | | | | | | | | | | | |
| JNK cascade | 5.15E-03 | 2.29 | TNFRSF19/PTGER4/ |  | | | | | | | | | | | | | | | | | | | | | | | | | | | | | | | | | | | | | | | | | | | |
| glycolysis | 5.20E-03 | 2.28 | PGK1/HK2/ |  | | | | | | | | | | | | | | | | | | | | | | | | | | | | | | | | | | | | | | | | | | | |
| negative regulation of  epithelial cell proliferation | 5.20E-03 | 2.28 | TGFB2/ |  | | | | | | | | | | | | | | | | | | | | | | | | | | | | | | | | | | | | | | | | | | | |
| neuron differentiation | 5.59E-03 | 2.25 | FZD8/EPHA2/ |  | | | | | | | | | | | | | | | | | | | | | | | | | | | | | | | | | | | | | | | | | | | |
| hematopoietic progenitor  cell differentiation | 5.91E-03 | 2.23 | INHBA/FSTL3/ |  | | | | | | | | | | | | | | | | | | | | | | | | | | | | | | | | | | | | | | | | | | | |
| regulation of  complement activation | 5.91E-03 | 2.23 | C3/C1QB/ |  | | | | | | | | | | | | | | | | | | | | | | | | | | | | | | | | | | | | | | | | | | | |
| myoblast fusion | 5.91E-03 | 2.23 | NEO1/ |  | | | | | | | | | | | | | | | | | | | | | | | | | | | | | | | | | | | | | | | | | | | |
| regulation of cardiac  muscle contraction  by regulation of the release  of sequestered calcium ion | 5.91E-03 | 2.23 | PLN/ |  | | | | | | | | | | | | | | | | | | | | | | | | | | | | | | | | | | | | | | | | | | | |
| bone development | 5.91E-03 | 2.23 | PTGER4/ |  | | | | | | | | | | | | | | | | | | | | | | | | | | | | | | | | | | | | | | | | | | | |
| positive regulation  of cell division | 5.92E-03 | 2.23 | TGFB2/ |  | | | | | | | | | | | | | | | | | | | | | | | | | | | | | | | | | | | | | | | | | | | |
| Phosphatidylinositol  -mediated signaling | 6.41E-03 | 2.19 | EGF/C3/HBEGF/NCS1/FGF23/ |  | | | | | | | | | | | | | | | | | | | | | | | | | | | | | | | | | | | | | | | | | | | |
| male gonad development | 6.41E-03 | 2.19 | C6/INHBA/FSTL3/ |  | | | | | | | | | | | | | | | | | | | | | | | | | | | | | | | | | | | | | | | | | | | |
| embryo development | 6.82E-03 | 2.17 | CCDC47/TGFB2/FZD8/FOXO6/ |  | | | | | | | | | | | | | | | | | | | | | | | | | | | | | | | | | | | | | | | | | | | |
|  |  |  |  |  | | | | | | | | | | | | | | | | | | | | | | | | | | | | | | | | | | | | | | | | | | | |
|  |  |  |  |  |  |  |  |  |  |  |  |  |  |  |  |  |  |  |  |  |  |  |  |  |  |  |  |  |  |  |  |  |  |  |  |  |  |  |  |  |  |  |  |  |  |  |  |

**Supplemental Table11** Pathway analysis: categories of AF-related gene in male

| **Pathway Name** | **p-value** | **-lgP** | **Gene** | | | | |  | | | | | | | | | | | | | | | | | | | | | | | |
| --- | --- | --- | --- | --- | --- | --- | --- | --- | --- | --- | --- | --- | --- | --- | --- | --- | --- | --- | --- | --- | --- | --- | --- | --- | --- | --- | --- | --- | --- | --- | --- |
| Metabolic pathways | 1.48E-41 | 40.83 | ACAT1/PGK1/UQCRB/ATP6V1D/UQCRHL/  HIBADH/NDUFB6/COX7C/COX17/NDUFA1/  NDUFA6/ASAH1/NDUFA8/COX5B/HK2/  UQCR10/PLA2G16/COX7B/TRIT1/IDH3B/  COQ7/ATP5O/ATP5G1/UROD/C3/  CDA/GGT5/NAGLU/PRPS1L1/ | | | | |  | | | | | | | | | | | | | | | | | | | | | | | |
| Huntington's disease | 2.35E-34 | 33.63 | UQCRB/UQCRHL/NDUFB6/COX7C/NDUFA1/  NDUFA6/NDUFA8/COX5B/UQCR10/VDAC3/  COX7B/ATP5O/ATP5G1/C3/CREB5/  CREBBP/ | | | | |  | | | | | | | | | | | | | | | | | | | | | | | |
| Parkinson's disease | 3.15E-34 | 33.50 | UQCRB/UQCRHL/NDUFB6/COX7C/NDUFA1/  NDUFA6/NDUFA8/COX5B/UQCR10/VDAC3/  COX7B/ATP5O/ATP5G1/C3/ | | | | |  | | | | | | | | | | | | | | | | | | | | | | | |
| Alzheimer's disease | 3.23E-34 | 33.49 | UQCRB/UQCRHL/NDUFB6/COX7C/NDUFA1/  NDUFA6/NDUFA8/COX5B/UQCR10/COX7B/  ATP5O/ATP5G1/ | | | | |  | | | | | | | | | | | | | | | | | | | | | | | |
| Oxidative phosphorylation | 1.40E-31 | 30.85 | UQCRB/ATP6V1D/UQCRHL/NDUFB6/COX7C/  COX17/NDUFA1/NDUFA6/NDUFA8/COX5B/  UQCR10/COX7B/ATP5O/ATP5G1/ | | | | |  | | | | | | | | | | | | | | | | | | | | | | | |
| Focal adhesion | 7.40E-24 | 23.13 | EGF/PIK3R3/COL1A1/COL3A1/COL1A2/  FLNA/ACTG1/ACTB/PXN/ITGB3/ | | | | |  | | | | | | | | | | | | | | | | | | | | | | | |
| Hypertrophic cardiomyopathy (HCM) | 9.63E-19 | 18.02 | PRKAG1/C3/ACTG1/TGFB2/TPM2/  ACTB/ITGB3/ | | | | |  | | | | | | | | | | | | | | | | | | | | | | | |
| Cardiac muscle contraction | 7.00E-18 | 17.15 | UQCRB/UQCRHL/COX7C/COX5B/UQCR10/  COX7B/TPM2/ | | | | |  | | | | | | | | | | | | | | | | | | | | | | | |
| Dilated cardiomyopathy | 8.17E-18 | 17.09 | PLN/C3/ACTG1/TGFB2/TPM2/  ACTB/ITGB3/ | | | | |  | | | | | | | | | | | | | | | | | | | | | | | |
| Valine, leucine and  isoleucine degradation | 1.87E-17 | 16.73 | ACAT1/HIBADH/ | | | | |  | | | | | | | | | | | | | | | | | | | | | | | |
| ECM-receptor interaction | 2.29E-17 | 16.64 | COL1A1/COL3A1/COL1A2/HSPG2/ITGB3/ | | | | |  | | | | | | | | | | | | | | | | | | | | | | | |
| Arrhythmogenic right ventricular  cardiomyopathy (ARVC) | 4.88E-16 | 15.31 | ACTG1/ACTB/ITGB3/ | | | | |  | | | | | | | | | | | | | | | | | | | | | | | |
| PI3K-Akt signaling pathway | 4.62E-15 | 14.34 | EGF/PIK3R3/COL1A1/COL3A1/COL1A2/  CREB5/ATF4/ITGB3/EPHA2/FGF23/ | | | | |  | | | | | | | | | | | | | | | | | | | | | | | |
| Amoebiasis | 1.53E-14 | 13.82 | PIK3R3/COL1A1/COL3A1/COL1A2/TGFB2/ | | | | |  | | | | | | | | | | | | | | | | | | | | | | | |
| Regulation of actin cytoskeleton | 2.63E-14 | 13.58 | EGF/PIK3R3/ACTG1/PFN1/ACTB/  PXN/ITGB3/FGF23/ | | | | |  | | | | | | | | | | | | | | | | | | | | | | | |
| Propanoate metabolism | 5.90E-14 | 13.23 | ACAT1/ | | | | |  | | | | | | | | | | | | | | | | | | | | | | | |
| Citrate cycle (TCA cycle) | 3.80E-13 | 12.42 | IDH3B/ | | | | |  | | | | | | | | | | | | | | | | | | | | | | | |
| Proteoglycans in cancer | 7.91E-13 | 12.10 | PIK3R3/FLNA/HSPG2/ACTG1/HBEGF/  TGFB2/FZD8/CBL/ACTB/PXN/  PLAU/ITGB3/FGF23/ | | | | |  | | | | | | | | | | | | | | | | | | | | | | | |
| Pyruvate metabolism | 7.05E-12 | 11.15 | ACAT1/ | | | | |  | | | | | | | | | | | | | | | | | | | | | | | |
| Malaria | 1.40E-11 | 10.85 | TGFB2/ | | | | |  | | | | | | | | | | | | | | | | | | | | | | | |
| Proteasome | 2.53E-11 | 10.60 | C6/SMC2/PSME2/PSMC5/ | | | | |  | | | | | | | | | | | | | | | | | | | | | | | |
| Fatty acid degradation | 2.53E-11 | 10.60 | ACAT1/CPT1A/ | | | | |  | | | | | | | | | | | | | | | | | | | | | | | |
| Cytokine-cytokine receptor interaction | 3.10E-11 | 10.51 | TNFRSF19/LIFR/EGF/INHBA/TGFB2/  CCR7/ | | | | |  | | | | | | | | | | | | | | | | | | | | | | | |
| Staphylococcus aureus infection | 4.04E-11 | 10.39 | HLA-DRB4/HLA-DMA/C3/C1QB/C1QC/ | | | | |  | | | | | | | | | | | | | | | | | | | | | | | |
| Influenza A | 2.20E-10 | 9.66 | RSAD2/HLA-DRB4/HLA-DMA/PIK3R3/ACTG1/  ACTB/CREBBP/ | | | | |  | | | | | | | | | | | | | | | | | | | | | | | |
| Phagosome | 2.38E-10 | 9.62 | ATP6V1D/HLA-DRB4/HLA-DMA/SEC22B/C3/  ACTG1/ACTB/ITGB3/ | | | | |  | | | | | | | | | | | | | | | | | | | | | | | |
| Leukocyte transendothelial  migration | 2.60E-10 | 9.59 | F5/RAPGEF4/PIK3R3/ACTG1/ACTB/  PXN/ | | | | |  | | | | | | | | | | | | | | | | | | | | | | | |
| Leishmaniasis | 5.21E-10 | 9.28 | HLA-DRB4/HLA-DMA/C3/TGFB2/ | | | | |  | | | | | | | | | | | | | | | | | | | | | | | |
| Rheumatoid arthritis | 5.86E-10 | 9.23 | ATP6V1D/HLA-DRB4/HLA-DMA/TGFB2/ | | | | |  | | | | | | | | | | | | | | | | | | | | | | | |
| Chagas disease  (American trypanosomiasis) | 8.30E-10 | 9.08 | PIK3R3/C3/TGFB2/C1QB/C1QC/  SMAD3/ | | | | |  | | | | | | | | | | | | | | | | | | | | | | | |
| Salmonella infection | 1.12E-09 | 8.95 | FLNA/ACTG1/PFN1/ACTB/ | | | | |  | | | | | | | | | | | | | | | | | | | | | | | |
| Hematopoietic cell lineage | 1.12E-09 | 8.95 | HLA-DRB4/ITGB3/ | | | | |  | | | | | | | | | | | | | | | | | | | | | | | |
| Pathways in cancer | 1.16E-09 | 8.93 | F5/EGF/PIK3R3/TGFB2/FZD8/  CBL/GLI3/SMAD3/GLI2/DAP/  FGF23/CREBBP/ | | | | |  | | | | | | | | | | | | | | | | | | | | | | | |
| HTLV-I infection | 1.96E-09 | 8.71 | VDAC3/HLA-DRB4/HLA-DMA/PIK3R3/C3/  TGFB2/FZD8/CRTC3/ATF4/SMAD3/  CREBBP/ | | | | |  | | | | | | | | | | | | | | | | | | | | | | | |
| Viral myocarditis | 2.48E-09 | 8.60 | HLA-DRB4/HLA-DMA/ACTG1/ACTB/ | | | | |  | | | | | | | | | | | | | | | | | | | | | | | |
| Pertussis | 3.14E-09 | 8.50 | C3/SERPING1/C1QB/C1QC/ | | | | |  | | | | | | | | | | | | | | | | | | | | | | | |
| Systemic lupus erythematosus | 7.29E-09 | 8.14 | C6/HLA-DRB4/HIST1H2BG/HLA-DMA/C3/  C1QB/C1QC/ | | | | |  | | | | | | | | | | | | | | | | | | | | | | | |
| Tuberculosis | 8.60E-09 | 8.07 | HLA-DRB4/HLA-DMA/C3/TGFB2/CREBBP/ | | | | |  | | | | | | | | | | | | | | | | | | | | | | | |
| Osteoclast differentiation | 2.30E-08 | 7.64 | PIK3R3/TGFB2/SIRPA/ITGB3/ | | | | |  | | | | | | | | | | | | | | | | | | | | | | | |
| Protein digestion and absorption | 4.72E-08 | 7.33 | COL1A1/COL3A1/COL1A2/ELN/XPNPEP2/ | | | | |  | | | | | | | | | | | | | | | | | | | | | | | |
| Endocytosis | 8.57E-08 | 7.07 | C6/EGF/TGFB2/CBL/SMAD3/  AGAP1/ | | | | |  | | | | | | | | | | | | | | | | | | | | | | | |
| Epstein-Barr virus infection | 8.57E-08 | 7.07 | C6/SMC2/HLA-DRB4/PIK3R3/PSMC5/  CREBBP/ | | | | |  | | | | | | | | | | | | | | | | | | | | | | | |
| Glycolysis / Gluconeogenesis | 1.57E-07 | 6.80 | PGK1/HK2/ | | | | |  | | | | | | | | | | | | | | | | | | | | | | | |
| Herpes simplex infection | 2.20E-07 | 6.66 | HLA-DRB4/HLA-DMA/C3/C1QB/CREBBP/ | | | | |  | | | | | | | | | | | | | | | | | | | | | | | |
| Vascular smooth muscle contraction | 2.95E-07 | 6.53 | PRKG1/ACTG2/ | | | | |  | | | | | | | | | | | | | | | | | | | | | | | |
| Estrogen signaling pathway | 3.70E-07 | 6.43 | PIK3R3/HBEGF/CREB5/ATF4/ | | | | |  | | | | | | | | | | | | | | | | | | | | | | | |
| Tryptophan metabolism | 1.08E-06 | 5.97 | ACAT1/ | | | | |  | | | | | | | | | | | | | | | | | | | | | | | |
| Hippo signaling pathway | 1.45E-06 | 5.84 | ACTG1/TGFB2/FZD8/ACTB/TEAD3/  SMAD3/GLI2/ | | | | |  | | | | | | | | | | | | | | | | | | | | | | | |
| Complement and coagulation cascades | 1.86E-06 | 5.73 | F5/C6/C3/SERPING1/C1QB/  C1QC/PLAU/ | | | | |  | | | | | | | | | | | | | | | | | | | | | | | |
| Salivary secretion | 2.08E-06 | 5.68 | PRKG1/ | | | | |  | | | | | | | | | | | | | | | | | | | | | | | |
| Hepatitis B | 2.32E-06 | 5.63 | VDAC3/PIK3R3/C3/HSPG2/TGFB2/  CREB5/ATF4/SMAD3/CREBBP/ | | | | |  | | | | | | | | | | | | | | | | | | | | | | | |
| TGF-beta signaling pathway | 2.63E-06 | 5.58 | INHBA/TGFB2/SMAD3/CREBBP/ | | | | |  | | | | | | | | | | | | | | | | | | | | | | | |
| PPAR signaling pathway | 2.68E-06 | 5.57 | AQP7/CPT1A/ | | | | |  | | | | | | | | | | | | | | | | | | | | | | | |
| HIF-1 signaling pathway | 4.31E-06 | 5.37 | PGK1/HK2/EGF/PIK3R3/CREBBP/ | | | | |  | | | | | | | | | | | | | | | | | | | | | | | |
| Legionellosis | 4.93E-06 | 5.31 | SEC22B/C3/ | | | | |  | | | | | | | | | | | | | | | | | | | | | | | |
| Ribosome | 9.31E-06 | 5.03 | MRPL15/ | | | | |  | | | | | | | | | | | | | | | | | | | | | | | |
| MAPK signaling pathway | 1.22E-05 | 4.91 | EGF/FLNA/TGFB2/ATF4/FGF23/ | | | | |  | | | | | | | | | | | | | | | | | | | | | | | |
| Peroxisome | 1.37E-05 | 4.86 | PEX3/ | | | | |  | | | | | | | | | | | | | | | | | | | | | | | |
| Insulin signaling pathway | 1.43E-05 | 4.84 | PRKAR1A/PRKAG1/HK2/PIK3R3/CBL/ | | | | |  | | | | | | | | | | | | | | | | | | | | | | | |
| Calcium signaling pathway | 2.09E-05 | 4.68 | PLN/VDAC3/C3/ | | | | |  | | | | | | | | | | | | | | | | | | | | | | | |
| Prion diseases | 2.76E-05 | 4.56 | C6/PRNP/C1QB/C1QC/ | | | | |  | | | | | | | | | | | | | | | | | | | | | | | |
| Tight junction | 2.85E-05 | 4.54 | ACTG1/ACTB/ | | | | |  | | | | | | | | | | | | | | | | | | | | | | | |
| Toxoplasmosis | 3.00E-05 | 4.52 | HLA-DRB4/HLA-DMA/PIK3R3/TGFB2/ | | | | |  | | | | | | | | | | | | | | | | | | | | | | | |
| Butanoate metabolism | 3.81E-05 | 4.42 | ACAT1/ | | | | |  | | | | | | | | | | | | | | | | | | | | | | | |
| Chemokine signaling pathway | 4.46E-05 | 4.35 | PIK3R3/PXN/CCR7/ | | | | |  | | | | | | | | | | | | | | | | | | | | | | | |
| Transcriptional misregulation  in cancer | 5.17E-05 | 4.29 | MLF1/PLAU/CEBPE/MAF/CCR7/ | | | | |  | | | | | | | | | | | | | | | | | | | | | | | |
| Alcoholism | 5.17E-05 | 4.29 | HIST1H2BG/CREB5/ATF4/ | | | | |  | | | | | | | | | | | | | | | | | | | | | | | |
| Lysine degradation | 6.30E-05 | 4.20 | ACAT1/ | | | | |  | | | | | | | | | | | | | | | | | | | | | | | |
| Toll-like receptor  signaling pathway | 9.73E-05 | 4.01 | PIK3R3/ | | | | |  | | | | | | | | | | | | | | | | | | | | | | | |
| Asthma | 1.33E-04 | 3.87 | HLA-DRB4/HLA-DMA/ | | | | |  | | | | | | | | | | | | | | | | | | | | | | | |
| Viral carcinogenesis | 1.41E-04 | 3.85 | VDAC3/HIST1H2BG/PIK3R3/C3/PXN/  CREB5/ATF4/CREBBP/ | | | | |  | | | | | | | | | | | | | | | | | | | | | | | |
| Insulin secretion | 1.42E-04 | 3.85 | RAPGEF4/CREB5/ATF4/ | | | | |  | | | | | | | | | | | | | | | | | | | | | | | |
| Bacterial invasion of epithelial cells | 1.76E-04 | 3.76 | PIK3R3/ACTG1/CBL/ACTB/PXN/ | | | | |  | | | | | | | | | | | | | | | | | | | | | | | |
| Gap junction | 1.80E-04 | 3.74 | EGF/PRKG1/GJD2/ | | | | |  | | | | | | | | | | | | | | | | | | | | | | | |
| Graft-versus-host disease | 1.85E-04 | 3.73 | HLA-DRB4/HLA-DMA/ | | | | |  | | | | | | | | | | | | | | | | | | | | | | | |
| Type I diabetes mellitus | 2.63E-04 | 3.58 | HLA-DRB4/HLA-DMA/ | | | | |  | | | | | | | | | | | | | | | | | | | | | | | |
| Cell adhesion molecules (CAMs) | 3.00E-04 | 3.52 | HLA-DRB4/HLA-DMA/NEO1/CD276/ | | | | |  | | | | | | | | | | | | | | | | | | | | | | | |
| Amino sugar and  nucleotide sugar metabolism | 3.68E-04 | 3.43 | HK2/ | | | | |  | | | | | | | | | | | | | | | | | | | | | | | |
| Circadian entrainment | 4.37E-04 | 3.36 | PRKG1/ | | | | |  | | | | | | | | | | | | | | | | | | | | | | | |
| Adherens junction | 4.65E-04 | 3.33 | ACTG1/ACTB/SMAD3/CREBBP/ | | | | |  | | | | | | | | | | | | | | | | | | | | | | | |
| Colorectal cancer | 5.02E-04 | 3.30 | PIK3R3/TGFB2/SMAD3/ | | | | |  | | | | | | | | | | | | | | | | | | | | | | | |
| Intestinal immune network  for IgA production | 5.05E-04 | 3.30 | HLA-DRB4/HLA-DMA/ | | | | |  | | | | | | | | | | | | | | | | | | | | | | | |
| Drug metabolism - cytochrome P450 | 5.25E-04 | 3.28 | FMO2/GSTT1/ | | | | |  | | | | | | | | | | | | | | | | | | | | | | | |
| Gastric acid secretion | 5.92E-04 | 3.23 | ACTB/ | | | | |  | | | | | | | | | | | | | | | | | | | | | | | |
| Glyoxylate and  dicarboxylate metabolism | 7.77E-04 | 3.11 | ACAT1/ | | | | |  | | | | | | | | | | | | | | | | | | | | | | | |
| Pancreatic cancer | 8.45E-04 | 3.07 | EGF/PIK3R3/TGFB2/SMAD3/ | | | | |  | | | | | | | | | | | | | | | | | | | | | | | |
| GnRH signaling pathway | 9.71E-04 | 3.01 | HBEGF/ATF4/ | | | | |  | | | | | | | | | | | | | | | | | | | | | | | |
| NF-kappa B signaling pathway | 9.71E-04 | 3.01 | PLAU/ | | | | |  | | | | | | | | | | | | | | | | | | | | | | | |
| Starch and sucrose metabolism | 1.04E-03 | 2.98 | HK2/ | | | | |  | | | | | | | | | | | | | | | | | | | | | | | |
| Ubiquinone and other  terpenoid-quinone biosynthesis | 1.08E-03 | 2.97 | COQ7/ | | | | |  | | | | | | | | | | | | | | | | | | | | | | | |
| Biosynthesis of amino acids | 1.36E-03 | 2.86 | PGK1/IDH3B/PRPS1L1/ | | | | |  | | | | | | | | | | | | | | | | | | | | | | | |
| Amphetamine addiction | 1.36E-03 | 2.86 | CREB5/ATF4/ | | | | |  | | | | | | | | | | | | | | | | | | | | | | | |
| Phenylalanine metabolism | 1.43E-03 | 2.84 | GLYAT/ | | | | |  | | | | | | | | | | | | | | | | | | | | | | | |
| Notch signaling pathway | 1.64E-03 | 2.78 | CREBBP/ | | | | |  | | | | | | | | | | | | | | | | | | | | | | | |
| RNA degradation | 1.71E-03 | 2.77 | C6/C3/PABPC4L/ | | | | |  | | | | | | | | | | | | | | | | | | | | | | | |
| Chronic myeloid leukemia | 1.91E-03 | 2.72 | PIK3R3/TGFB2/CBL/SMAD3/ | | | | |  | | | | | | | | | | | | | | | | | | | | | | | |
| Apoptosis | 2.35E-03 | 2.63 | PRKAR1A/PIK3R3/ | | | | |  | | | | | | | | | | | | | | | | | | | | | | | |
| Dopaminergic synapse | 2.60E-03 | 2.58 | CREB5/ATF4/ | | | | |  | | | | | | | | | | | | | | | | | | | | | | | |
| Galactose metabolism | 2.76E-03 | 2.56 | HK2/ | | | | |  | | | | | | | | | | | | | | | | | | | | | | | |
| Allograft rejection | 2.98E-03 | 2.53 | HLA-DRB4/HLA-DMA/ | | | | |  | | | | | | | | | | | | | | | | | | | | | | | |
| Circadian rhythm | 3.30E-03 | 2.48 | PRKAG1/ | | | | |  | | | | | | | | | | | | | | | | | | | | | | | |
| Purine metabolism | 3.32E-03 | 2.48 | PDE7A/PRPS1L1/CANT1/ | | | | |  | | | | | | | | | | | | | | | | | | | | | | | |
| Phosphatidylinositol signaling system | 4.26E-03 | 2.37 | PIK3R3/C3/ | | | | |  | | | | | | | | | | | | | | | | | | | | | | | |
| Cell cycle | 4.53E-03 | 2.34 | C3/TGFB2/SMAD3/CREBBP/ | | | | |  | | | | | | | | | | | | | | | | | | | | | | | |
| Carbohydrate digestion and absorption | 5.20E-03 | 2.28 | HK2/PIK3R3/ | | | | |  | | | | | | | | | | | | | | | | | | | | | | | |
| Melanoma | 5.83E-03 | 2.23 | EGF/PIK3R3/FGF23/ | | | | |  | | | | | | | | | | | | | | | | | | | | | | | |
| Long-term potentiation | 5.83E-03 | 2.23 | ATF4/CREBBP/ | | | | |  | | | | | | | | | | | | | | | | | | | | | | | |
| Wnt signaling pathway | 5.94E-03 | 2.23 | FZD8/SOST/SMAD3/CREBBP/ | | | | |  | | | | | | | | | | | | | | | | | | | | | | | |
| Prostate cancer | 8.53E-03 | 2.07 | EGF/PIK3R3/CREB5/ATF4/CREBBP/ | | | | |  | | | | | | | | | | | | | | | | | | | | | | | |
| Cocaine addiction | 9.54E-03 | 2.02 | CREB5/ATF4/ | | | | |  | | | | | | | | | | | | | | | | | | | | | | | |
| 2-Oxocarboxylic acid metabolism | 9.64E-03 | 2.02 | IDH3B | | | | |  | | | | | | | | | | | | | | | | | | | | | | | |
| Bladder cancer | 9.64E-03 | 2.02 | EGF/DAP/ | | | | |  | | | | | | | | | | | | | | | | | | | | | | | |
| Glycerophospholipid metabolism | 1.00E-02 | 2.00 | GPD1L/PLA2G16/ | | | | |  | | | | | | | | | | | | | | | | | | | | | | | |
| Glioma | 1.17E-02 | 1.93 | EGF/PIK3R3/ | | | | |  | | | | | | | | | | | | | | | | | | | | | | | |
| Renal cell carcinoma | 1.29E-02 | 1.89 | PIK3R3/TGFB2/CREBBP/ | | | | |  | | | | | | | | | | | | | | | | | | | | | | | |
| Metabolism of xenobiotics  by cytochrome P450 | 1.30E-02 | 1.89 | GSTT1/ | | | | |  | | | | | | | | | | | | | | | | | | | | | | | |
| Antigen processing and presentation | 1.52E-02 | 1.82 | HLA-DRB4/PSME2/HLA-DMA/LGMN/ | | | | |  | | | | | | | | | | | | | | | | | | | | | | | |
| Cholinergic synapse | 1.65E-02 | 1.78 | PIK3R3/CREB5/ATF4/ | | | | |  | | | | | | | | | | | | | | | | | | | | | | | |
| Ubiquitin mediated proteolysis | 1.66E-02 | 1.78 | C3/CBL/ | | | | |  | | | | | | | | | | | | | | | | | | | | | | | |
| Autoimmune thyroid disease | 1.78E-02 | 1.75 | HLA-DRB4/HLA-DMA/ | | | | |  | | | | | | | | | | | | | | | | | | | | | | | |
| Adipocytokine signaling pathway | 1.98E-02 | 1.70 | PRKAG1/CPT1A/ | | | | |  | | | | | | | | | | | | | | | | | | | | | | | |
| Vasopressin-regulated  water reabsorption | 2.22E-02 | 1.65 | CREB5/ | | | | |  | | | | | | | | | | | | | | | | | | | | | | | |
| ErbB signaling pathway | 2.37E-02 | 1.62 | EGF/PIK3R3/HBEGF/CBL/ | | | | |  | | | | | | | | | | | | | | | | | | | | | | | |
| mTOR signaling pathway | 2.57E-02 | 1.59 | PIK3R3/ | | | | |  | | | | | | | | | | | | | | | | | | | | | | | |
| Shigellosis | 2.80E-02 | 1.55 | ACTG1/PFN1/ACTB/ | | | | |  | | | | | | | | | | | | | | | | | | | | | | | |
| Measles | 2.88E-02 | 1.54 | PIK3R3/ | | | | |  | | | | | | | | | | | | | | | | | | | | | | | |
| Fructose and mannose metabolism | 3.36E-02 | 1.47 | HK2/ | | | | |  | | | | | | | | | | | | | | | | | | | | | | | |
| Glutamatergic synapse | 3.49E-02 | 1.46 | C3/ | | | | |  | | | | | | | | | | | | | | | | | | | | | | | |
| Fc gamma R-mediated phagocytosis | 3.54E-02 | 1.45 | PIK3R3/ | | | | |  | | | | | | | | | | | | | | | | | | | | | | | |
| Glutathione metabolism | 3.63E-02 | 1.44 | GSTT1/GGT5/ | | | | |  | | | | | | | | | | | | | | | | | | | | | | | |
| Aldosterone-regulated  sodium reabsorption | 4.64E-02 | 1.33 | PIK3R3/ | | | | |  | | | | | | | | | | | | | | | | | | | | | | | |
| Chemical carcinogenesis | 4.74E-02 | 1.32 | GSTT1/ | | | | |  | | | | | | | | | | | | | | | | | | | | | | | |
|  |  |  |  |  |  |  |  |  |  |  |  |  |  |  |  |  |  |  |  |  |  |  |  |  |  |  |  |  |  |  |  |

**Supplemental Table12** Fibrotic remodeling related genes

| **Gene** | **Gene Feature** | **Gender** | **Functional Classification** | **References** |
| --- | --- | --- | --- | --- |
| ADAMTS2 | Down | Male | Pro-fibrosis | [Hepatology.](http://www.ncbi.nlm.nih.gov/pubmed/17929299) 2007; 46(5):1620-31. |
| COL1A1 | Down | Male | Pro-fibrosis | [Circulation.](http://www.ncbi.nlm.nih.gov/pubmed/23459615) 2013 ;127(14):1466-75 |
| COL1A2 | Down | Male | Pro-fibrosis | [J Intern Med.](http://www.ncbi.nlm.nih.gov/pubmed/?term=COL1A2+and+atrial+fibrillation) 2012; 272(3):305-15. |
| COL3A1 | Down | Male | Pro-fibrosis | [Circulation.](http://www.ncbi.nlm.nih.gov/pubmed/23459615) 2013;127(14):1466-75 |
| ECM1 | Down | Male | Pro-fibrosis | [Hypertens Res.](http://www.ncbi.nlm.nih.gov/pubmed/22495609) 2012; 35(8):811-8. |
| FGF23 | Down | Male | Pro-fibrosis | [Mol Membr Biol.](http://www.ncbi.nlm.nih.gov/pubmed/24124751) 2013; 30(8):369-85. |
| FZD8 | Down | Male | Pro-fibrosis | [FASEB J.](http://www.ncbi.nlm.nih.gov/pubmed/26849959) 2016; 30(5):1823-35. |
| GLI3 | Down | Male | Pro-fibrosis | [Nat Commun.](http://www.ncbi.nlm.nih.gov/pubmed/27001906) 2016; 22; 7:10993. |
| HBEGF | Down | Male | Pro-fibrosis | PLoS One. 2012; 7(9):e44946. |
| INHBA | Down | Male | Pro-fibrosis | Wound Repair Regen. 2008; 16(2): 254- 65. |
| POSTN | Down | Male | Pro-fibrosis | J Cell Mol Med. 2015;19(10):2462-8. |
| P-SMAD3 | Down | Male | Pro-fibrosis | Cardiology. 2013;124(4):233-40. |
| TGFB2 | Down | Male | Pro-fibrosis | Sci Transl Med. 2014;17;6(267):267ra176. |
| TGF-βI | Down | Male | Pro-fibrosis | Cancer Lett. 2015; 356(200): 506–516. |
| WISP1 | Down | Male | Pro-fibrosis | Sci Rep. 2016 ;6:20547. |
| SOST | Down | Male | Anti-fibrosis | Ren Fail. 2015;37(9):1514-7. |
| TGF-βI | up | Female | Pro-fibrosis | Cancer Lett. 2015; 356(200): 506–516. |
| CMA1 | up | Female | Pro-fibrosis | Int J Clin Exp Pathol. 2014 ;15;7(7):3596-607. |
| P4HA1 | up | Female | Pro-fibrosis | J Hepatol. 2013 ;58(3):522-8. |
| BNIP3 | up | Female | Pro-fibrosis | Circ Heart Fail. 2013 ;6(3):572-83. |
| COL6A6 | up | Female | Pro-fibrosis | Biochim Biophys Acta. 2014 ;1842(9):1604-12. |
| POSTN | up | Female | Pro-fibrosis | J Cell Mol Med. 2015;19(10):2462-8. |
| PARP1 | up | Female | Pro-fibrosis | J Physiol Sci. 2015 ;65(1):105-11. |
| CCL18 | up | Female | Pro-fibrosis | Eur Respir J. 2014 ;44(6):1608-15. |
| THBS1 | Down | Female | Pro-fibrosis | J Vet Intern Med. 2014 ;28(6):1666-75. |
| IL6 | Down | Female | Pro-fibrosis | J Immunol. 2014;193(7):3755-68. |
| IL1B | Down | Female | Pro-fibrosis | Autophagy. 2015;11(8):1280-92. |
| EGR1 | Down | Female | Pro-fibrosis | Am J Cancer Res. 2016 Jun 1;6(6):1358-70. |
| IL8 | Down | Female | Pro-fibrosis | Hum Genet. 2016 Aug;135(8):881-94. |
| TGFB2 | Down | Female | Pro-fibrosis | Sci Transl Med. 2014;17;6(267):267ra176. |
| ID1 | up | Female | Anti-fibrosis | Biochem Biophys Res Commun. 2014;444(1):81-5. |
| NR4A1 | Down | Female | Anti-fibrosis | Nat Med. 2015 Feb;21(2):150-8. |
| KLF2 | Down | Female | Anti-fibrosis | Gut. 2015 ;64(9):1349-50. |
| AXIN2 | Down | Female | Anti-fibrosis | Eur Surg Res. 2015;55(4):328-340. |
| SOST | Down | Female | Anti-fibrosis | Ren Fail. 2015;37(9):1514-7. |
| KLF4 | Down | Female | Anti-fibrosis | Front Physiol. 2015 Nov 12;6:327. |
